# Proportion of pregnant women with HBV infection eligible for antiviral prophylaxis to prevent vertical transmission: A systematic review and meta-analysis

**DOI:** 10.1016/j.jhepr.2024.101064

**Published:** 2024-03-26

**Authors:** Hugues Delamare, Julian Euma Ishii-Rousseau, Adya Rao, Mélanie Cresta, Jeanne Perpétue Vincent, Olivier Ségéral, Shevanthi Nayagam, Yusuke Shimakawa

**Affiliations:** 1 Institut Pasteur, Université Paris Cité, Unité d'Épidémiologie des Maladies Émergentes, Paris, France; 2 Department of Global Health Promotion, Tokyo Medical and Dental University, Tokyo, Japan; 3 MRC Centre for Infectious Disease Analysis, School of Public Health, Imperial College London, UK; 4 Unité VIH/Sida, Service des maladies infectieuses, Hôpitaux Universitaires de Genève, Genève, Switzerland

**Keywords:** Hepatitis B, Elimination, Mother-to-child transmission, Pregnant women, HBV DNA, HBeAg, Systematic review

## Abstract

**Background & Aims:**

In 2020, the World Health Organization (WHO) recommended peripartum antiviral prophylaxis (PAP) for pregnant women infected with hepatitis B virus (HBV) with high viremia (≥200,000 IU/ml). Hepatitis B e antigen (HBeAg) was also recommended as an alternative when HBV DNA is unavailable. To inform policymaking and guide the implementation of prevention of mother-to-child transmission strategies, we conducted a systematic review and meta-analysis to estimate the proportion of HBV-infected pregnant women eligible for PAP at global and regional levels.

**Methods:**

We searched PubMed, EMBASE, Scopus, and CENTRAL for studies involving HBV-infected pregnant women. We extracted proportions of women with high viremia (≥200,000 IU/ml), proportions of women with positive HBeAg, proportions of women cross-stratified based on HBV DNA and HBeAg, and the risk of child infection in these maternal groups. Proportions were pooled using random-effects meta-analysis.

**Results:**

Of 6,999 articles, 131 studies involving 71,712 HBV-infected pregnant women were included. The number of studies per WHO region was 66 (Western Pacific), 21 (Europe), 17 (Africa), 11 (Americas), nine (Eastern Mediterranean), and seven (South-East Asia). The overall pooled proportion of high viremia was 21.27% (95% CI 17.77–25.26%), with significant regional variation: Western Pacific (31.56%), Americas (23.06%), Southeast Asia (15.62%), Africa (12.45%), Europe (9.98%), and Eastern Mediterranean (7.81%). HBeAg positivity showed similar regional variation. After cross-stratification, the proportions of high viremia and positive HBeAg, high viremia and negative HBeAg, low viremia and positive HBeAg, and low viremia and negative HBeAg were 15.24% (95% CI 11.12–20.53%), 2.70% (95% CI 1.88–3.86%), 3.69% (95% CI 2.86–4.75%), and 75.59% (95% CI 69.15–81.05%), respectively. The corresponding risks of child infection following birth dose vaccination without immune globulin and PAP were 14.86% (95% CI 8.43–24.88%), 6.94% (95% CI 2.92–15.62%), 7.14% (95% CI 1.00–37.03%), and 0.14% (95% CI 0.02–1.00%).

**Conclusions:**

Approximately 20% of HBV-infected pregnant women are eligible for PAP. Given significant regional variations, each country should tailor strategies for HBsAg screening, risk stratification, and PAP in routine antenatal care.

**Impact and implications:**

In 2020, the WHO recommended that pregnant women who test positive for the hepatitis B surface antigen (HBsAg) undergo HBV DNA testing or HBeAg and those with high viremia (≥200,000 IU/ml) or positive HBeAg receive PAP. To effectively implement new HBV PMTCT interventions and integrate HBV screening, risk stratification, and antiviral prophylaxis into routine antenatal care services, estimating the proportion of HBV-infected pregnant women eligible for PAP is critical. In this systematic review and meta-analysis, we found that approximately one-fifth of HBV-infected pregnant women are eligible for PAP based on HBV DNA testing, and a similar proportion is eligible based on HBeAg testing. Owing to substantial regional variations in eligibility proportions and the availability and costs of different tests, it is vital for each country to optimize strategies that integrate HBV screening, risk stratification, and PAP into routine antenatal care services.

**Systematic review registration:**

This study was registered with PROSPERO (Protocol No: CRD42021266545).

## Highlights

• Overall proportion of pregnant women eligible for PAP was 21.27% based on high HBV DNA levels and 23.86% based on positive HBeAg.
• Significant regional variation was observed for the pooled proportion with high viremia.
• After cross-stratification, 15.24% had high viremia and positive HBeAg, 2.70% high viremia and negative HBeAg, 3.69% low viremia and positive HBeAg, and 75.59% low viremia and negative HBeAg.
• From these subgroups, the risks of child infection following birth dose vaccination without immune globulin and PAP were 14.86%, 6.94%, 7.14%, and 0.14%, respectively.

## Graphical abstract

**Figure:**
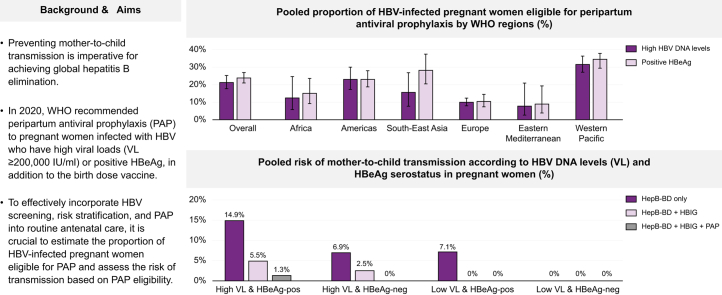

## Introduction

Globally, 316 million individuals are living with chronic hepatitis B virus (HBV) infection,^1^ of whom 95% reside in low- and middle-income countries (LMICs).^2^ An estimated 820,000 annual deaths are attributed to HBV-related cirrhosis or hepatocellular carcinoma.^3^ The World Health Organization (WHO) has set a goal to globally eliminate HBV infection as a public health threat by 2030, including achieving a 0.1% prevalence of HBsAg in children aged 5 years.^3^ Preventing mother-to-child transmission (MTCT) of HBV is crucial in reaching this goal, as this mode of transmission is a major risk factor for chronic HBV infection and related liver diseases, compared with horizontal transmission later in life.^4^

The WHO has recommended since 2009 that all infants should receive a series of three to four doses of hepatitis B vaccine starting immediately after birth, preferably within 24 h, to prevent perinatal MTCT and early horizontal transmission.^5^ Moreover, to further reduce the MTCT risk, the WHO published in 2020 its first guidelines for peripartum antiviral prophylaxis (PAP), recommending that pregnant women who test positive for HBsAg and have HBV DNA levels of 200,000 IU/ml or greater should receive tenofovir prophylaxis from at least the 28th week of pregnancy until birth.^5^ The use of HBeAg testing, as an alternative means of determining eligibility for tenofovir prophylaxis, was also recommended for pregnant women with limited access to quantitative HBV DNA testing.^5^^,^^6^

To effectively implement prevention of MTCT (PMTCT) interventions and integrate HBV screening, risk stratification, and antiviral prophylaxis into routine antenatal care services, it is essential to estimate the proportion of HBV-infected pregnant women who are eligible for peripartum antiviral prophylaxis.^5^^,^^7^ In LMICs, where most HBV infections occur, understanding regional differences in this proportion can help optimize resource allocation for this critical intervention. To address these issues, we conducted a systematic review and meta-analysis to estimate the following: (Q1) the proportion of HBV-infected pregnant women with high HBV DNA levels (≥200,000 IU/ml), (Q2) the proportion of HBV-infected pregnant women who test positive for HBeAg, (Q3) the proportion of HBV-infected pregnant women classified into four subgroups based on their HBeAg serostatus and HBV DNA levels (HBeAg-positive with high viremia, HBeAg-positive with low viremia, HBeAg-negative with high viremia, and HBeAg-negative with low viremia), and (Q4) the risk of child infection in each of the four maternal subgroups.

## Materials and methods

### Search strategy and selection criteria

We searched PubMed, EMBASE, Scopus, and CENTRAL for studies published between January 1, 2000, and June 22, 2021, without any language restrictions. The search strategy used the terms ‘hepatitis B infection’ AND (‘viral load’ OR ‘HBeAg’) AND ‘pregnancy’ and their variations (Supplementary Methods 1). References for included studies were used to manually identify additional studies.

We included studies evaluating HBV DNA levels and/or HBeAg serostatus, anytime during pregnancy, in HBsAg-positive pregnant women who did not receive any anti-HBV therapy at the baseline assessment. We accepted studies providing antiviral therapy to these women after the baseline assessment. We excluded studies that selected pregnant women based on their HBeAg status or viral load, as well as those with fewer than 10 participants for a given question.

Eligibility criteria were developed for each of the four questions. To address Q1, studies were required to report the proportion of pregnant women with HBV DNA levels equal to or greater than 200,000 IU/ml (≥5.3 log IU/ml). However, as not all studies used this threshold, we also accepted studies that dichotomized viral loads into high and low categories using a threshold ranging from 100,000 (5.0 log) IU/ml to 1,000,000 (6.0 log) IU/ml. For Q2, studies were included if the proportion of pregnant women with positive HBeAg was available. For Q3, studies reporting the number of women in a subgroup defined by both HBV DNA levels (high or low, at a threshold range of 100,000–1,000,000 IU/ml) and HBeAg serostatus (positive or negative) were included. For Q4, studies reporting the risk of child infection in each of the four maternal categories defined above were included. Child infection was defined based on HBsAg positivity in infants aged between 6 and 12 months.^5^ However, for infants who received at least three doses of hepatitis B vaccine, the definition was expandable up to 24 months because of the negligible risk of horizontal transmission in these children.^8^ Corresponding authors were contacted when critical information was missing.

Titles and abstracts of all articles identified through the literature search were independently screened by two reviewers (MC and AR). This was followed by a full-text review and data extraction using a pre-piloted sheet (Supplementary Methods 2) by two additional independent reviewers (HD and JEIR). Any discrepancies were resolved by YS. Extracted data included study setting, study design, recruitment period, maternal and infant HBV markers (type and timing of sampling, as well as assay type), number of participants, their demographics and characteristics, and administration of maternal antiviral prophylaxis or infant immunoprophylaxis. Viral loads reported as copies/ml were converted to IU/ml.^5^ When we encountered articles reporting overlapping populations and settings, a main study was selected based on completeness and relevance to the eligibility criteria, whereas other overlapping studies were discounted. However, the inclusion of Chinese-language articles presented additional challenges. Patient groups reported in these articles often appeared in English-language articles as well, making it difficult to determine their uniqueness.^9^ We therefore made the decision not to consider Chinese-language articles in our systematic review. The risk of bias was assessed using a tool developed by Hoy *et al.*^10^ for the first three questions (Supplementary Methods 3) and the Altman^11^ framework for the fourth question (Supplementary Methods 4). The protocol was preregistered in PROSPERO (CRD42021266545). The study was reported according to the PRISMA guidelines.

### Data analysis

The meta-analysis was conducted using the ‘metaprop’ command with RStudio version 3.3.0+ (PBC, Boston, MA, USA). Proportions, specific to each WHO region, were pooled via a random-effects meta-analysis using a generalized linear mixed model with a logit link approach. The percentage of heterogeneity was evaluated using the *I*^2^ statistic. To explore the sources of heterogeneity for Q1 and Q2, subgroup analyses were performed on median or mean maternal age, maternal coinfection with HIV, study design, recruitment site, median recruitment year, whether the HBsAg screening process was fully described or not, and the uptake rate for HBV DNA quantification or HBeAg testing. In addition, the viral load cut-off used in each study was assessed for Q1, and the type of HBeAg assay was assessed for Q2. For Q4, the risk of child infection was stratified by the administration of PAP and infant immunoprophylaxis (hepatitis B birth dose vaccine (HepB-BD) and/or hepatitis B immune globulin (HBIG)). Heterogeneity between subgroups was assessed using the meta-regression and test of moderators. Two-sided *p* <0.05 was considered as statistically significant. Small-study effects were visually assessed by plotting study size against the logarithm of the odds of proportion.^12^

## Results

**Fig. 1:**
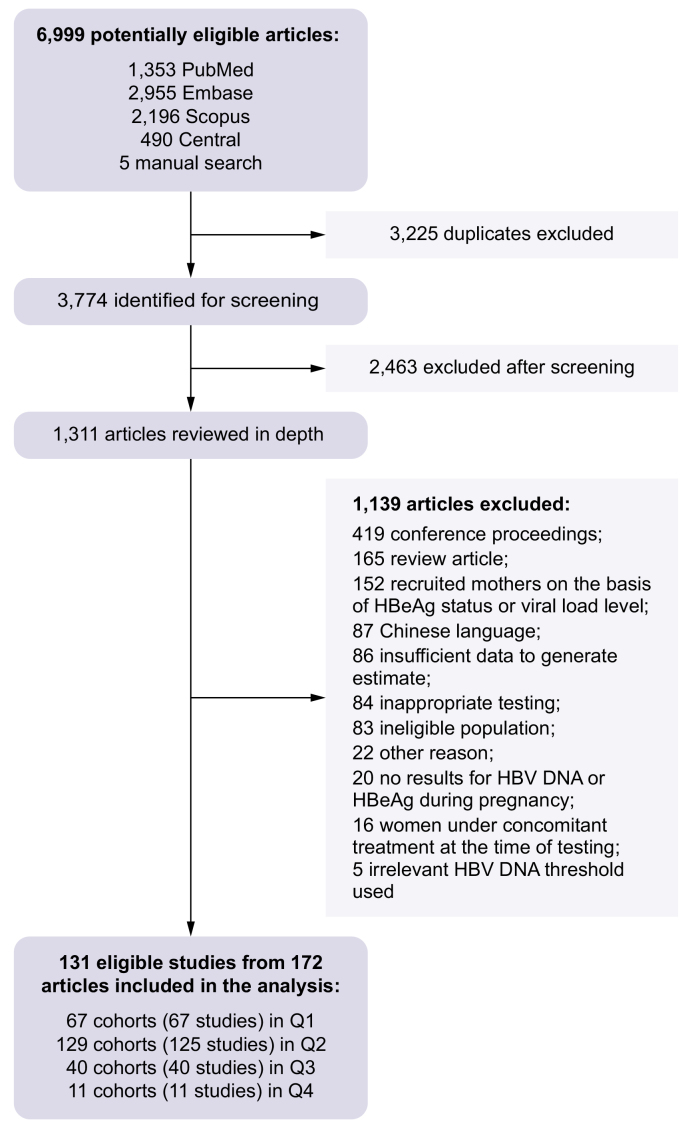
Flow diagram of study selection.

Of 6,999 articles identified, 1,311 underwent full-text assessment. Finally, 131 distinct studies reported in 172 articles met the inclusion criteria and provided data for 71,712 women who were HBsAg-positive (Fig. 1 and Supplementary References 1 and Supplementary Results 1). Notably, four studies reported two distinct groups each – either because some women had HIV co-infection (three studies)[13], [14], [15] or they were monitored differently (one study).^16^ This gave us a total of 135 cohorts for meta-analysis. The numbers of cohorts and studies evaluated in each of the questions were as follows: 67 cohorts from 67 studies for Q1, 129 cohorts from 125 studies for Q2, 40 cohorts from 40 studies for Q3, and 11 cohorts from 11 studies for Q4 (Supplementary References 1). Study characteristics are presented in Supplementary Results 2. The majority of the studies were conducted in the WHO Western Pacific Region (WPR: n = 66, 50%), especially in China (n = 48, 36%), followed by the European Region (EUR: n = 21, 16%), African Region (AFR: n = 17, 13%), Regions of the Americas (AMR: n = 11, 8%), Eastern Mediterranean Region (EMR: n = 9, 7%), and South-East Asia Region (SEAR: n = 7, 5%). Study designs were prospective (n = 65, 48%), retrospective (n = 38, 29%), or cross-sectional (n = 28, 22%). Apart from 11 studies that did not specify the type of assays used to quantify HBV DNA levels, all the studies used PCR assays. HBeAg was detected using enzyme immunoassay (n = 58, 45%), chemiluminescent immunoassay (n = 32, 24%), fluorescent immunoassay (n = 4, 3%), or a rapid diagnostic test (n = 5, 4%). In 30 studies (23%), the method of HBeAg detection was not reported.

The assessment of risk of bias is summarized in Supplementary Results 3. Regarding the representativeness of the study participants, the majority of the studies recruited women from tertiary centers (n = 94, 72%), whereas a smaller proportion recruited from primary care settings (n = 34, 26%). Sixty-two studies (47%) provided a comprehensive description of the HBsAg screening process, indicating a reduced susceptibility to selection bias. With respect to the uptake of HBV DNA quantification in women who were HBsAg-positive, 25 studies (36%) reported an uptake of ≥75%, 24 studies (36%) reported an uptake of <75%, and 18 studies (27%) did not provide the uptake information. The uptake was relatively higher for HBeAg testing: 57 studies (46%) reported an uptake of ≥75%, 27 studies (22%) reported an uptake of <75%, and 41 studies (33%) did not report the uptake information. The adapted funnel plots did not show any clear asymmetry (Supplementary Results 4), suggesting the lack of small-study effects.

**Fig. 2:**
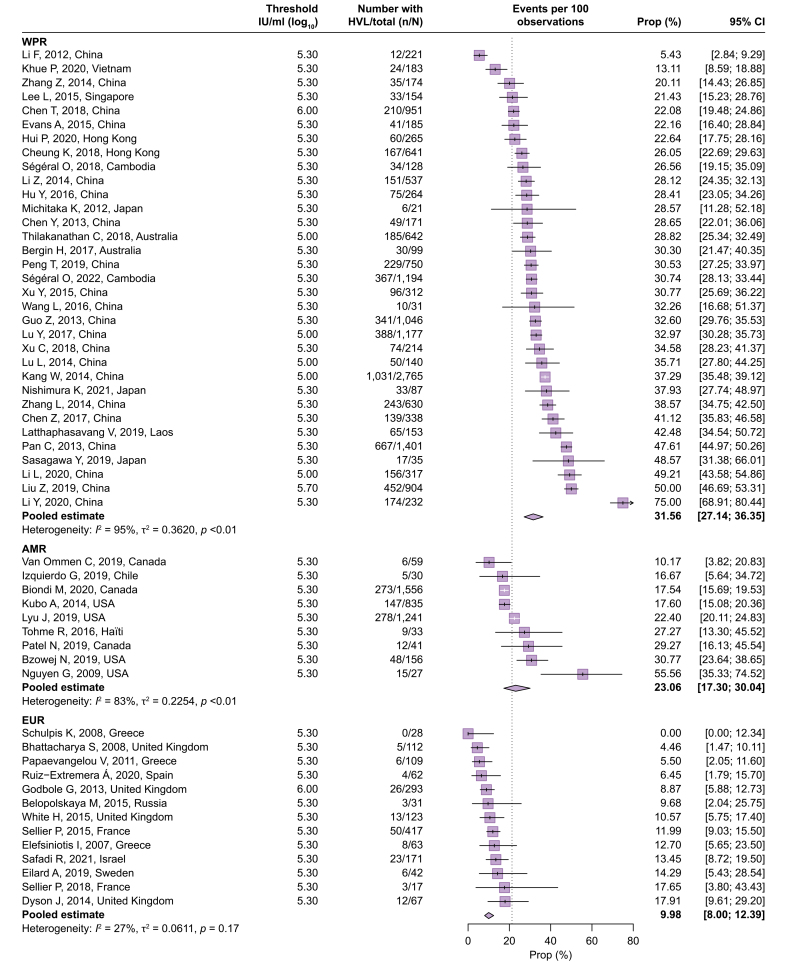

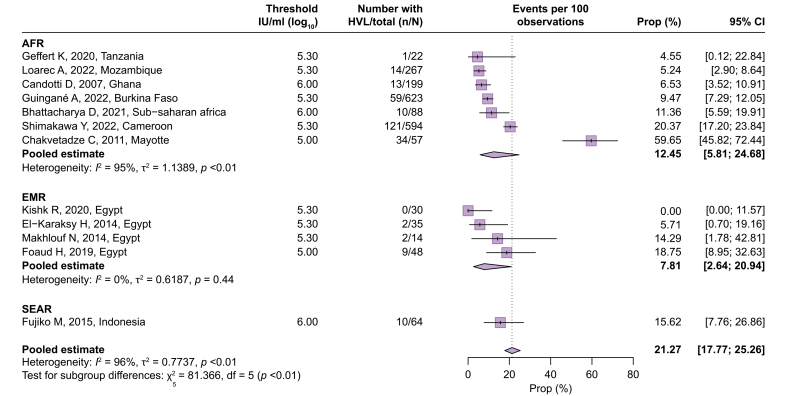
Proportion of pregnant women with HBV infection with high HBV DNA levels. Proportions were pooled via a random-effects meta-analysis using a generalized linear mixed model with a logit link approach. AFR, African Region; AMR, Regions of the Americas; EMR, Eastern Mediterranean Region; EUR, European Region; SEAR, South-East Asia Region; WPR, Western Pacific Region.

Fig. 2 presents the proportion of women with high HBV DNA levels among 23,881 pregnant women with chronic HBV infection, derived from 67 cohorts (Q1). The overall pooled estimate was 21.27% (95% CI 17.77–25.26%), accompanied by considerable heterogeneity across the studies (*I*^2^ = 96%). Significant variation was observed according to the WHO regions (test of moderators, *p* <0.0001), with the WPR having the highest proportion at 31.56% (95% CI 27.14–36.35%, *I*^2^ = 95%). The AMR accounted for 23.06% (95% CI 17.30–30.04%, *I*^2^ = 83%), making it the second-highest region, likely because of the inclusion of studies focusing on Asian communities in North America.[17], [18], [19], [20] After excluding these four studies, the pooled estimate in the AMR was 17.51% (95% CI 16.07–19.04%, *I*^2^ = 6%). For the rest of the world, the proportion was comparatively low when considered in relation to the WPR: AFR (12.45%, 95% CI 5.81–24.68%, *I*^2^ = 95%), EUR (9.98%, 95% CI 8.00–12.39%, *I*^2^ = 27%), and EMR (7.81%, 95% CI 2.64–20.94%, *I*^2^ = 0%). Only one study from the SEAR was available for this analysis (15.62%, 95% CI 7.76–26.86%).^21^ Outliers were identified, and their potential reasons are presented in Supplementary Results 5. Of four studies showing extremely lower estimates (<5%), three included a small number of participants (n <50).[22], [23], [24] All of five studies showing extremely higher estimates (>50% for the WPR and >30% for the rest of the world) recruited participants at specialized tertiary care centers.^18^^,^^19^^,^[25], [26], [27]

Owing to the large number of included studies in the WPR and the higher proportion of women with high HBV DNA levels in this region, subgroup analyses were conducted after stratifying the data into the WPR and the rest of the world (Table S1). The use of a higher threshold for HBV DNA levels was associated with a lower proportion of women surpassing these thresholds in the other regions (test of moderators, *p* = 0.0217), whereas such an association was not observed in the WPR. Studies providing a comprehensive description of the HBsAg screening process tended to yield lower estimates in both the WPR and the other regions (test of moderators, *p* <0.0001 and *p* = 0.0675, respectively).

**Fig. 3:**
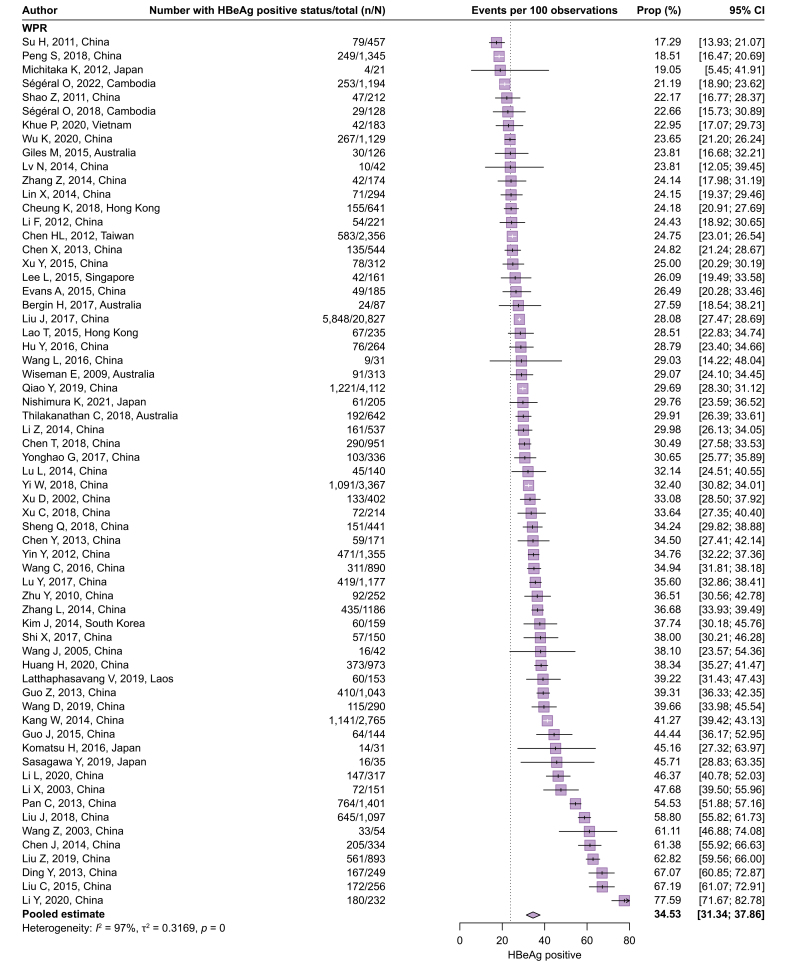

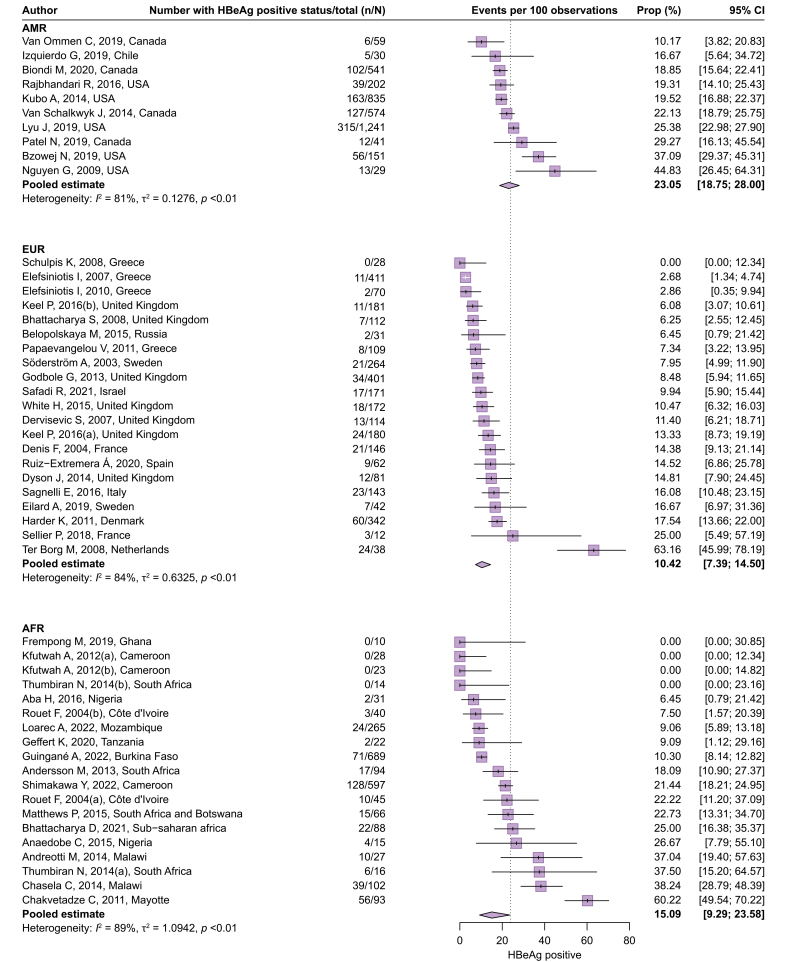

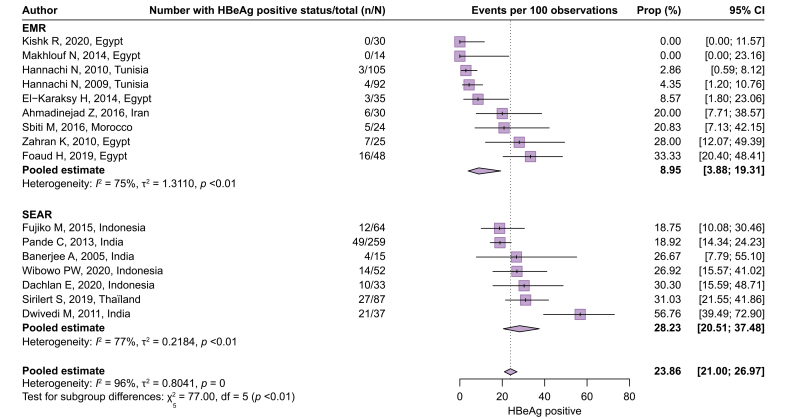
Proportion of pregnant women with HBV infection who tested positive for HBeAg. Proportions were pooled via a random-effects meta-analysis using a generalized linear mixed model with a logit link approach. AFR, African Region; AMR, Regions of the Americas; EMR, Eastern Mediterranean Region; EUR, European Region; SEAR, South-East Asia Region; WPR, Western Pacific Region.

Fig. 3 presents the proportion who test positive for HBeAg in 68,662 HBV-infected pregnant women from 129 cohorts (Q2). The overall pooled estimate was 23.86% (95% CI 21.00–26.97%). Similar to the proportion of women with high viremia, considerable heterogeneity was observed across the studies (*I*^2^ = 96%) and across the WHO regions (test of moderators, *p* <0.0001). The proportion was the highest in the WPR (34.53%, 95% CI 31.34–37.86%), followed by the SEAR (28.23%, 95% CI 20.51–37.48%), the AMR (23.05%, 95% CI 18.75–28.00%), the AFR (15.09%, 95% CI 9.29–23.58%), the EUR (10.42%, 95% CI 7.39–14.50%), and the EMR (8.95%, 95% CI 3.88–19.31%). The characteristics of outlier cohorts are summarized in Supplementary Results 5. Subgroup analyses of the proportion of HBV-infected women who test positive for HBeAg are presented in Table S2. In the WPR, a lower age of women was significantly associated with a higher proportion of positive HBeAg (39.65%, 95% CI 31.97–47.87%) in studies with a median/mean age of <29 years compared with 29.38% (95% CI 25.78–33.26%) in studies with ≥29 years (*p* = 0.0154). This association was not confirmed in other WHO regions. Of the potential sources of methodological heterogeneity, there was strong evidence for the study design in other regions (*p* = 0.0264) and whether HBsAg screening process fully described or not in the WPR (*p* = 0.0397).

**Fig. 4:**
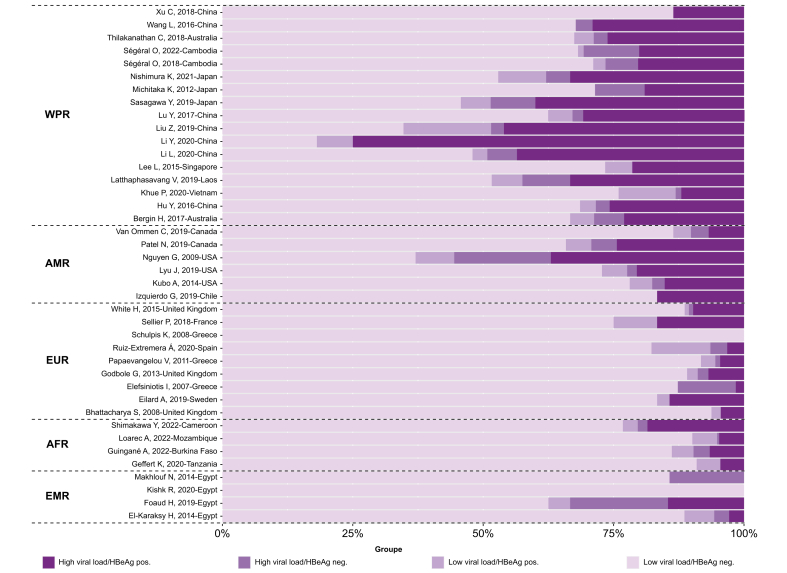
Proportion of pregnant women with HBV infection in subgroups defined by both HBeAg status and HBV DNA levels. Proportions were pooled via a random-effects meta-analysis using a generalized linear mixed model with a logit link approach. AFR, African Region; AMR, Regions of the Americas; EMR, Eastern Mediterranean Region; EUR, European Region; WPR, Western Pacific Region.

Fig. 4 presents the distribution of 10,386 HBV-infected pregnant women, from 39 cohorts, into the four subgroups defined by both HBV DNA levels and HBeAg serostatus (Q3). The overall pooled estimates of the proportion with high viremia and positive HBeAg, high viremia and negative HBeAg, low viremia and positive HBeAg, and low viremia and negative HBeAg were 15.24% (95% CI 11.12–20.53%, *I*^2^ = 95%), 2.70% (95% CI 1.88–3.86%, *I*^2^ = 83%), 3.69% (95% CI 2.86–4.75%, *I*^2^ = 86%), and 75.59% (95% 69.15–81.05%, *I*^2^ = 96%), respectively. In the WPR, the proportion with high viremia and positive HBeAg was relatively high, whereas in the other regions, the vast majority were in a subgroup of low viremia and negative HBeAg (Fig. S1). Only the minority represented the high viremia and HBeAg negative group and the low viremia and HBeAg positive group across the regions.

**Fig. 5:**
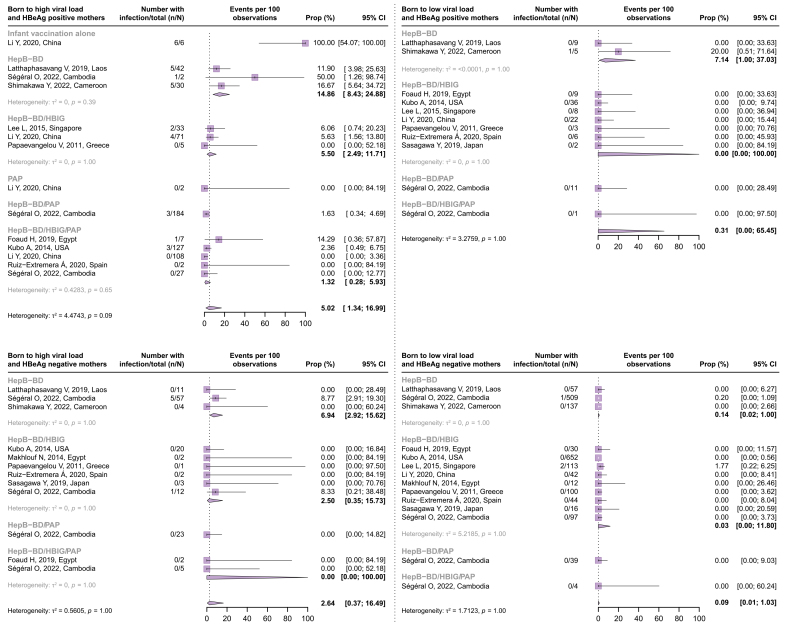
Risk of child infection in subgroups defined by maternal HBeAg status and HBV DNA levels. Proportions were pooled via a random-effects meta-analysis using a generalized linear mixed model with a logit link approach. HBIG, hepatitis B immune globulin; HepB-BD, hepatitis B birth dose vaccine; PAP, peripartum antiviral prophylaxis.

Fig. 5 presents the risk of child infection stratified by both maternal HBV markers and maternal and child prophylaxis administered (Q4). The majority of the cohorts provided one of the following combinations of the maternal/infant immunoprophylaxis: ‘HepB-BD only’, ‘HepB-BD + HBIG’, and ‘HepB-BD + HBIG + PAP’. The risk of child infection following ‘HepB-BD only’, ‘HepB-BD + HBIG’, and ‘HepB-BD + HBIG + PAP’ was 14.86% (95% CI 8.43–24.88%), 5.50% (95% CI 2.49–11.71%), and 1.32% (95% CI 0.28–5.93%) from women who were HBeAg-positive with high viremia, respectively; 6.94% (95% CI 2.92–15.62%), 2.50% (95% CI 0.35–15.73%), and 0.00% (95% CI 0.00–100.00%) from women who were HBeAg-negative with high viremia, respectively; 7.14% (95% CI 1.00–37.03%), 0.00% (95% CI 0.00–100.00%), and 0.00% (95% CI 0.00–97.50%) from women who were HBeAg-positive with low viremia, respectively; and 0.14% (95% CI 0.02–1.00%), 0.03% (95% CI 0.00–11.80%), and 0.00% (95% CI 0.00–60.24%) from women who were HBeAg-negative with low viremia, respectively.

## Discussion

In this systematic review and meta-analysis of 131 studies involving 71,712 women who were HBsAg-positive, the overall proportion of pregnant women with HBV infection eligible for PAP based on high HBV DNA levels and positive HBeAg was 21.27% (95% CI 17.77–25.26%) and 23.86% (95% CI 21.00–26.97%), respectively. Notably, these proportions exhibited significant regional disparities, with the WPR showing the highest pooled proportions of both high HBV DNA levels (31.56%) and positive HBeAg (34.53%). Furthermore, we examined the distribution of HBV-infected pregnant women cross-stratified by HBV DNA levels and HBeAg status. We found that over three-quarters of women (75.59%, 95% CI 69.15–81.05%) fell into the category of low viremia and negative HBeAg, with a risk of MTCT of 0.14% (95% CI 0.02–1.00%) using ‘HepB-BD only’, 0.03% (95% CI 0.00–11.80%) using ‘HepB-BD + HBIG’, and 0.00% (95% CI 0.00–60.24%) using ‘HepB-BD + HBIG + PAP’.

To date, only a limited number of systematic reviews have attempted to estimate the country- or region-specific proportions of positive HBeAg in HBV-infected pregnant women.[28], [29], [30], [31], [32], [33] Our results align with these previous studies, demonstrating a higher proportion of HBeAg positivity in Asia (>20%) than in the rest of the world (<20%). In the Americas, the pooled prevalence of HBeAg was relatively high (23.05%), probably because of the overrepresentation of women of Asian origin.^17^^,^^18^^,^^20^ After excluding these studies, the pooled proportion was 19.72% (95% CI 18.13–21.42%). The substantial geographical variation in HBeAg prevalence can be best elucidated by a feedback mechanism that links the mode of transmission with the natural history of chronic HBV infection.^34^ MTCT is more likely than horizontal transmission to result in chronic HBV infection.^34^ In addition, among individuals who have established chronic HBV infection, MTCT further increases the risk of prolonged periods of HBeAg persistence and high HBV replication beyond the reproductive age,^35^^,^^36^ thereby perpetuating the cycle of MTCT in subsequent generations. Another contributing factor to this geographical variation is the difference in circulating HBV genotypes.^35^^,^^36^ Importantly, our study has confirmed a similar pattern of geographical variation in the proportion of pregnant women with high HBV DNA levels, providing further support for the aforementioned hypothesis.

Maternal HBV DNA levels during pregnancy have been established as the most reliable marker for predicting MTCT.^37^ However, in resource-limited countries where access to HBV DNA testing is limited, the WHO conditionally recommended the use of HBeAg as an alternative indicator to determine eligibility for PAP.^5^ Although positive HBeAg is closely correlated with elevated HBV DNA levels in HBV-infected pregnant women,^6^ a subset of women may still carry high viral loads despite having negative HBeAg. This can occur because of the emergence of viral mutations that reduce HBeAg production while maintaining viral replication capacity.^38^ To evaluate the independent role of HBV DNA levels and HBeAg status in relation to MTCT risk, we examined the proportion of women with high HBV DNA levels but negative HBeAg and assessed their risk of MTCT. Among the four groups cross-stratified by these two markers, the subgroup identified as ‘high viral loads and negative HBeAg’ represented the smallest proportion (2.70%, 95% CI 1.88–3.86%). Moreover, this specific subgroup, characterized by ‘high viral loads and negative HBeAg’, appeared to have a lower risk of MTCT than the group with ‘high viral loads and positive HBeAg. Specifically, the rates of MTCT were 6.94 *vs*. 14.86% after HepB-BD only, 2.50 *vs*. 5.50% after HepB-BD + HBIG, and 0.00 *vs*. 1.32% after HepB-BD + HBIG + PAP, respectively. It is important to emphasize that these estimates were derived from a limited number of studies with a small sample size. Despite this limitation, the findings suggest that the potential impact of overlooking women who were HBeAg-negative with high HBV DNA levels, as a result of HBeAg-guided risk stratification, may have a relatively limited contribution compared with the oversight of women who were HBeAg-positive with similar virological profiles.

To effectively eliminate the MTCT of HBV, it is crucial to expand PMTCT interventions by integrating HBV screening, risk stratification, and administration of PAP into routine antenatal care.^5^ In addition, integrating HBV PMTCT efforts with other infectious disease control programs targeting HIV or syphilis may further enhance the overall effectiveness.^7^ However, in resource-limited countries, the available options for risk stratification following positive HBsAg screening should be carefully considered. The conventional HBV DNA-guided strategy is expected to be highly reliable, but limited access to this test in such contexts may raise concerns about its feasibility. Similarly, the alternative strategy guided by HBeAg detection, using laboratory-based immunoassays, faces challenges in accessibility, especially in decentralized resource-limited settings. The use of a rapid diagnostic test to detect HBeAg, although having limitations in analytical sensitivity, could potentially be improved by coupling it with alanine transaminase levels, thereby enhancing its diagnostic sensitivity.^39^ Moreover, emerging biomarkers, such as a rapid test for hepatitis B core-related antigen (HBcrAg), provide additional options for risk assessment.^40^ As an ultimate form of simplification, the universal administration of PAP to all women who were HBsAg-positive, without any risk stratification, has recently been proposed.^41^ However, its feasibility and acceptability are currently unknown. Given the regional heterogeneity in the proportion of women eligible for PAP, the varying availability of different tests, and their associated costs across different countries, the findings of our study can provide valuable insights to help each country define the most optimal strategy for effectively eliminating HBV MTCT.

Our study has limitations. First, owing to the limited number of studies evaluating viral genotypes or coinfection with HCV or HDV, we were unable to perform subgroup analyses based on these factors. Methodological heterogeneity was also identified as a potential limitation, particularly regarding the comprehensive description of the HBsAg screening process. The spectrum and prevalence of both high HBV DNA levels and HBeAg may differ between studies that exclusively recruited women newly identified as positive HBsAg and those that included women known to have chronic HBV infection. We observed that studies with a more detailed description of HBsAg screening tended to provide lower estimates, suggesting that the pooled estimates in our study may be slightly overestimated compared with the general population of pregnant women. We conducted a systematic review of observational studies without any language restriction. However, the inclusion of Chinese-language articles posed challenges, as patient groups reported in these articles often overlapped with those in English-language articles, making it challenging to ascertain their uniqueness.^9^ As a result, we opted not to include Chinese-language articles in our systematic review. Of note, this decision is unlikely to impact our work, given the substantial number of included studies conducted in China (n = 48). Lastly, we encountered a limited number of studies for the Q4, which examined the risk of MTCT based on maternal groups stratified by HBV DNA levels and HBeAg status. The scarcity of these studies was compounded by the need for additional stratification considering the type of maternal antiviral prophylaxis and infant immunoprophylaxis. We therefore could not examine the regional difference in the risk of MTCT. The limited availability of data highlights the need for additional research to further elucidate the potential role of HBeAg in modifying the risk of MTCT, independently of HBV DNA levels.^37^

In conclusion, our study found that approximately one-fifth of pregnant women with HBV-infection are eligible for PAP. There is a significant variation in this proportion between the WPR and the rest of the world. These findings underscore the crucial need to optimize strategies for expanding PMTCT interventions, considering the regional differences in the proportion of high-risk women. By integrating these interventions and tailoring them to local contexts, countries can make substantial progress toward the goal of MTCT elimination.

## Financial support

There was no funding source for this study.

## Authors’ contributions

Conceived the study: YS. Developed the study protocol: MC, YS. Screened the titles and abstracts: MC, AR. Extracted the data: HD, JEIR, AR. Performed the statistical analysis: HD and JEIR. Contributed to supervision: JPV, YS. Provided technical support to complete the study: OS, SN. Wrote the first draft of the manuscript: HD, JEIR, YS. Had full access to all the data in the study, read and approved the final version of the manuscript, and had final responsibility for the decision to submit for publication: all authors.

## Data availability statement

The full search strategy and key results used to generate data that inform the conclusion of this systematic review can be found in the Supplementary information.

## Conflicts of interest

YS has received a research grant from Gilead and research materials from Abbott Laboratories and Fujirebio Inc.

Please refer to the accompanying ICMJE disclosure forms for further details.

## References

[1] GBD 2019 Hepatitis B Collaborators (2022). Global, regional, and national burden of hepatitis B, 1990–2019: a systematic analysis for the Global Burden of Disease Study 2019. Lancet Gastroenterol Hepatol.

[2] Polaris Observatory Collaborators (2018). Global prevalence, treatment, and prevention of hepatitis B virus infection in 2016: a modelling study. Lancet Gastroenterol Hepatol.

[3] World Health Organization (2021). https://apps.who.int/iris/handle/10665/342813.

[4] Shimakawa Y., Yan H.-J., Tsuchiya N. (2013). Association of early age at establishment of chronic hepatitis B infection with persistent viral replication, liver cirrhosis and hepatocellular carcinoma: a systematic review. PLoS One.

[5] Prevention of mother-to-child transmission of hepatitis B virus: guidelines on antiviral prophylaxis in pregnancy. https://www.who.int/publications-detail-redirect/978-92-4-000270-8 Accessed 5 April 2023. 32833415

[6] Boucheron P., Lu Y., Yoshida K. (2021). Accuracy of HBeAg to identify pregnant women at risk of transmitting hepatitis B virus to their neonates: a systematic review and meta-analysis. Lancet Infect Dis.

[7] Cohn J., Owiredu M.N., Taylor M.M. (2021). Eliminating mother-to-child transmission of human immunodeficiency virus, syphilis and hepatitis B in sub-Saharan Africa. Bull World Health Organ.

[8] Ansari A., Vincent J.P., Moorhouse L. (2023). Risk of early horizontal transmission of hepatitis B virus in children of uninfected mothers in sub-Saharan Africa: a systematic review and meta-analysis. Lancet Glob Health.

[9] Zhou Y.-H. (2016). Prevention of mother-to-child transmission of hepatitis B virus by treating mothers with high viral loads. Hepatology.

[10] Hoy D., Brooks P., Woolf A. (2012). Assessing risk of bias in prevalence studies: modification of an existing tool and evidence of interrater agreement. J Clin Epidemiol.

[11] Altman D.G. (2001).

[12] Hunter J.P., Saratzis A., Sutton A.J. (2014). In meta-analyses of proportion studies, funnel plots were found to be an inaccurate method of assessing publication bias. J Clin Epidemiol.

[13] Kfutwah A.K., Tejiokem M.C., Njouom R. (2012). A low proportion of HBeAg among HBsAg-positive pregnant women with known HIV status could suggest low perinatal transmission of HBV in Cameroon. Virol J.

[14] Rouet F., Chaix M.-L., Inwoley A. (2004). HBV and HCV prevalence and viraemia in HIV-positive and HIV-negative pregnant women in Abidjan, Côte d’Ivoire: the ANRS 1236 study. J Med Virol.

[15] Thumbiran N.V., Moodley D., Parboosing R. (2014). Hepatitis B and HIV co-infection in pregnant women: indication for routine antenatal hepatitis B virus screening in a high HIV prevalence setting. S Afr Med J.

[16] Keel P., Edwards G., Flood J. (2016). Assessing the impact of a nurse-delivered home dried blood spot service on uptake of testing for household contacts of hepatitis B-infected pregnant women across two London trusts. Epidemiol Infect.

[17] Lyu J., Wang S., He Q. (2020). Hep B moms: a cross-sectional study of mother-to-child transmission risk among pregnant Asian American women with chronic hepatitis B in New York City, 2007–2017. J Viral Hepat.

[18] Nguyen G., Garcia R.T., Nguyen N. (2009). Clinical course of hepatitis B virus infection during pregnancy. Aliment Pharmacol Ther.

[19] Bzowej N.H., Tran T.T., Li R. (2019). Total alanine aminotransferase (ALT) flares in pregnant North American women with chronic hepatitis B infection: results from a prospective observational study. Am J Gastroenterol.

[20] Patel N.H., Joshi S.S., Lau K.C.K. (2019). Analysis of serum hepatitis B virus RNA levels in a multiethnic cohort of pregnant chronic hepatitis B carriers. J Clin Virol.

[21] Fujiko M., Chalid M.T., Turyadi (2015). Chronic hepatitis B in pregnant women: is hepatitis B surface antigen quantification useful for viral load prediction?. Int J Infect Dis.

[22] Geffert K., Maponga T.G., Henerico S. (2020). Prevalence of chronic HBV infection in pregnant woman attending antenatal care in a tertiary hospital in Mwanza, Tanzania: a cross-sectional study. BMC Infect Dis.

[23] Kishk R., Mandour M., Elprince M. (2020). Pattern and interpretation of hepatitis B virus markers among pregnant women in North East Egypt. Braz J Microbiol.

[24] Schulpis K.H., Barzeliotou A., Papadakis M. (2008). Maternal chronic hepatitis B virus is implicated with low neonatal paraoxonase/arylesterase activities. Clin Biochem.

[25] Chakvetadze C., Roussin C., Roux J. (2011). Efficacy of hepatitis B sero-vaccination in newborns of African HBsAg positive mothers. Vaccine.

[26] Li Y., Wang J., Yu Y. (2020). Maternal antiviral treatment safeguards infants from hepatitis B transmission in contingencies of delayed immunoprophylaxis. Liver Int.

[27] Liu J., Chen T., Chen Y. (2020). 2019 Chinese clinical practice guidelines for the prevention of mother-to-child transmission of hepatitis B virus. J Clin Transl Hepatol.

[28] Giri S., Sahoo S., Angadi S. (2022). Seroprevalence of hepatitis B virus among pregnant women in India: a systematic review and meta-analysis. J Clin Exp Hepatol.

[29] Liu D., Liu Y., Ni J. (2022). Hepatitis B infection among pregnant women in China: a systematic review and meta-analysis. Front Public Health.

[30] Olakunde B.O., Adeyinka D.A., Olakunde O.A. (2021). A systematic review and meta-analysis of the prevalence of hepatitis B virus infection among pregnant women in Nigeria. PLoS One.

[31] Liu X., Chen C., Jiang D. (2021). Comparison of HBV-DNA and HBeAg as antiviral therapeutic indicators among HBV-infected pregnant women: a systematic review and meta-analysis. Ann Palliat Med.

[32] Bigna J.J., Kenne A.M., Hamroun A. (2019). Gender development and hepatitis B and C infections among pregnant women in Africa: a systematic review and meta-analysis. Infect Dis Poverty.

[33] Ott J.J., Stevens G.A., Wiersma S.T. (2012). The risk of perinatal hepatitis B virus transmission: hepatitis B e antigen (HBeAg) prevalence estimates for all world regions. BMC Infect Dis.

[34] Moutchia J., Njouom R., Rumpler E. (2022). Maternal age at first childbirth and geographical variation in hepatitis B virus prevalence in Cameroon: important role of mother-to-child transmission. Clin Infect Dis.

[35] Yang Y., Huang A., Zhao Y. (2021). Spontaneous loss of chronic HBV infection markers in treatment-naïve children: a systematic review and pooled meta-analyses. Expert Rev Anti Infect Ther.

[36] Mohareb A.M., Liu A.F., Kim A.Y. (2022). Clearance of hepatitis B e antigen in untreated chronic hepatitis B virus infection: a systematic review and meta-analysis. J Infect Dis.

[37] Pan C.Q., Duan Z.-P., Bhamidimarri K.R. (2012). An algorithm for risk assessment and intervention of mother to child transmission of hepatitis B virus. Clin Gastroenterol Hepatol.

[38] Kramvis A. (2016). The clinical implications of hepatitis B virus genotypes and HBeAg in pediatrics. Rev Med Virol.

[39] Segeral O., Dim B., Durier C. (2020). Hepatitis B e antigen (HBeAg) rapid test and alanine aminotransferase level–based algorithm to identify pregnant women at risk of HBV mother-to-child transmission: the ANRS 12345 TA PROHM study. Clin Infect Dis.

[40] Shimakawa Y., Ndow G., Kaneko A. (2023). Rapid point-of-care test for hepatitis B core-related antigen to diagnose high viral load in resource-limited settings. Clin Gastroenterol Hepatol.

[41] Nayagam S., de Villiers M.J., Shimakawa Y. (2023). Impact and cost-effectiveness of hepatitis B virus prophylaxis in pregnancy: a dynamic simulation modelling study. Lancet Gastroenterol Hepatol.

