# Supplementary material for: Proportion of pregnant women with HBV infection eligible for antiviral prophylaxis to prevent vertical transmission: A systematic review and meta-analysis
Source: JHEP Rep. 2024 Mar 26;6(8):101064. doi: 10.1016/j.jhepr.2024.101064 (PMC11260332; doi:10.1016/j.jhepr.2024.101064)
Supplement: Multimedia component 4 [file mmc4.pdf]

Proportion of pregnant women with HBV infection eligible for antiviral prophylaxis to prevent vertical transmission: A systematic review and meta-analysis

Hugues Delamare,[1,†] **Julian Euma Ishii-Rousseau**,[2,†] **Adya Rao**,[3,†] **Mélanie Cresta**,[1,†] Jeanne Perpétue Vincent,[1] Olivier Ségéral,[4] Shevanthi Nayagam,[3] Yusuke Shimakawa[1,*]

[1]Institut Pasteur, Université Paris Cité, Unité d'Épidémiologie des Maladies Émergentes, Paris, France; [2]Department of Global Health Promotion, Tokyo Medical and Dental University, Tokyo, Japan; [3]MRC Centre for Infectious Disease Analysis, School of Public Health, Imperial College London, UK; [4]Unité VIH/Sida, Service des maladies infectieuses, Hôpitaux Universitaires de Genève, Genève, Switzerland

JHEP Reports **2024.** https://doi.org/10.1016/j.jhepr.2024.101064

Background & Aims: In 2020, the World Health Organization (WHO) recommended peripartum antiviral prophylaxis (PAP) for pregnant women infected with hepatitis B virus (HBV) with high viremia (≥200,000 IU/ml). Hepatitis B e antigen (HBeAg) was also recommended as an alternative when HBV DNA is unavailable. To inform policymaking and guide the implementation of prevention of mother-to-child transmission strategies, we conducted a systematic review and meta-analysis to estimate the proportion of HBV-infected pregnant women eligible for PAP at global and regional levels.
Methods: We searched PubMed, EMBASE, Scopus, and CENTRAL for studies involving HBV-infected pregnant women. We extracted proportions of women with high viremia (≥200,000 IU/ml), proportions of women with positive HBeAg, proportions of women cross-stratified based on HBV DNA and HBeAg, and the risk of child infection in these maternal groups. Proportions were pooled using random-effects meta-analysis.
Results: Of 6,999 articles, 131 studies involving 71,712 HBV-infected pregnant women were included. The number of studies per WHO region was 66 (Western Pacific), 21 (Europe), 17 (Africa), 11 (Americas), nine (Eastern Mediterranean), and seven (South-East Asia). The overall pooled proportion of high viremia was 21.27% (95% CI 17.77–25.26%), with significant regional variation: Western Pacific (31.56%), Americas (23.06%), Southeast Asia (15.62%), Africa (12.45%), Europe (9.98%), and Eastern Mediterranean (7.81%). HBeAg positivity showed similar regional variation. After cross-stratification, the proportions of high viremia and positive HBeAg, high viremia and negative HBeAg, low viremia and positive HBeAg, and low viremia and negative HBeAg were 15.24% (95% CI 11.12–20.53%), 2.70% (95% CI 1.88–3.86%), 3.69% (95% CI 2.86–4.75%), and 75.59% (95% CI 69.15–81.05%), respectively. The corresponding risks of child infection following birth dose vaccination without immune globulin and PAP were 14.86% (95% CI 8.43–24.88%), 6.94% (95% CI 2.92–15.62%), 7.14% (95% CI 1.00–37.03%), and 0.14% (95% CI 0.02–1.00%).
Conclusions: Approximately 20% of HBV-infected pregnant women are eligible for PAP. Given significant regional variations, each country should tailor strategies for HBsAg screening, risk stratification, and PAP in routine antenatal care.
Impact and implications: In 2020, the WHO recommended that pregnant women who test positive for the hepatitis B surface antigen (HBsAg) undergo HBV DNA testing or HBeAg and those with high viremia (≥200,000 IU/ml) or positive HBeAg receive PAP. To effectively implement new HBV PMTCT interventions and integrate HBV screening, risk stratification, and antiviral prophylaxis into routine antenatal care services, estimating the proportion of HBV-infected pregnant women eligible for PAP is critical. In this systematic review and meta-analysis, we found that approximately one-fifth of HBV-infected pregnant women are eligible for PAP based on HBV DNA testing, and a similar proportion is eligible based on HBeAg testing. Owing to substantial regional variations in eligibility proportions and the availability and costs of different tests, it is vital for each country to optimize strategies that integrate HBV screening, risk stratification, and PAP into routine antenatal care services.
Systematic review registration: This study was registered with PROSPERO (Protocol No: CRD42021266545).
© 2024 The Author(s). Published by Elsevier B.V. on behalf of European Association for the Study of the Liver (EASL). This is an open access article under the CC BY license (http://creativecommons.org/licenses/by/4.0/).

Introduction

Globally, 316 million individuals are living with chronic hepatitis B virus (HBV) infection,[1] of whom 95% reside in low- and middle-income countries (LMICs).[2] An estimated 820,000 annual deaths are attributed to HBV-related cirrhosis or hepatocellular carcinoma.[3] The World Health Organization (WHO) has set a goal to globally eliminate HBV infection as a public health threat by 2030, including achieving a 0.1% prevalence of HBsAg in children

Keywords: Hepatitis B; Elimination; Mother-to-child transmission; Pregnant women; HBV DNA; HBeAg; Systematic review.
Received 25 October 2023; received in revised form 4 March 2024; accepted 8 March 2024; available online 26 March 2024
† These authors contributed equally to this work.
* Corresponding author. Address: Institut Pasteur, Université Paris Cité, Unité d'Épidémiologie des Maladies Émergentes, 25-28 rue du Dr Roux, 75015 Paris, France. Tel.: +33-1-40-61-39-58; Fax: +33-1-45-68-88-76.
E-mail address: yusuke.shimakawa@gmail.com (Y. Shimakawa).

ELSEVIER
EASL The Home of Hepatology

aged 5 years.[3] Preventing mother-to-child transmission (MTCT) of HBV is crucial in reaching this goal, as this mode of transmission is a major risk factor for chronic HBV infection and related liver diseases, compared with horizontal transmission later in life.[4]

The WHO has recommended since 2009 that all infants should receive a series of three to four doses of hepatitis B vaccine starting immediately after birth, preferably within 24 h, to prevent perinatal MTCT and early horizontal transmission.[5] Moreover, to further reduce the MTCT risk, the WHO published in 2020 its first guidelines for peripartum antiviral prophylaxis (PAP), recommending that pregnant women who test positive for HBsAg and have HBV DNA levels of 200,000 IU/ml or greater should receive tenofovir prophylaxis from at least the 28th week of pregnancy until birth.[5] The use of HBeAg testing, as an alternative means of determining eligibility for tenofovir prophylaxis, was also recommended for pregnant women with limited access to quantitative HBV DNA testing.[5,6]

To effectively implement prevention of MTCT (PMTCT) interventions and integrate HBV screening, risk stratification, and antiviral prophylaxis into routine antenatal care services, it is essential to estimate the proportion of HBV-infected pregnant women who are eligible for peripartum antiviral prophylaxis.[5,7] In LMICs, where most HBV infections occur, understanding regional differences in this proportion can help optimize resource allocation for this critical intervention. To address these issues, we conducted a systematic review and meta-analysis to estimate the following: (Q1) the proportion of HBV-infected pregnant women with high HBV DNA levels (≥200,000 IU/ml), (Q2) the proportion of HBV-infected pregnant women who test positive for HBeAg, (Q3) the proportion of HBV-infected pregnant women classified into four subgroups based on their HBeAg serostatus and HBV DNA levels (HBeAg-positive with high viremia, HBeAg-positive with low viremia, HBeAg-negative with high viremia, and HBeAg-negative with low viremia), and (Q4) the risk of child infection in each of the four maternal subgroups.

Materials and methods

Search strategy and selection criteria

We searched PubMed, EMBASE, Scopus, and CENTRAL for studies published between January 1, 2000, and June 22, 2021, without any language restrictions. The search strategy used the terms 'hepatitis B infection' AND ('viral load' OR 'HBeAg') AND 'pregnancy' and their variations (Supplementary Methods 1). References for included studies were used to manually identify additional studies.

We included studies evaluating HBV DNA levels and/or HBeAg serostatus, anytime during pregnancy, in HBsAg-positive pregnant women who did not receive any anti-HBV therapy at the baseline assessment. We accepted studies providing antiviral therapy to these women after the baseline assessment. We excluded studies that selected pregnant women based on their HBeAg status or viral load, as well as those with fewer than 10 participants for a given question.

Eligibility criteria were developed for each of the four questions. To address Q1, studies were required to report the proportion of pregnant women with HBV DNA levels equal to or greater than 200,000 IU/ml (≥5.3 log IU/ml). However, as not all studies used this threshold, we also accepted studies that dichotomized viral loads into high and low categories using a threshold ranging from 100,000 (5.0 log) IU/ml to 1,000,000 (6.0 log) IU/ml. For Q2, studies were included if the proportion of pregnant women with positive HBeAg was available. For Q3, studies reporting the number of women in a subgroup defined by both HBV DNA levels (high or low, at a threshold range of 100,000–1,000,000 IU/ml) and HBeAg serostatus (positive or negative) were included. For Q4, studies reporting the risk of child infection in each of the four maternal categories defined above were included. Child infection was defined based on HBsAg positivity in infants aged between 6 and 12 months.[5] However, for infants who received at least three doses of hepatitis B vaccine, the definition was expandable up to 24 months because of the negligible risk of horizontal transmission in these children.[8] Corresponding authors were contacted when critical information was missing.

Titles and abstracts of all articles identified through the literature search were independently screened by two reviewers (MC and AR). This was followed by a full-text review and data extraction using a pre-piloted sheet (Supplementary Methods 2) by two additional independent reviewers (HD and JEIR). Any discrepancies were resolved by YS. Extracted data included study setting, study design, recruitment period, maternal and infant HBV markers (type and timing of sampling, as well as assay type), number of participants, their demographics and characteristics, and administration of maternal antiviral prophylaxis or infant immunoprophylaxis. Viral loads reported as copies/ml were converted to IU/ml.[5] When we encountered articles reporting overlapping populations and settings, a main study was selected based on completeness and relevance to the eligibility criteria, whereas other overlapping studies were discounted. However, the inclusion of Chinese-language articles presented additional challenges. Patient groups reported in these articles often appeared in English-language articles as well, making it difficult to determine their uniqueness.[9] We therefore made the decision not to consider Chinese-language articles in our systematic review. The risk of bias was assessed using a tool developed by Hoy *et al.*[10] for the first three questions (Supplementary Methods 3) and the Altman[11] framework for the fourth question (Supplementary Methods 4). The protocol was preregistered in PROSPERO (CRD42021266545). The study was reported according to the PRISMA guidelines.

Data analysis

The meta-analysis was conducted using the 'metaprop' command with RStudio version 3.3.0+ (PBC, Boston, MA, USA). Proportions, specific to each WHO region, were pooled via a random-effects meta-analysis using a generalized linear mixed model with a logit link approach. The percentage of heterogeneity was evaluated using the I^2 statistic. To explore the sources of heterogeneity for Q1 and Q2, subgroup analyses were performed on median or mean maternal age, maternal coinfection with HIV, study design, recruitment site, median recruitment year, whether the HBsAg screening process was fully described or not, and the uptake rate for HBV DNA quantification or HBeAg testing. In addition, the viral load cut-off used in each study was assessed for Q1, and the type of HBeAg assay was assessed for Q2. For Q4, the risk of child infection was stratified by the administration of PAP and infant immunoprophylaxis (hepatitis B birth dose vaccine (HepB-BD) and/or hepatitis B immune globulin (HBIG)). Heterogeneity between subgroups was assessed using the meta-regression and test of moderators. Two-sided p <0.05 was considered as statistically significant. Small-study effects were visually assessed by plotting study size against the logarithm of the odds of proportion.[12]

Results

Of 6,999 articles identified, 1,311 underwent full-text assessment. Finally, 131 distinct studies reported in 172 articles met the inclusion criteria and provided data for 71,712 women who were HBsAg-positive (Fig. 1 and Supplementary References 1 and Supplementary Results 1). Notably, four studies reported two distinct groups each – either because some women had HIV co-infection (three studies)[13–15] or they were monitored differently (one study).[16] This gave us a total of 135 cohorts for meta-analysis. The numbers of cohorts and studies evaluated in each of the questions were as follows: 67 cohorts from 67 studies for Q1, 129 cohorts from 125 studies for Q2, 40 cohorts from 40 studies for Q3, and 11 cohorts from 11 studies for Q4 (Supplementary References 1). Study characteristics are presented in Supplementary Results 2. The majority of the studies were conducted in the WHO Western Pacific Region (WPR: n = 66, 50%), especially in China (n = 48, 36%), followed by the European Region (EUR: n = 21, 16%), African Region (AFR: n = 17, 13%), Regions of the Americas (AMR: n = 11, 8%), Eastern Mediterranean Region (EMR: n = 9, 7%), and South-East Asia Region (SEAR: n = 7, 5%). Study designs were prospective (n = 65, 48%), retrospective (n = 38, 29%), or cross-sectional (n = 28, 22%). Apart from 11 studies that did not specify the type of assays used to quantify HBV DNA levels, all the studies used PCR assays. HBeAg was detected using enzyme immunoassay (n = 58, 45%), chemiluminescent immunoassay (n = 32, 24%), fluorescent immunoassay (n = 4, 3%), or a rapid diagnostic test (n = 5, 4%). In 30 studies (23%), the method of HBeAg detection was not reported.

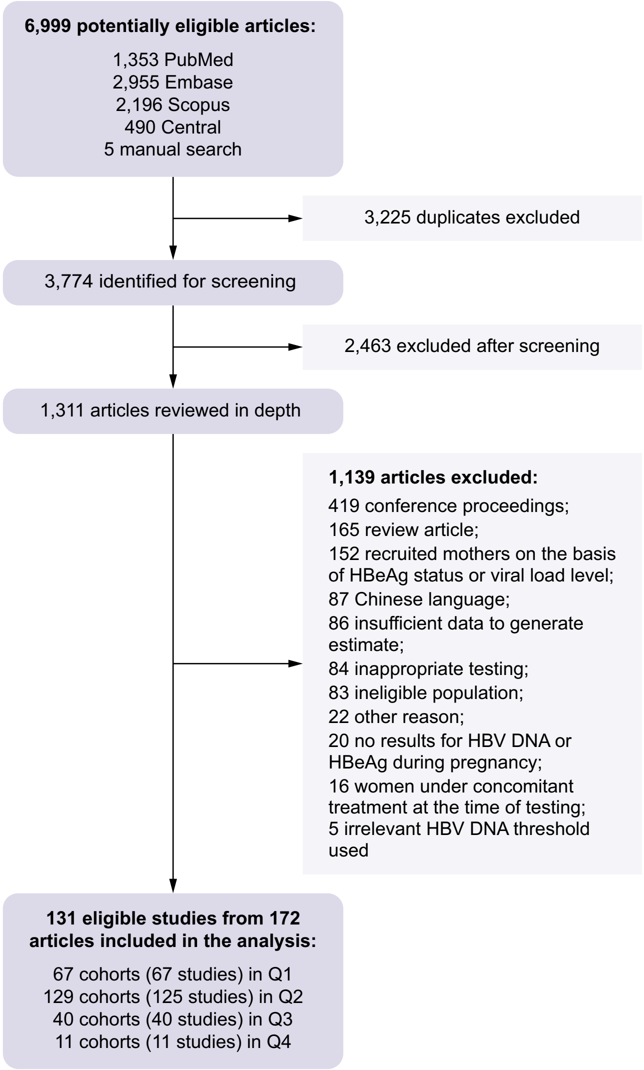

Fig. 1. Flow diagram of study selection.

The assessment of risk of bias is summarized in Supplementary Results 3. Regarding the representativeness of the study participants, the majority of the studies recruited women from tertiary centers (n = 94, 72%), whereas a smaller proportion recruited from primary care settings (n = 34, 26%). Sixty-two studies (47%) provided a comprehensive description of the HBsAg screening process, indicating a reduced susceptibility to selection bias. With respect to the uptake of HBV DNA quantification in women who were HBsAg-positive, 25 studies (36%) reported an uptake of ≥75%, 24 studies (36%) reported an uptake of <75%, and 18 studies (27%) did not provide the uptake information. The uptake was relatively higher for HBeAg testing: 57 studies (46%) reported an uptake of ≥75%, 27 studies (22%) reported an uptake of <75%, and 41 studies (33%) did not report the uptake information. The adapted funnel plots did not show any clear asymmetry (Supplementary Results 4), suggesting the lack of small-study effects.

Fig. 2 presents the proportion of women with high HBV DNA levels among 23,881 pregnant women with chronic HBV infection, derived from 67 cohorts (Q1). The overall pooled estimate was 21.27% (95% CI 17.77–25.26%), accompanied by considerable heterogeneity across the studies (I^2 = 96%). Significant variation was observed according to the WHO regions (test of moderators, p <0.0001), with the WPR having the highest proportion at 31.56% (95% CI 27.14–36.35%, I^2 = 95%). The AMR accounted for 23.06% (95% CI 17.30–30.04%, I^2 = 83%), making it the second-highest region, likely because of the inclusion of studies focusing on Asian communities in North America.[17–20] After excluding these four studies, the pooled estimate in the AMR was 17.51% (95% CI 16.07–19.04%, I^2 = 6%). For the rest of the world, the proportion was comparatively low when considered in relation to the WPR: AFR (12.45%, 95% CI 5.81–24.68%, I^2 = 95%), EUR (9.98%, 95% CI 8.00–12.39%, I^2 = 27%), and EMR (7.81%, 95% CI 2.64–20.94%, I^2 = 0%). Only one study from the SEAR was available for this analysis (15.62%, 95% CI 7.76–26.86%).[21] Outliers were identified, and their potential reasons are presented in Supplementary Results 5. Of four studies showing extremely lower estimates (<5%), three included a small number of participants (n <50).[22–24] All of five studies showing extremely higher estimates (>50% for the WPR and >30% for the rest of the world) recruited participants at specialized tertiary care centers.[18,19,25–27]

Owing to the large number of included studies in the WPR and the higher proportion of women with high HBV DNA levels in this region, subgroup analyses were conducted after stratifying the data into the WPR and the rest of the world (Table S1). The use of a higher threshold for HBV DNA levels was associated with a lower proportion of women surpassing these thresholds in the other regions (test of moderators, p = 0.0217), whereas such an association was not observed in the WPR. Studies providing a comprehensive description of the HBsAg screening process tended to yield lower estimates in both the WPR and the other regions (test of moderators, p <0.0001 and p = 0.0675, respectively).

Fig. 3 presents the proportion who test positive for HBeAg in 68,662 HBV-infected pregnant women from 129 cohorts (Q2).

	Threshold IU/ml (log_{10})	Number with HVL/total (n/N)	Prop (%)	95% CI
**WPR**				
Li F, 2012, China	5.30	12/221	5.43	[2.84; 9.29]
Khue P, 2020, Vietnam	5.30	24/183	13.11	[8.59; 18.88]
Zhang Z, 2014, China	5.30	35/174	20.11	[14.43; 26.85]
Lee L, 2015, Singapore	5.30	33/154	21.43	[15.23; 28.76]
Chen T, 2018, China	6.00	210/951	22.08	[19.48; 24.86]
Evans A, 2015, China	5.30	41/185	22.16	[16.40; 28.84]
Hui P, 2020, Hong Kong	5.30	60/265	22.64	[17.75; 28.16]
Cheung K, 2018, Hong Kong	5.30	167/641	26.05	[22.69; 29.63]
Ségéral O, 2018, Cambodia	5.30	34/128	26.56	[19.15; 35.09]
Li Z, 2014, China	5.30	151/537	28.12	[24.35; 32.13]
Hu Y, 2016, China	5.30	75/264	28.41	[23.05; 34.26]
Michitaka K, 2012, Japan	5.30	6/21	28.57	[11.28; 52.18]
Chen Y, 2013, China	5.30	49/171	28.65	[22.01; 36.06]
Thilakanathan C, 2018, Australia	5.00	185/642	28.82	[25.34; 32.49]
Bergin H, 2017, Australia	5.30	30/99	30.30	[21.47; 40.35]
Peng T, 2019, China	5.30	229/750	30.53	[27.25; 33.97]
Ségéral O, 2022, Cambodia	5.30	367/1,194	30.74	[28.13; 33.44]
Xu Y, 2015, China	5.30	96/312	30.77	[25.69; 36.22]
Wang L, 2016, China	5.30	10/31	32.26	[16.68; 51.37]
Guo Z, 2013, China	5.30	341/1,046	32.60	[29.76; 35.53]
Lu Y, 2017, China	5.00	388/1,177	32.97	[30.28; 35.73]
Xu C, 2018, China	5.30	74/214	34.58	[28.23; 41.37]
Lu L, 2014, China	5.00	50/140	35.71	[27.80; 44.25]
Kang W, 2014, China	5.00	1,031/2,765	37.29	[35.48; 39.12]
Nishimura K, 2021, Japan	5.30	33/87	37.93	[27.74; 48.97]
Zhang L, 2014, China	5.30	243/630	38.57	[34.75; 42.50]
Chen Z, 2017, China	5.30	139/338	41.12	[35.83; 46.58]
Latthaphasavang V, 2019, Laos	5.30	65/153	42.48	[34.54; 50.72]
Pan C, 2013, China	5.30	667/1,401	47.61	[44.97; 50.26]
Sasagawa Y, 2019, Japan	5.30	17/35	48.57	[31.38; 66.01]
Li L, 2020, China	5.00	156/317	49.21	[43.58; 54.86]
Liu Z, 2019, China	5.70	452/904	50.00	[46.69; 53.31]
Li Y, 2020, China	5.30	174/232	75.00	[68.91; 80.44]
**Pooled estimate**			**31.56**	**[27.14; 36.35]**
Heterogeneity: I^2 = 95%, τ^2 = 0.3620, p <0.01				
**AMR**				
Van Ommen C, 2019, Canada	5.30	6/59	10.17	[3.82; 20.83]
Izquierdo G, 2019, Chile	5.30	5/30	16.67	[5.64; 34.72]
Biondi M, 2020, Canada	5.30	273/1,556	17.54	[15.69; 19.53]
Kubo A, 2014, USA	5.30	147/835	17.60	[15.08; 20.36]
Lyu J, 2019, USA	5.30	278/1,241	22.40	[20.11; 24.83]
Tohme R, 2016, Haïti	5.30	9/33	27.27	[13.30; 45.52]
Patel N, 2019, Canada	5.30	12/41	29.27	[16.13; 45.54]
Bzowej N, 2019, USA	5.30	48/156	30.77	[23.64; 38.65]
Nguyen G, 2009, USA	5.30	15/27	55.56	[35.33; 74.52]
**Pooled estimate**			**23.06**	**[17.30; 30.04]**
Heterogeneity: I^2 = 83%, τ^2 = 0.2254, p <0.01				
**EUR**				
Schulpis K, 2008, Greece	5.30	0/28	0.00	[0.00; 12.34]
Bhattacharya S, 2008, United Kingdom	5.30	5/112	4.46	[1.47; 10.11]
Papaevangelou V, 2011, Greece	5.30	6/109	5.50	[2.05; 11.60]
Ruiz−Extremera Á, 2020, Spain	5.30	4/62	6.45	[1.79; 15.70]
Godbole G, 2013, United Kingdom	6.00	26/293	8.87	[5.88; 12.73]
Belopolskaya M, 2015, Russia	5.30	3/31	9.68	[2.04; 25.75]
White H, 2015, United Kingdom	5.30	13/123	10.57	[5.75; 17.40]
Sellier P, 2015, France	5.30	50/417	11.99	[9.03; 15.50]
Elefsiniotis I, 2007, Greece	5.30	8/63	12.70	[5.65; 23.50]
Safadi R, 2021, Israel	5.30	23/171	13.45	[8.72; 19.50]
Eilard A, 2019, Sweden	5.30	6/42	14.29	[5.43; 28.54]
Sellier P, 2018, France	5.30	3/17	17.65	[3.80; 43.43]
Dyson J, 2014, United Kingdom	5.30	12/67	17.91	[9.61; 29.20]
**Pooled estimate**			**9.98**	**[8.00; 12.39]**
Heterogeneity: I^2 = 27%, τ^2 = 0.0611, p = 0.17				

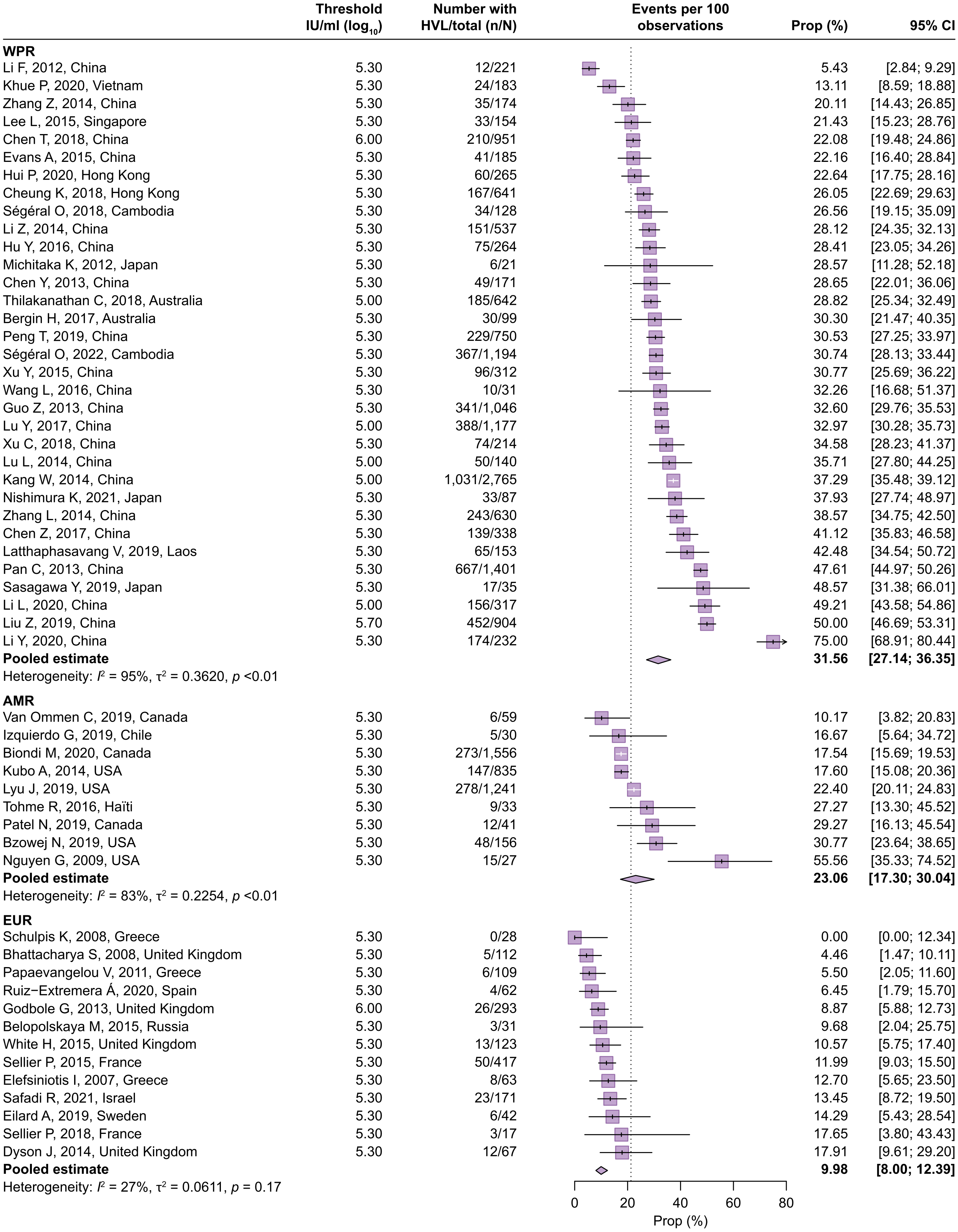

Fig. 2. Proportion of pregnant women with HBV infection with high HBV DNA levels. Proportions were pooled via a random-effects meta-analysis using a generalized linear mixed model with a logit link approach. AFR, African Region; AMR, Regions of the Americas; EMR, Eastern Mediterranean Region; EUR, European Region; SEAR, South-East Asia Region; WPR, Western Pacific Region.

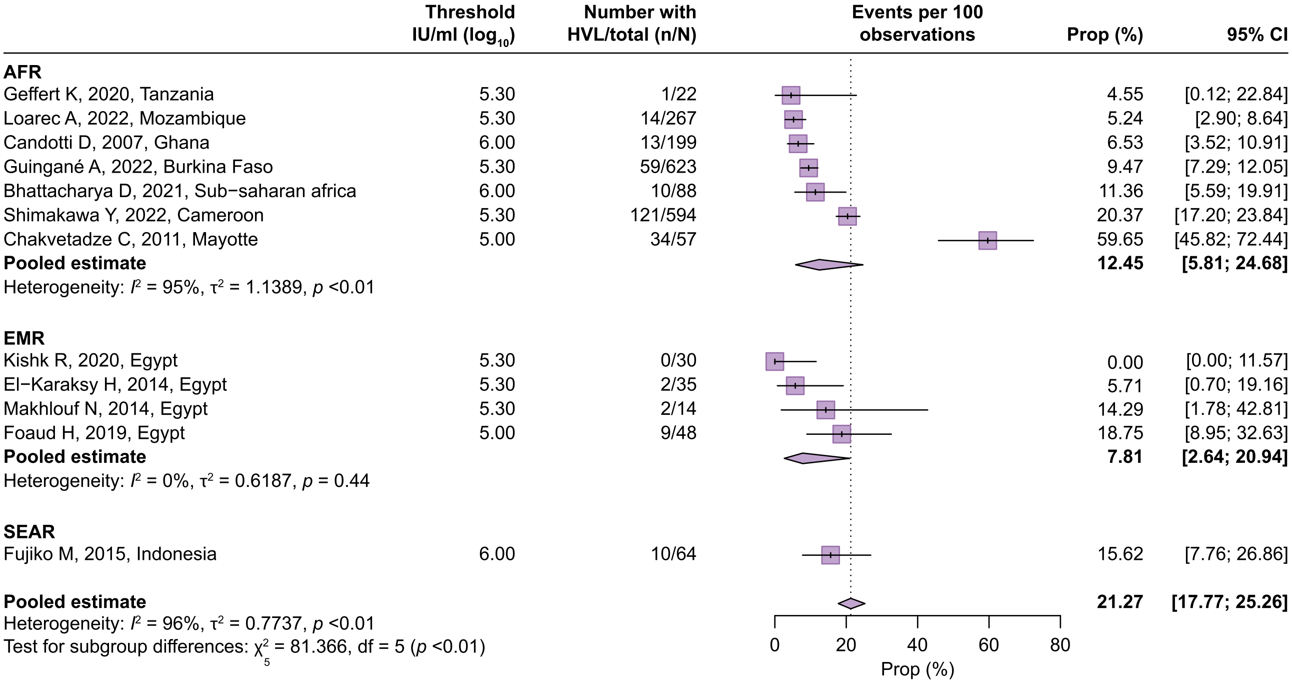

Fig. 2 (*continued*).

The overall pooled estimate was 23.86% (95% CI 21.00–26.97%). Similar to the proportion of women with high viremia, considerable heterogeneity was observed across the studies (I^2 = 96%) and across the WHO regions (test of moderators, p <0.0001). The proportion was the highest in the WPR (34.53%, 95% CI 31.34–37.86%), followed by the SEAR (28.23%, 95% CI 20.51–37.48%), the AMR (23.05%, 95% CI 18.75–28.00%), the AFR (15.09%, 95% CI 9.29–23.58%), the EUR (10.42%, 95% CI 7.39–14.50%), and the EMR (8.95%, 95% CI 3.88–19.31%). The characteristics of outlier cohorts are summarized in Supplementary Results 5. Subgroup analyses of the proportion of HBV-infected women who test positive for HBeAg are presented in Table S2. In the WPR, a lower age of women was significantly associated with a higher proportion of positive HBeAg (39.65%, 95% CI 31.97–47.87%) in studies with a median/mean age of <29 years compared with 29.38% (95% CI 25.78–33.26%) in studies with ≥29 years (p = 0.0154). This association was not confirmed in other WHO regions. Of the potential sources of methodological heterogeneity, there was strong evidence for the study design in other regions (p = 0.0264) and whether HBsAg screening process fully described or not in the WPR (p = 0.0397).

Fig. 4 presents the distribution of 10,386 HBV-infected pregnant women, from 39 cohorts, into the four subgroups defined by both HBV DNA levels and HBeAg serostatus (Q3). The overall pooled estimates of the proportion with high viremia and positive HBeAg, high viremia and negative HBeAg, low viremia and positive HBeAg, and low viremia and negative HBeAg were 15.24% (95% CI 11.12–20.53%, I^2 = 95%), 2.70% (95% CI 1.88–3.86%, I^2 = 83%), 3.69% (95% CI 2.86–4.75%, I^2 = 86%), and 75.59% (95% 69.15–81.05%, I^2 = 96%), respectively. In the WPR, the proportion with high viremia and positive HBeAg was relatively high, whereas in the other regions, the vast majority were in a subgroup of low viremia and negative HBeAg (Fig. S1). Only the minority represented the high viremia and HBeAg negative group and the low viremia and HBeAg positive group across the regions.

Fig. 5 presents the risk of child infection stratified by both maternal HBV markers and maternal and child prophylaxis administered (Q4). The majority of the cohorts provided one of the following combinations of the maternal/infant immunoprophylaxis: 'HepB-BD only', 'HepB-BD + HBIG', and 'HepB-BD + HBIG + PAP'. The risk of child infection following 'HepB-BD only', 'HepB-BD + HBIG', and 'HepB-BD + HBIG + PAP' was 14.86% (95% CI 8.43–24.88%), 5.50% (95% CI 2.49–11.71%), and 1.32% (95% CI 0.28–5.93%) from women who were HBeAg-positive with high viremia, respectively; 6.94% (95% CI 2.92–15.62%), 2.50% (95% CI 0.35–15.73%), and 0.00% (95% CI 0.00–100.00%) from women who were HBeAg-negative with high viremia, respectively; 7.14% (95% CI 1.00–37.03%), 0.00% (95% CI 0.00–100.00%), and 0.00% (95% CI 0.00–97.50%) from women who were HBeAg-positive with low viremia, respectively; and 0.14% (95% CI 0.02–1.00%), 0.03% (95% CI 0.00–11.80%), and 0.00% (95% CI 0.00–60.24%) from women who were HBeAg-negative with low viremia, respectively.

Discussion

In this systematic review and meta-analysis of 131 studies involving 71,712 women who were HBsAg-positive, the overall proportion of pregnant women with HBV infection eligible for PAP based on high HBV DNA levels and positive HBeAg was 21.27% (95% CI 17.77–25.26%) and 23.86% (95% CI 21.00–26.97%), respectively. Notably, these proportions exhibited significant regional disparities, with the WPR showing the highest pooled proportions of both high HBV DNA levels (31.56%) and positive HBeAg (34.53%). Furthermore, we examined the distribution of HBV-infected pregnant women cross-stratified by HBV DNA levels and HBeAg status. We found that over three-quarters of women (75.59%, 95% CI 69.15–81.05%) fell into the category of

Author	Number with HBeAg positive status/total (n/N)	Events per 100 observations	Prop (%)	95% CI
**WPR**				
Su H, 2011, China	79/457		17.29	[13.93; 21.07]
Peng S, 2018, China	249/1,345		18.51	[16.47; 20.69]
Michitaka K, 2012, Japan	4/21		19.05	[5.45; 41.91]
Ségéral O, 2022, Cambodia	253/1,194		21.19	[18.90; 23.62]
Shao Z, 2011, China	47/212		22.17	[16.77; 28.37]
Ségéral O, 2018, Cambodia	29/128		22.66	[15.73; 30.89]
Khue P, 2020, Vietnam	42/183		22.95	[17.07; 29.73]
Wu K, 2020, China	267/1,129		23.65	[21.20; 26.24]
Giles M, 2015, Australia	30/126		23.81	[16.68; 32.21]
Lv N, 2014, China	10/42		23.81	[12.05; 39.45]
Zhang Z, 2014, China	42/174		24.14	[17.98; 31.19]
Lin X, 2014, China	71/294		24.15	[19.37; 29.46]
Cheung K, 2018, Hong Kong	155/641		24.18	[20.91; 27.69]
Li F, 2012, China	54/221		24.43	[18.92; 30.65]
Chen HL, 2012, Taiwan	583/2,356		24.75	[23.01; 26.54]
Chen X, 2013, China	135/544		24.82	[21.24; 28.67]
Xu Y, 2015, China	78/312		25.00	[20.29; 30.19]
Lee L, 2015, Singapore	42/161		26.09	[19.49; 33.58]
Evans A, 2015, China	49/185		26.49	[20.28; 33.46]
Bergin H, 2017, Australia	24/87		27.59	[18.54; 38.21]
Liu J, 2017, China	5,848/20,827		28.08	[27.47; 28.69]
Lao T, 2015, Hong Kong	67/235		28.51	[22.83; 34.74]
Hu Y, 2016, China	76/264		28.79	[23.40; 34.66]
Wang L, 2016, China	9/31		29.03	[14.22; 48.04]
Wiseman E, 2009, Australia	91/313		29.07	[24.10; 34.45]
Qiao Y, 2019, China	1,221/4,112		29.69	[28.30; 31.12]
Nishimura K, 2021, Japan	61/205		29.76	[23.59; 36.52]
Thilakanathan C, 2018, Australia	192/642		29.91	[26.39; 33.61]
Li Z, 2014, China	161/537		29.98	[26.13; 34.05]
Chen T, 2018, China	290/951		30.49	[27.58; 33.53]
Yonghao G, 2017, China	103/336		30.65	[25.77; 35.89]
Lu L, 2014, China	45/140		32.14	[24.51; 40.55]
Yi W, 2018, China	1,091/3,367		32.40	[30.82; 34.01]
Xu D, 2002, China	133/402		33.08	[28.50; 37.92]
Xu C, 2018, China	72/214		33.64	[27.35; 40.40]
Sheng Q, 2018, China	151/441		34.24	[29.82; 38.88]
Chen Y, 2013, China	59/171		34.50	[27.41; 42.14]
Yin Y, 2012, China	471/1,355		34.76	[32.22; 37.36]
Wang C, 2016, China	311/890		34.94	[31.81; 38.18]
Lu Y, 2017, China	419/1,177		35.60	[32.86; 38.41]
Zhu Y, 2010, China	92/252		36.51	[30.56; 42.78]
Zhang L, 2014, China	435/1186		36.68	[33.93; 39.49]
Kim J, 2014, South Korea	60/159		37.74	[30.18; 45.76]
Shi X, 2017, China	57/150		38.00	[30.21; 46.28]
Wang J, 2005, China	16/42		38.10	[23.57; 54.36]
Huang H, 2020, China	373/973		38.34	[35.27; 41.47]
Latthaphasavang V, 2019, Laos	60/153		39.22	[31.43; 47.43]
Guo Z, 2013, China	410/1,043		39.31	[36.33; 42.35]
Wang D, 2019, China	115/290		39.66	[33.98; 45.54]
Kang W, 2014, China	1,141/2,765		41.27	[39.42; 43.13]
Guo J, 2015, China	64/144		44.44	[36.17; 52.95]
Komatsu H, 2016, Japan	14/31		45.16	[27.32; 63.97]
Sasagawa Y, 2019, Japan	16/35		45.71	[28.83; 63.35]
Li L, 2020, China	147/317		46.37	[40.78; 52.03]
Li X, 2003, China	72/151		47.68	[39.50; 55.96]
Pan C, 2013, China	764/1,401		54.53	[51.88; 57.16]
Liu J, 2018, China	645/1,097		58.80	[55.82; 61.73]
Wang Z, 2003, China	33/54		61.11	[46.88; 74.08]
Chen J, 2014, China	205/334		61.38	[55.92; 66.63]
Liu Z, 2019, China	561/893		62.82	[59.56; 66.00]
Ding Y, 2013, China	167/249		67.07	[60.85; 72.87]
Liu C, 2015, China	172/256		67.19	[61.07; 72.91]
Li Y, 2020, China	180/232		77.59	[71.67; 82.78]
**Pooled estimate**			**34.53**	**[31.34; 37.86]**
Heterogeneity: $I^2 = 97\%$, $\tau^2 = 0.3169$, $p = 0$				

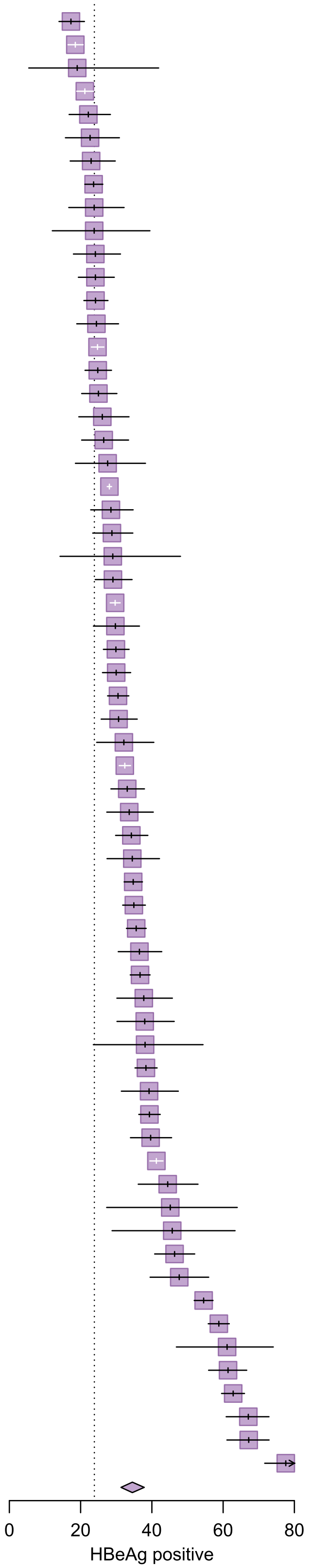

Fig. 3. Proportion of pregnant women with HBV infection who tested positive for HBeAg. Proportions were pooled via a random-effects meta-analysis using a generalized linear mixed model with a logit link approach. AFR, African Region; AMR, Regions of the Americas; EMR, Eastern Mediterranean Region; EUR, European Region; SEAR, South-East Asia Region; WPR, Western Pacific Region.

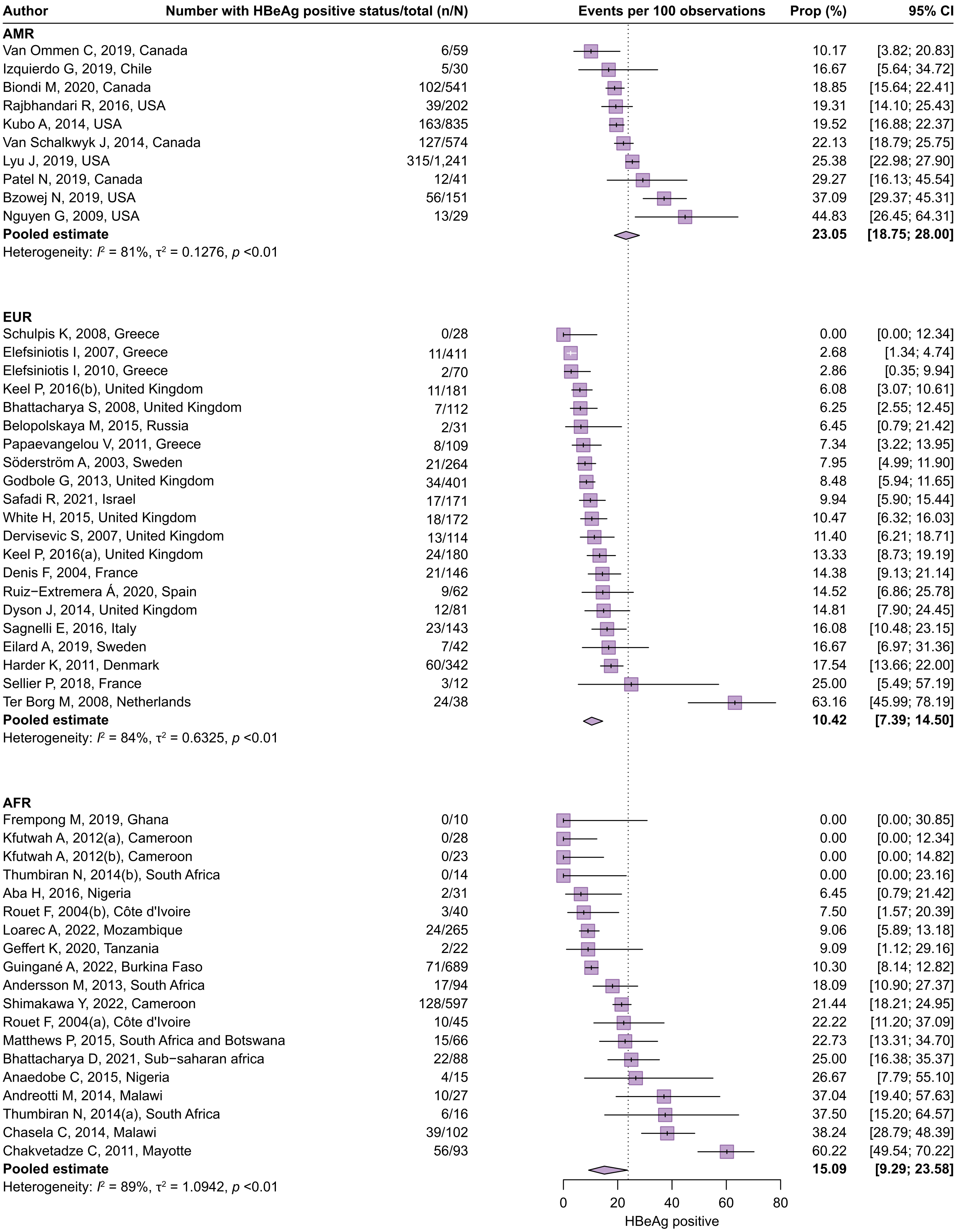

Fig. 3 (*continued*).

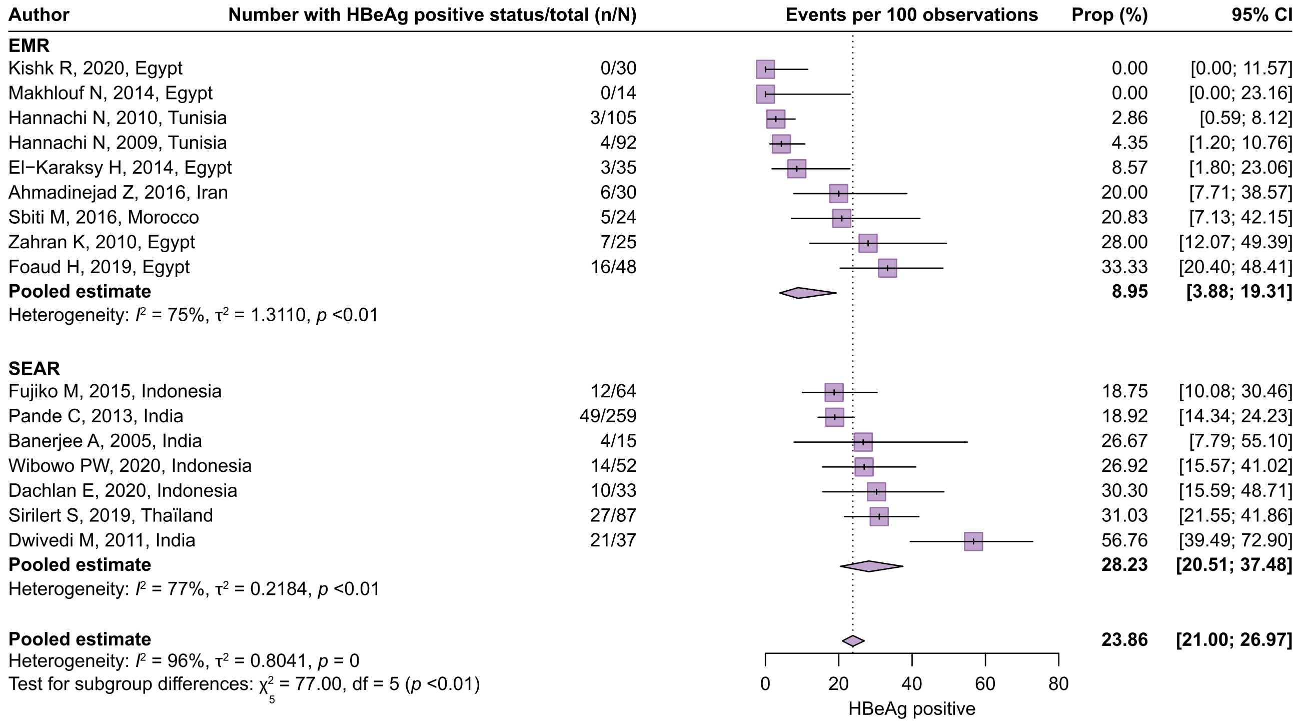

Fig. 3 (*continued*).

low viremia and negative HBeAg, with a risk of MTCT of 0.14% (95% CI 0.02–1.00%) using 'HepB-BD only', 0.03% (95% CI 0.00–11.80%) using 'HepB-BD + HBIG', and 0.00% (95% CI 0.00–60.24%) using 'HepB-BD + HBIG + PAP'.

To date, only a limited number of systematic reviews have attempted to estimate the country- or region-specific proportions of positive HBeAg in HBV-infected pregnant women.[28–33] Our results align with these previous studies, demonstrating a higher proportion of HBeAg positivity in Asia (>20%) than in the rest of the world (<20%). In the Americas, the pooled prevalence of HBeAg was relatively high (23.05%), probably because of the over-representation of women of Asian origin.[17,18,20] After excluding these studies, the pooled proportion was 19.72% (95% CI 18.13–21.42%). The substantial geographical variation in HBeAg prevalence can be best elucidated by a feedback mechanism that links the mode of transmission with the natural history of chronic HBV infection.[34] MTCT is more likely than horizontal transmission to result in chronic HBV infection.[34] In addition, among individuals who have established chronic HBV infection, MTCT further increases the risk of prolonged periods of HBeAg persistence and high HBV replication beyond the reproductive age,[35,36] thereby perpetuating the cycle of MTCT in subsequent generations. Another contributing factor to this geographical variation is the difference in circulating HBV genotypes.[35,36] Importantly, our study has confirmed a similar pattern of geographical variation in the proportion of pregnant women with high HBV DNA levels, providing further support for the aforementioned hypothesis.

Maternal HBV DNA levels during pregnancy have been established as the most reliable marker for predicting MTCT.[37] However, in resource-limited countries where access to HBV DNA testing is limited, the WHO conditionally recommended the use of HBeAg as an alternative indicator to determine eligibility for PAP.[5] Although positive HBeAg is closely correlated with elevated HBV DNA levels in HBV-infected pregnant women,[6] a subset of women may still carry high viral loads despite having negative HBeAg. This can occur because of the emergence of viral mutations that reduce HBeAg production while maintaining viral replication capacity.[38] To evaluate the independent role of HBV DNA levels and HBeAg status in relation to MTCT risk, we examined the proportion of women with high HBV DNA levels but negative HBeAg and assessed their risk of MTCT. Among the four groups cross-stratified by these two markers, the subgroup identified as 'high viral loads and negative HBeAg' represented the smallest proportion (2.70%, 95% CI 1.88–3.86%). Moreover, this specific subgroup, characterized by 'high viral loads and negative HBeAg', appeared to have a lower risk of MTCT than the group with 'high viral loads and positive HBeAg. Specifically, the rates of MTCT were 6.94 *vs.* 14.86% after HepB-BD only, 2.50 *vs.* 5.50% after HepB-BD + HBIG, and 0.00 *vs.* 1.32% after HepB-BD + HBIG + PAP, respectively. It is important to emphasize that these estimates were derived from a limited number of studies with a small sample size. Despite this limitation, the findings suggest that the potential impact of overlooking women who were HBeAg-negative with high HBV DNA levels, as a result of HBeAg-guided risk stratification, may have a relatively limited contribution compared with the oversight of women who were HBeAg-positive with similar virological profiles.

To effectively eliminate the MTCT of HBV, it is crucial to expand PMTCT interventions by integrating HBV screening, risk stratification, and administration of PAP into routine antenatal care.[5] In addition, integrating HBV PMTCT efforts with other infectious disease control programs targeting HIV or syphilis may further enhance the overall effectiveness.[7] However, in resource-limited countries, the available options for risk stratification following positive HBsAg screening should be carefully considered. The conventional HBV DNA-guided strategy is expected to

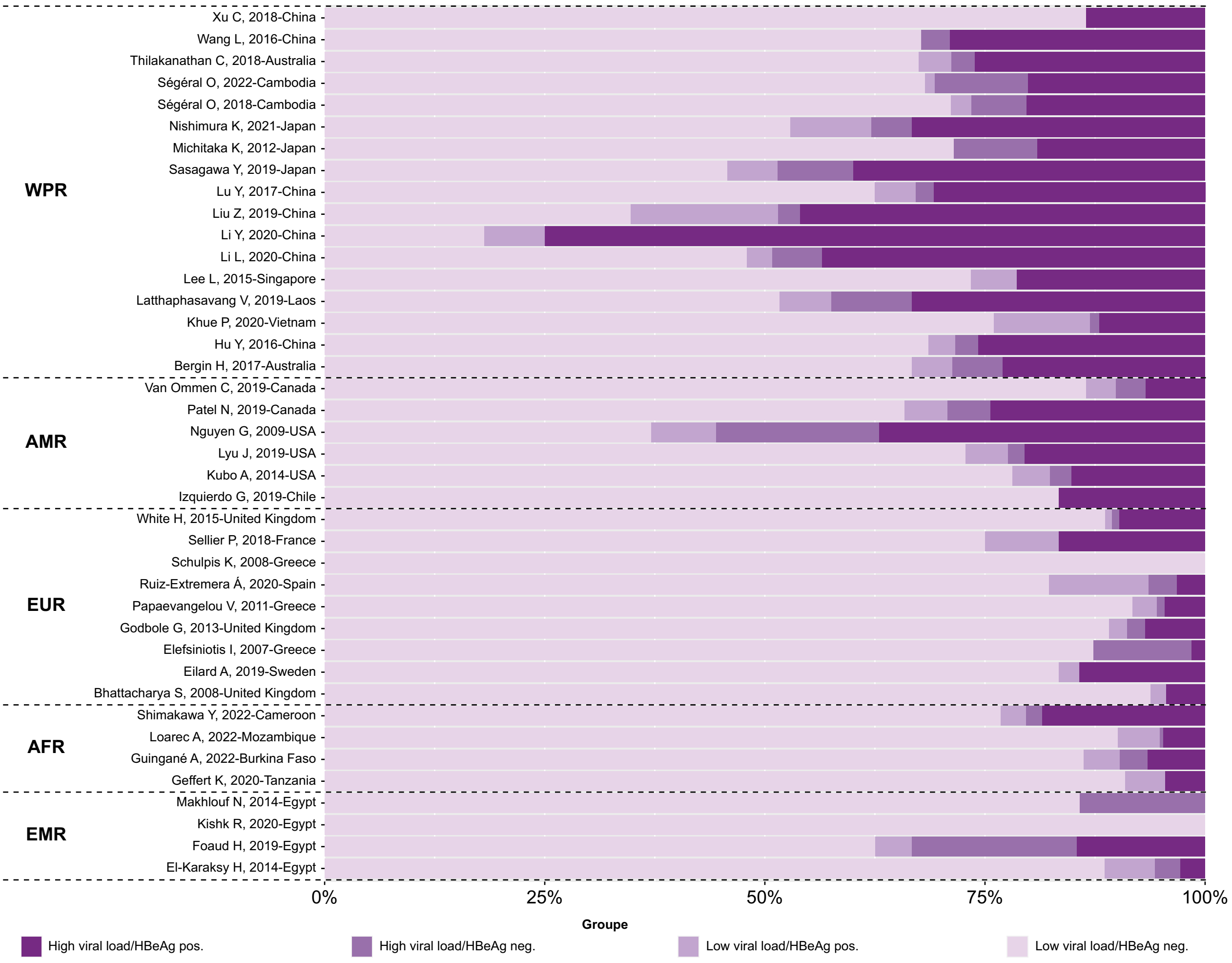

Fig. 4. Proportion of pregnant women with HBV infection in subgroups defined by both HBeAg status and HBV DNA levels. Proportions were pooled via a random-effects meta-analysis using a generalized linear mixed model with a logit link approach. AFR, African Region; AMR, Regions of the Americas; EMR, Eastern Mediterranean Region; EUR, European Region; WPR, Western Pacific Region.

be highly reliable, but limited access to this test in such contexts may raise concerns about its feasibility. Similarly, the alternative strategy guided by HBeAg detection, using laboratory-based immunoassays, faces challenges in accessibility, especially in decentralized resource-limited settings. The use of a rapid diagnostic test to detect HBeAg, although having limitations in analytical sensitivity, could potentially be improved by coupling it with alanine transaminase levels, thereby enhancing its diagnostic sensitivity.[39] Moreover, emerging biomarkers, such as a rapid test for hepatitis B core-related antigen (HBcrAg), provide additional options for risk assessment.[40] As an ultimate form of simplification, the universal administration of PAP to all women who were HBsAg-positive, without any risk stratification, has recently been proposed.[41] However, its feasibility and acceptability are currently unknown. Given the regional heterogeneity in the proportion of women eligible for PAP, the varying availability of different tests, and their associated costs across different countries, the findings of our study can provide valuable insights to help each country define the most optimal strategy for effectively eliminating HBV MTCT.

Our study has limitations. First, owing to the limited number of studies evaluating viral genotypes or coinfection with HCV or HDV, we were unable to perform subgroup analyses based on these factors. Methodological heterogeneity was also identified as a potential limitation, particularly regarding the comprehensive description of the HBsAg screening process. The spectrum and prevalence of both high HBV DNA levels and HBeAg may differ between studies that exclusively recruited women newly identified as positive HBsAg and those that included women known to have chronic HBV infection. We observed that studies with a more detailed description of HBsAg screening tended to provide lower estimates, suggesting that the pooled estimates in our study may be slightly overestimated compared with the general population of pregnant women. We conducted a systematic review of observational studies without any language restriction. However, the inclusion of Chinese-language articles posed challenges, as patient groups reported in these articles often overlapped with those in English-language articles, making it challenging to ascertain their uniqueness.[9] As a result, we opted not to include Chinese-language articles in our systematic review. Of note, this decision is unlikely to impact our work, given the substantial number of included studies conducted in China (n = 48). Lastly, we encountered a limited number of studies for the Q4, which examined the risk of MTCT based on maternal groups stratified by HBV DNA levels and HBeAg status. The scarcity of these

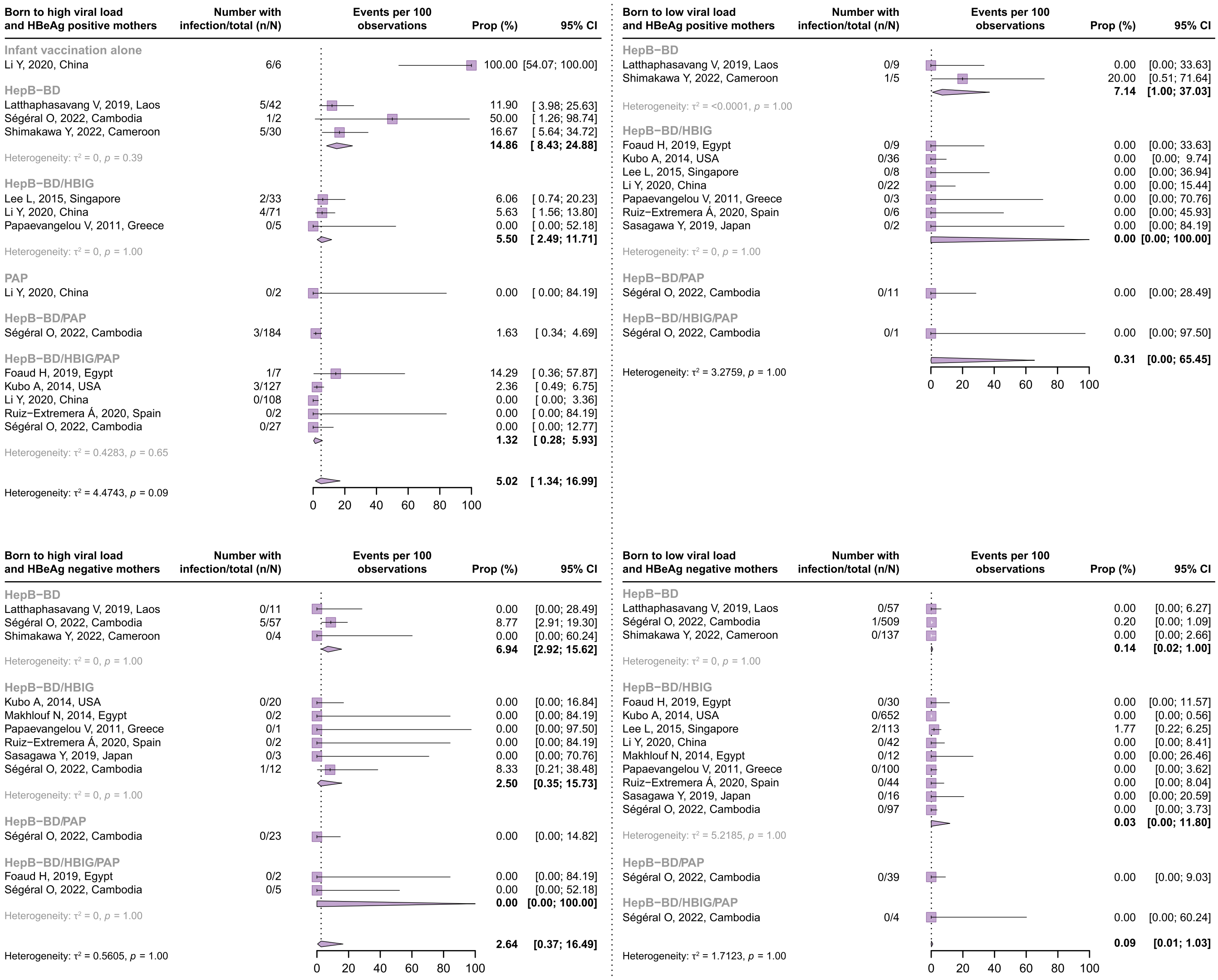

Fig. 5. Risk of child infection in subgroups defined by maternal HBeAg status and HBV DNA levels. Proportions were pooled via a random-effects meta-analysis using a generalized linear mixed model with a logit link approach. HBIG, hepatitis B immune globulin; HepB-BD, hepatitis B birth dose vaccine; PAP, peripartum antiviral prophylaxis.

studies was compounded by the need for additional stratification considering the type of maternal antiviral prophylaxis and infant immunoprophylaxis. We therefore could not examine the regional difference in the risk of MTCT. The limited availability of data highlights the need for additional research to further elucidate the potential role of HBeAg in modifying the risk of MTCT, independently of HBV DNA levels.[37]

In conclusion, our study found that approximately one-fifth of pregnant women with HBV-infection are eligible for PAP. There is a significant variation in this proportion between the WPR and the rest of the world. These findings underscore the crucial need to optimize strategies for expanding PMTCT interventions, considering the regional differences in the proportion of high-risk women. By integrating these interventions and tailoring them to local contexts, countries can make substantial progress toward the goal of MTCT elimination.

Abbreviations

AFR, African Region; AMR, Region of the Americas; EMR, Eastern Mediterranean Region; EUR, European Region; HBIG, hepatitis B immune globulin; HepB-BD, hepatitis B birth dose vaccine; LMICs, low- and middle-income countries; MTCT, mother-to-child transmission; PAP, peripartum antiviral prophylaxis; PMTCT, prevention of MTCT; SEAR, South-East Asia Region; WHO, World Health Organization; WPR, Western Pacific Region.

Financial support

There was no funding source for this study.

Conflicts of interest

YS has received a research grant from Gilead and research materials from Abbott Laboratories and Fujirebio Inc.

Please refer to the accompanying ICMJE disclosure forms for further details.

Authors' contributions

Conceived the study: YS. Developed the study protocol: MC, YS. Screened the titles and abstracts: MC, AR. Extracted the data: HD, JEIR, AR. Performed the statistical analysis: HD and JEIR. Contributed to supervision: JPV, YS. Provided technical support to complete the study: OS, SN. Wrote the first draft of the manuscript: HD, JEIR, YS. Had full access to all the data in the study, read and approved the final version of the manuscript, and had final responsibility for the decision to submit for publication: all authors.

Data availability statement

The full search strategy and key results used to generate data that inform the conclusion of this systematic review can be found in the Supplementary information.

Acknowledgements

JPV was funded by the Agence Nationale de Recherche sur le SIDA, les Hépatites Virales et les Maladies Infectieuses Émergentes (ANRS-MIE). We would like to extend our gratitude to the corresponding authors of the included studies who responded to our inquiries.

Supplementary data

Supplementary data to this article can be found online at https://doi.org/10.1016/j.jhepr.2024.101064.

References

[1] GBD 2019 Hepatitis B Collaborators. Global, regional, and national burden of hepatitis B, 1990–2019: a systematic analysis for the Global Burden of Disease Study 2019. Lancet Gastroenterol Hepatol 2022;7:796–829.
[2] Polaris Observatory Collaborators. Global prevalence, treatment, and prevention of hepatitis B virus infection in 2016: a modelling study. Lancet Gastroenterol Hepatol 2018;3:383–403.
[3] World Health Organization. Global progress report on HIV, viral hepatitis and sexually transmitted infections, 2021: accountability for the global health sector strategies 2016–2021: actions for impact: web annex 2: data methods. World Health Organization; 2021; https://apps.who.int/iris/handle/10665/342813. [Accessed 5 April 2023].
[4] Shimakawa Y, Yan H-J, Tsuchiya N, et al. Association of early age at establishment of chronic hepatitis B infection with persistent viral replication, liver cirrhosis and hepatocellular carcinoma: a systematic review. PLoS One 2013;8:e69430.
[5] Prevention of mother-to-child transmission of hepatitis B virus: guidelines on antiviral prophylaxis in pregnancy. https://www.who.int/publications-detail-redirect/978-92-4-000270-8 Accessed 5 April 2023.
[6] Boucheron P, Lu Y, Yoshida K, et al. Accuracy of HBeAg to identify pregnant women at risk of transmitting hepatitis B virus to their neonates: a systematic review and meta-analysis. Lancet Infect Dis 2021;21:85–96.
[7] Cohn J, Owiredu MN, Taylor MM, et al. Eliminating mother-to-child transmission of human immunodeficiency virus, syphilis and hepatitis B in sub-Saharan Africa. Bull World Health Organ 2021;99:287–295.
[8] **Ansari A**, **Vincent JP**, Moorhouse L, et al. Risk of early horizontal transmission of hepatitis B virus in children of uninfected mothers in sub-Saharan Africa: a systematic review and meta-analysis. Lancet Glob Health 2023;11:e715–e728.
[9] Zhou Y-H. Prevention of mother-to-child transmission of hepatitis B virus by treating mothers with high viral loads. Hepatology 2016;64:1823–1824.
[10] Hoy D, Brooks P, Woolf A, et al. Assessing risk of bias in prevalence studies: modification of an existing tool and evidence of interrater agreement. J Clin Epidemiol 2012;65:934–939.
[11] Altman DG. Systematic reviews in health care. London: BMJ Publishing Group; 2001.
[12] Hunter JP, Saratzis A, Sutton AJ, et al. In meta-analyses of proportion studies, funnel plots were found to be an inaccurate method of assessing publication bias. J Clin Epidemiol 2014;67:897–903.
[13] Kfutwah AK, Tejiokem MC, Njouom R. A low proportion of HBeAg among HBsAg-positive pregnant women with known HIV status could suggest low perinatal transmission of HBV in Cameroon. Virol J 2012;9:62.
[14] Rouet F, Chaix M-L, Inwoley A, et al. HBV and HCV prevalence and viraemia in HIV-positive and HIV-negative pregnant women in Abidjan, Côte d'Ivoire: the ANRS 1236 study. J Med Virol 2004;74:34–40.
[15] Thumbiran NV, Moodley D, Parboosing R, et al. Hepatitis B and HIV coinfection in pregnant women: indication for routine antenatal hepatitis B virus screening in a high HIV prevalence setting. S Afr Med J 2014;104:307.
[16] Keel P, Edwards G, Flood J, et al. Assessing the impact of a nurse-delivered home dried blood spot service on uptake of testing for household contacts of hepatitis B-infected pregnant women across two London trusts. Epidemiol Infect 2016;144:2087–2097.
[17] Lyu J, Wang S, He Q, et al. Hep B moms: a cross-sectional study of mother-to-child transmission risk among pregnant Asian American women with chronic hepatitis B in New York City, 2007–2017. J Viral Hepat 2020;27:168–175.
[18] Nguyen G, Garcia RT, Nguyen N, et al. Clinical course of hepatitis B virus infection during pregnancy. Aliment Pharmacol Ther 2009;29:755–764.
[19] Bzowej NH, Tran TT, Li R, et al. Total alanine aminotransferase (ALT) flares in pregnant North American women with chronic hepatitis B infection: results from a prospective observational study. Am J Gastroenterol 2019;114:1283–1291.
[20] Patel NH, Joshi SS, Lau KCK, et al. Analysis of serum hepatitis B virus RNA levels in a multiethnic cohort of pregnant chronic hepatitis B carriers. J Clin Virol 2019;111:42–47.
[21] Fujiko M, Chalid MT, Turyadi, et al. Chronic hepatitis B in pregnant women: is hepatitis B surface antigen quantification useful for viral load prediction? Int J Infect Dis 2015;41:83–89.
[22] Geffert K, Maponga TG, Henerico S, et al. Prevalence of chronic HBV infection in pregnant woman attending antenatal care in a tertiary hospital in Mwanza, Tanzania: a cross-sectional study. BMC Infect Dis 2020;20:395.
[23] Kishk R, Mandour M, Elprince M, et al. Pattern and interpretation of hepatitis B virus markers among pregnant women in North East Egypt. Braz J Microbiol 2020;51:593–600.
[24] Schulpis KH, Barzeliotou A, Papadakis M, et al. Maternal chronic hepatitis B virus is implicated with low neonatal paraoxonase/arylesterase activities. Clin Biochem 2008;41:282–287.
[25] Chakvetadze C, Roussin C, Roux J, et al. Efficacy of hepatitis B sero-vaccination in newborns of African HBsAg positive mothers. Vaccine 2011;29:2846–2849.
[26] Li Y, Wang J, Yu Y, et al. Maternal antiviral treatment safeguards infants from hepatitis B transmission in contingencies of delayed immunoprophylaxis. Liver Int 2020;40:2377–2384.
[27] **Liu J**, **Chen T**, **Chen Y**, et al. 2019 Chinese clinical practice guidelines for the prevention of mother-to-child transmission of hepatitis B virus. J Clin Transl Hepatol 2020;8:397–406.
[28] Giri S, Sahoo S, Angadi S, et al. Seroprevalence of hepatitis B virus among pregnant women in India: a systematic review and meta-analysis. J Clin Exp Hepatol 2022;12:1408–1419.
[29] **Liu D**, **Liu Y**, **Ni J**, et al. Hepatitis B infection among pregnant women in China: a systematic review and meta-analysis. Front Public Health 2022;10:879289.
[30] Olakunde BO, Adeyinka DA, Olakunde OA, et al. A systematic review and meta-analysis of the prevalence of hepatitis B virus infection among pregnant women in Nigeria. PLoS One 2021;16:e0259218.
[31] Liu X, Chen C, Jiang D, et al. Comparison of HBV-DNA and HBeAg as antiviral therapeutic indicators among HBV-infected pregnant women: a systematic review and meta-analysis. Ann Palliat Med 2021;10:9362–9371.
[32] Bigna JJ, Kenne AM, Hamroun A, et al. Gender development and hepatitis B and C infections among pregnant women in Africa: a systematic review and meta-analysis. Infect Dis Poverty 2019;8:16.
[33] Ott JJ, Stevens GA, Wiersma ST. The risk of perinatal hepatitis B virus transmission: hepatitis B e antigen (HBeAg) prevalence estimates for all world regions. BMC Infect Dis 2012;12:131.
[34] Moutchia J, Njouom R, Rumpler E, et al. Maternal age at first childbirth and geographical variation in hepatitis B virus prevalence in Cameroon: important role of mother-to-child transmission. Clin Infect Dis 2022;74:836–845.
[35] Yang Y, Huang A, Zhao Y. Spontaneous loss of chronic HBV infection markers in treatment-naïve children: a systematic review and pooled meta-analyses. Expert Rev Anti Infect Ther 2021;19:649–660.

[36] Mohareb AM, Liu AF, Kim AY, et al. Clearance of hepatitis B e antigen in untreated chronic hepatitis B virus infection: a systematic review and meta-analysis. J Infect Dis 2022;226:1761–1770.
[37] Pan CQ, Duan Z-P, Bhamidimarri KR, et al. An algorithm for risk assessment and intervention of mother to child transmission of hepatitis B virus. Clin Gastroenterol Hepatol 2012;10:452–459.
[38] Kramvis A. The clinical implications of hepatitis B virus genotypes and HBeAg in pediatrics. Rev Med Virol 2016;26:285–303.
[39] Segeral O, Dim B, Durier C, et al. Hepatitis B e antigen (HBeAg) rapid test and alanine aminotransferase level–based algorithm to identify pregnant women at risk of HBV mother-to-child transmission: the ANRS 12345 TA PROHM study. Clin Infect Dis 2020;71:e587–e593.
[40] Shimakawa Y, Ndow G, Kaneko A, et al. Rapid point-of-care test for hepatitis B core-related antigen to diagnose high viral load in resource-limited settings. Clin Gastroenterol Hepatol 2023;21(7):1943–1946.e2.
[41] Nayagam S, de Villiers MJ, Shimakawa Y, et al. Impact and cost-effectiveness of hepatitis B virus prophylaxis in pregnancy: a dynamic simulation modelling study. Lancet Gastroenterol Hepatol 2023;8:635–645.

Journal of Hepatology, Volume *6*

Supplemental information

Proportion of pregnant women with HBV infection eligible for antiviral prophylaxis to prevent vertical transmission: A systematic review and meta-analysis

Hugues Delamare, Julian Euma Ishii-Rousseau, Adya Rao, Mélanie Cresta, Jeanne Perpétue Vincent, Olivier Ségéral, Shevanthi Nayagam, and Yusuke Shimakawa

Supplementary material

Proportion of pregnant women with HBV infection eligible for antiviral prophylaxis to prevent vertical transmission: A systematic review and meta-analysis

Hugues Delamare, Julian Euma Ishii-Rousseau, Adya Rao, Mélanie Cresta, Jeanne Perpétue Vincent, Olivier Ségéral, Shevanthi Nayagam, Yusuke Shimakawa

Contents

Supplementary Methods 1. Search strategy ... 3

Supplementary Methods 2. List of variables on the data extraction sheet ... 7

Supplementary Methods 3. Risk of bias assessment tool for Q1, Q2, and Q3 (Hoy D et al., 2012) ... 11

Supplementary Methods 4. Risk of bias assessment tool for Q4 (Altman D, 2001) ... 15

Supplementary Results 1. List of articles with overlapping study population ... 17

Supplementary Results 2. Study characteristics ... 19

Supplementary Results 2A. Characteristics of the included studies ... 19

Supplementary Results 2B. Characteristics of the included cohorts in Q4 ... 32

Supplementary Results 3. Risk of bias ... 35

Supplementary Results 3A. Risk of bias of the studies included in Q1, Q2, and Q3 ... 35

Supplementary Results 3B. Risk of bias of the studies included in Q4 ... 42

Supplementary Results 4. Publication bias ... 44

Supplementary Results 4A. Adapted funnel plots for the studies included in Q1 (n=67) ... 44

Supplementary Results 4B. Adapted funnel plots for the studies included in Q2 (n=125) ... 44

Supplementary Results 5. Description of studies that provided outlying estimates ... 45

Supplementary Results 5A. Studies showing substantial deviation towards lower estimates (n=13) ... 45

Supplementary Results 5B. Studies showing substantial deviation towards higher estimates (n=18) ... 46

Table S1. Subgroup analyses for the proportion of HBV-infected pregnant women with high HBV DNA levels (67 cohorts from 67 studies) ... 47

Table S2. Subgroup analyses for the proportion of HBV-infected pregnant women who test positive for HBeAg (129 cohorts from 125 studies) ... 49

Fig. S1. Proportion of HBV-infected pregnant women in subgroups defined by both HBV DNA levels and HBeAg status according to the WHO regions ... 51

Fig. S1A. Proportion of HBV-infected pregnant women with high viral load and1 positive HBeAg 51
Fig. S1B. Proportion of HBV-infected pregnant women with high viral load and negative HBeAg 52
Fig. S1C. Proportion of HBV-infected pregnant women with low viral load and positive HBeAg 53
Fig. S1D. Proportion of HBV-infected pregnant women with low viral load and negative HBeAg 54
Supplementary references 1. References of studies included in the systematic review 55

Supplementary Methods 1. Search strategy

Database: PubMed
Date searched: From January 1st, 2000 to June 22nd, 2021

Item	Search words
**1**	"hepatitis b"[MeSH] OR "hepatitis b virus"[MeSH]
**2**	hepatitis b[Text] OR type b hepatitis[Text] OR hepatitis type b[Text] OR hbv[Text] OR vhb[Text] OR hep b[Text] OR hbsag[Text] OR hbs ag[Text] OR hbs antigen*[Text]
**3**	#1 OR #2
**4**	“viral load”[MeSH] OR “viremia”[MeSH] OR “DNA, viral”[MeSH] OR “nucleic acid amplification techniques”[MeSH]
**5**	viral load*[Text] OR viremi*[Text] OR viraemi*[Text] OR DNA[Text] OR nucleic acid test*[Text] OR nucleic acid amplification*[Text] OR NAT[Text] OR polymerase chain reaction*[Text] OR PCR[Text]
**6**	#4 OR #5
**7**	“pregnancy”[MeSH] OR “pregnant women”[MeSH] OR “maternal- fetal relations”[MeSH] OR “infectious disease transmission, vertical”[MeSH] OR “pregnancy complications, infectious”[MeSH] OR “prenatal diagnosis”[MeSH]
**8**	pregnan*[Text] OR trimest*[Text] OR gestation*[Text] OR antepartum[Text] OR ante-partum[Text] OR prepartum[Text] OR prepartum[Text] OR intrapartum[Text] OR intra-partum[Text] OR peripartum[Text] OR peri-partum[Text] OR antenatal*[Text] OR antenatal*[Text] OR prenatal*[Text] OR pre-natal*[Text] OR perinatal*[Text] OR peri-natal*[Text] OR intrauterine[Text] OR intra-uterine[Text] OR inutero[Text] OR in utero[Text] OR transplacental*[Text] OR placenta*[Text] OR vertical*[Text] OR congenital*[Text] OR mother*[Text] OR matern*[Text] OR fetomaternal*[Text] OR foetomaternal*[Text] OR fetal*[Text] OR foetal*[Text] OR fetus[Text] OR foetus[Text] OR offspring[Text] OR MTCT[Text] OR TME[Text]
**9**	#7 OR #8
**10**	#3 AND #6 AND #9

Database: Embase
Date searched: From January 1st, 2000 to June 22nd, 2021

Item	Search words
1	'hepatitis b'/exp OR 'hepatitis b' OR 'hepatitis b virus'/exp OR 'hepatitis b virus'
2	'hepatitis b' OR 'type b hepatitis' OR 'hepatitis type b' OR 'hbv' OR 'vhb' OR 'hep b' OR 'hbsag' OR 'hbs ag' OR 'hbs antigen'
3	#1 OR #2
4	'virus load'/exp OR 'viremia'/exp OR 'virus dna'/exp OR 'nucleic acid amplification'/exp
5	'viral load' OR 'viremi' OR 'viraemi' OR 'dna' OR 'nucleic acid test' OR 'nucleic acid amplification' OR 'nat' OR 'polymerase chain reaction' OR 'pcr'
6	#4 OR #5
7	'pregnancy'/exp OR 'pregnant women'/exp OR 'mother fetus relationship'/exp OR 'vertical transmission'/exp OR 'pregnancy complication'/exp OR 'prenatal diagnosis'/exp
8	'pregnan' OR 'trimest' OR 'gestation' OR 'antepartum' OR 'prepartum' OR 'pre-partum' OR 'intrapartum' OR 'peripartum' OR 'peri-partum' OR 'antenatal' OR 'ante-natal' OR 'prenatal' OR 'pre-natal' OR 'perinatal' OR 'peri-natal' OR 'intrauterine' OR 'intra-uterine' OR 'inutero' OR 'in utero' OR 'transplacental' OR 'placenta' OR 'vertical' OR 'congenital' OR 'mother' OR 'matern' OR 'fetomaternal' OR 'foetomaternal' OR 'fetal' OR 'foetal' OR 'fetus' OR 'foetus' OR 'offspring' OR 'mtct' OR 'tme'
9	#7 OR #8
10	#3 AND #6 AND #9

Database: Scopus
Date searched: From January 1st, 2000 to June 22nd, 2021

Item	Search words
1	TITLE-ABS-KEY (“hepatitis b” OR “type b hepatitis” OR “hepatitis type b” OR “hbv” OR “vhb” OR “hep b” OR “hbsag” OR “hbs ag” OR “hbs antigen*”)
2	TITLE-ABS-KEY (“viral load*” OR “viremi*” OR “viraemi*” OR “DNA” OR “nucleic acid test*” OR “nucleic acid amplification*” OR “NAT” OR “polymerase chain reaction*” OR “PCR”)
3	TITLE-ABS-KEY (“pregnan*” OR “trimest*” OR “gestation*” OR “antepartum” OR “ante-partum” OR “prepartum” OR “pre-partum” OR “intrapartum” OR “intra-partum” OR “peripartum” OR “peripartum” OR “antenatal*” OR “ante-natal*” OR “prenatal*” OR “prenatal*” OR “perinatal*” OR “peri-natal*” OR “intrauterine” OR “intrauterine” OR “inutero” OR “in utero” OR “transplacental*” OR “placenta*” OR “vertical*” OR “congenital*” OR “mother*” OR “matern*” OR “fetomaternal*” OR “foetomaternal*” OR “fetal*” OR “foetal*” OR “fetus” OR “foetus” OR “offspring” OR “MTCT” OR “TME”)
4	#1 AND #2 AND #3

Database: CENTRAL Database (The Cochrane Library)
Date searched: From January 1st, 2000 to June 22nd, 2021

Item	Search words
1	hepatitis b (MeSH, exp) OR hepatitis b virus (MeSH, exp)
2	"hepatitis b" OR "type b hepatitis" OR "hepatitis type b" OR hbv OR vhb OR "hep b" OR hbsag OR “hbs ag” OR “hbs antigen” OR “hbs antigens”
3	#1 OR #2
4	“viral load”(MeSH, exp) OR “viremia”(MeSH, exp) OR “DNA, viral”(MeSH, exp) OR “nucleic acid amplification techniques”(MeSH, exp)
5	“viral load*” OR viremi* OR viraemi* OR DNA OR “nucleic acid test*” OR “nucleic acid amplification*” OR NAT OR “polymerase chain reaction*” OR PCR
6	#4 OR #5
7	pregnancy (MeSH, exp) OR pregnant women (MeSH, exp) OR maternal-fetal relations (MeSH, exp) OR infectious disease transmission, vertical (MeSH, exp) OR pregnancy complications, infectious (MeSH, exp) OR prenatal diagnosis (MeSH, exp)
8	pregnan* OR trimest* OR gestation* OR antepartum OR antepartum OR prepartum OR pre-partum OR intrapartum OR intrapartum OR peripartum OR peri-partum OR antenatal* OR ante-natal* OR prenatal* OR pre-natal* OR perinatal* OR peri-natal* OR intrauterine OR intra-uterine OR inutero OR “in utero” OR transplacental* OR placenta* OR vertical* OR congenital* OR mother* OR matern* OR fetomaternal* OR foetomaternal* OR fetal* OR foetal* OR fetus OR foetus OR offspring* OR MTCT OR TME
9	#7 OR #8
10	#3 AND #6 AND #9

Supplementary Methods 2. List of variables on the data extraction sheet

Publication details
- Year
- Language
- Journal
- First Author
- Last Author

Methods
Study setting
- Country
- Study main objective
- Study design
- Recruitment setting (center or regional details, number of study sites)
- Recruitment period
- Inclusion criteria
- Exclusion criteria

- Concomitant anti-HBV therapy at baseline of pregnancy
- Selection based on newborns with HBIG completed
- Risk of biased sampling
- Data per pregnancy
- Overlap
- GroupID

Maternal HBV DNA
- HBV DNA level availability
- Maternal HBV DNA: Type of sample
- Maternal HBV DNA: When sample was taken
- Maternal HBV DNA: Type of assay
- Maternal HBV DNA: Qualitative or quantitative

Maternal HBeAg
- HBeAg status availability
- Maternal HBeAg: Type of sample
- Maternal HBeAg: When sample was taken
- Maternal HBeAg: Was this measured on the same sample as HBV DNA?
- Maternal HBeAg: Type of assay
- Maternal HBeAg: Commercial name of assay
- Maternal HBeAg: Qualitative or quantitative
- Maternal HBeAg: Limit of detection of the assay used (PEIU/mL, etc).

Women's characteristics
No. of participating women
- No. of women eligible for HBsAg screening
- No. of women screened for HBsAg (*have included HBsAg+ women, prior to exclusion)
- No. of women tested positive for HBsAg (*i.e. number included for analysis after excluding certain HBsAg+ women for other reasons)
- No. of women who had HBV DNA tested
- No. of women who had HBeAg tested
- No. of women who had both HBV DNA & HBeAg tested
- Mean (SD) or median (IQR) maternal age

- Numerator/denominator by HBV viral genotypes
- Numerator/denominator by HIV status
- Numerator/denominator by HCV status
- Numerator/denominator by HDV status
- High viral load cut off (only for studies providing numerators for HVL)

Proportion of HBV-infected pregnant women with high HBV DNA levels ≥200,000 IU/ml, with a positive hepatitis B e antigen (HBeAg) and with a discordant result

- Numerator: No. with high VL
- Denominator: No. of women tested positive for HBsAg and tested for viral load
- Numerator: No. with HBeAg-pos
- Denominator:No. of women tested positive for HBsAg and tested for HbeAg
- Numerator: No. with high VL & HBeAg-pos
- Numerator: No. with high VL & HBeAg-neg
- Numerator: No. with low VL & HBeAg-neg
- Numerator: No. with low VL & HBeAg-pos
- Denominator: No. of women tested positive for HBsAg and tested for both viral load & HBeAg
- No. of women with indeterminate result for HBeAg

<u>Infants' characteristics</u>
MTCT criteria

- Clinical endpoint for MTCT (i.e., HBsAg-positivity or HBV DNA positivity in infants at the age of 6-12 months is reported (Yes/no))

Infant HBsAg (only those reporting MTCT endpoint)

- Infant HBsAg: Type of sample
- Infant HBsAg: When sample was taken
- Infant HBsAg: Type of assay

Infant HBV DNA (only those reporting MTCT endpoint)

- Infant HBV DNA: Type of sample
- Infant HBV DNA: When sample was taken
- Infant HBV DNA: Type of assay
- Infant HBV DNA: Qualitative or quantitative
- Infant HBV DNA: Limit of detection of the assay used (IU/ml or copies/ml)

Preventive measures (only those reporting MTCT endpoint)

- Birth dose vaccine
- Age at birth dose vaccine (eg, <24h?)
- Infant vaccine
- No. of doses of infant vaccine
- Age at first dose of infant vaccine
- Age at second dose of infant vaccine
- Age at third dose of infant vaccine
- HBIG
- No. of doses of HBIG
- Age at HBIG
- Peripartum antiviral prophylaxis
- Name of antiviral used for peripartum antiviral prophylaxis
- When peripartum antiviral prophylaxis started?
- When peripartum antiviral prophylaxis stopped?
- Peripartum antiretroviral therapy for women co-infected with HIV
- Antiretroviral HIV therapy effective on HBV

- Name of antiretroviral, effective for HBV, used during pregnancy
- When were the antiretroviral prophylaxis, effective for HBV, started?
- When were the antiretroviral prophylaxis, effective for HBV, stopped?
- All participants receive the same preventive strategy for MTCT?

Q4: risk of MTCT in subgroups of HBV-infected mothers with a discordant result stratified by any measure to prevent HBV MTCT, including birth dose vaccine and/or 2-3 doses of infant vaccine, HBIG, peripartum antiviral prophylaxis and peripartum antiretroviral therapy for women co-infected with HIV.

- Child outcome used for this LINE (HBsAg or HBV DNA)
- PMTCT intervention used for this LINE (HepB-BD/HepB3/HBIG/PAP/Antiretroviral effective for HBV)
- Numerator: No. of infants born to HBsAg-pos mothers with high VL & HBeAg-pos, who were positive for child outcome
- Denominator: No. of infants born to HBsAg-pos mothers with high VL & HBeAg-pos, who were tested for child outcome
- Numerator: No. of infants born to HBsAg-pos mothers with high VL & HBeAg-neg, who were positive for child outcome
- Denominator: No. of infants born to HBsAg-pos mothers with high VL & HBeAg-neg, who were tested for child outcome
- Numerator: No. of infants born to HBsAg-pos mothers with low VL & HBeAg-neg, who were positive for child outcome
- Denominator: No. of infants born to HBsAg-pos mothers with low VL & HBeAg-neg, who were tested for child outcome
- Numerator: No. of infants born to HBsAg-pos mothers with low VL & HBeAg-pos, who were positive for child outcome
- Denominator: No. of infants born to HBsAg-pos mothers with low VL & HBeAg-pos, who were tested for child outcome

<u>Other</u>

- Funding by industry
- Comments
- Eligibility
- Eligibility comment
- Reason for non-eligibility

<u>Risk of bias Q1, Q2 & Q3</u>

External validity

- Was the study's target population a close representation of the national population in relation to relevant variables?
- Was the sampling frame a true or close representation of the target population?
- Was some form of systematic, random selection used to select the sample, or was a census undertaken?
- Was the likelihood of selecting women under concomitant antiviral treatment at baseline evaluation minimal?
- Was the likelihood of non-response bias minimal for HBeAg test? (75%)
- Was the likelihood of non-response bias minimal for HBV DNA test?

Internal validity

- Were data collected directly from the subjects (as opposed to a proxy)?
- Was the study instrument that measured the parameter of interest (HBeAG) shown to have validity and reliability?
- Was the same mode of data collection used for all subjects?
- Were the numerator(s) and denominator(s) for HBeAg appropriate?

- Were the numerator(s) and denominator(s) for HBV DNA appropriate?
- Summary item on the overall risk of study bias
- Rationale

<u>Risk of bias Q4</u>

1. Sample of patients
    - Eligibility criteria defined
    - Sample selection explained (setting, locations and periods of recruitment)
    - Clinical and demographic characteristics fully described
    - Representative of review question population (i.e. recruited following positive HBsAg results at antenatal care)
    - Completeness (of the women HBsAg+ eligible for the study, how many were included?)

2. Outcome (MTCT)
    - HBsAg or HBV DNA assay in infants fully defined
    - Proportion of infants born to enrolled HBsAg-positive mothers with HBV DNA and HbeAg assessment who were assessed for outcome
    - Outcome assessor blinded to maternal HBV DNA levels
    - Outcome assessor blinded to maternal HBeAg status

3. Exposure (Maternal viral load)
    - HBV DNA assay in pregnant women fully defined
    - Proportion of enrolled HBsAg-positive mothers who were assessed for HBV DNA
    - Assessor of maternal HBV DNA blinded to child outcome status
    - Assessor of maternal HBV DNA blinded to maternal HBeAg status
    - HBeAg assay in pregnant women fully defined
    - Proportion of enrolled HBsAg-positive mothers who were assessed for HBeAg
    - Assessor of maternal HBeAg blinded to child outcome status
    - Assessor of maternal HBeAg blinded to maternal HBV DNA levels

4. PMTCT strategy subsequent to inclusion in cohort
    - Fully described for hepatitis B vaccine and its schedule
    - Fully described for HBIG and its schedule
    - Fully described for peripartum antiviral prophylaxis and its timing

Supplementary Methods 3. Risk of bias assessment tool for Q1, Q2, and Q3 (Hoy D et al., 2012)

Risk of bias item	Criteria for answers (please circle one option)	Additional notes and examples
External validity		
1. Was the study's target population (i.e. pregnant women) **a close representation** of the national population in relation to relevant variables?	● **Yes (LOW RISK):** The study's target population was a close representation of the national population.<br>● **No (HIGH RISK)**: The study's target population was clearly NOT representative of the national population.<br>● **Not reported (UNKNOWN RISK)**	The **target population** refers to the group of people or entities to which the results of the study will be generalized. Examples:<br>● The study was a national survey of pregnant women, and the sample was drawn from a list that included all individuals who made antenatal care. The answer is: **Yes (LOW RISK).**<br>● The study was conducted in one province only, and it is not clear if this was representative of the national population. The answer is: **No (HIGH RISK).**<br>● The study was undertaken in one hospital only and it is clear this was not representative of the national population. The answer is:
2. Was the sampling frame a **true or close representation** of the target population?	● **Yes (LOW RISK):** The sampling frame was a **true or close** representation of the target population.<br>● **No (HIGH RISK)**: The sampling frame was NOT a **true or close** representation of the target population.<br>● **Not reported (UNKNOWN RISK)**	The **sampling frame** is a list of the sampling units in the target population and the study sample is drawn from this list. Examples:<br>● The sampling was carried out in antenatal care services at primary healthcare or at community. The answer is: **Yes (LOW RISK)**.<br>● The sampling was carried out at tertiary care hospitals. The answer is: **No (HIGH RISK)**.
3. Was some form of **random selection** used to select the sample (i.e., pregnant women), OR, was a census undertaken?	● **Yes (LOW RISK):** A census, OR, some form of random selection (e.g. simple random sampling, stratified random sampling, cluster sampling, systematic sampling), OR, consecutive sampling.<br>● **No (HIGH RISK)**:<br>A convenient sample was selected.<br>● **Not reported (UNKNOWN RISK)**	A census collects information from every unit in the sampling frame. In a survey, only part of the sampling frame is sampled. In these instances, random selection of the sample helps minimize study bias.<br>In a hospital-based study, consecutive sampling or systematic sampling can be acceptable.<br>Examples:<br>● The sample was selected using simple random sampling. The answer is: **Yes (LOW RISK)**.<br>● Every woman visiting antenatal care services were sampled. The answer is: **Yes (LOW RISK)**.<br>● Women were sampled only when their children completed infant immunoprophylaxis . The answer is: **No (HIGH RISK).**

4a.. Was the likelihood of **non-response bias minimal for HBeAg test?**	• **Yes (LOW RISK):** The response rate for the study (proportion of HBsAg-positive pregnant women who had HBeAg test) was >/=75%.<br>• **No (HIGH RISK)**: The response rate was <75%.	Examples:<br>• Of 100 HBsAg-positive women, 98 had HBeAg test. The response rate was 98%. The answer is: **Yes (LOW RISK)**.<br>• Of 100 HBsAg-positive women, only 65 had HBeAg test. The response rate was 65%. The answer is: **No (HIGH RISK)**.
4b. Was the likelihood of **non-response bias minimal for HBV DNA test?**	• **Yes (LOW RISK):** The response rate for the study (proportion of HBsAg-positive pregnant women who had HBV DNA test) was >/=75%.<br>• **No (HIGH RISK)**: The response rate was <75%,.<br>• **Not reported (UNKNOWN RISK)**	Examples:<br>• Of 100 HBsAg-positive women, 98 had HBV DNA test. The response rate was 98%. The answer is: **Yes (LOW RISK)**.<br>Of 100 HBsAg-positive women, only 65 had HBV DNA test. The response rate was 65%. The answer is: **No (HIGH RISK)**.
5. Was the likelihood of selecting women under concomitant antiviral treatment at baseline evaluation minimal?	• **Yes (LOW RISK):** Article specifies that no pregnant women were under antiviral therapy at the baseline evaluation.<br>• **No (HIGH RISK) :** Article does not specify that no pregnant women were under antiviral therapy at the baseline evaluation.	Pregnant women were under antiviral therapy at the baseline evaluation

Internal validity		
6. Were data collected **directly from the subjects** (as opposed to a proxy)?	• **Yes (LOW RISK):** All data were collected from hospital or laboratory records .	• All eligible subjects were tested for HBeAg and results were recorded in the laboratory. The answer is: **Yes (LOW RISK)**.

7. Was an acceptable case definition used in the study?	● **Yes (LOW RISK):** An acceptable case definition was used.<br>● **No (HIGH RISK)**: An acceptable case definition was NOT used.<br>● **Not reported (UNKNOWN RISK)**	For this systematic review, this question is **Not applicable (NA)**.
8a. Was the study instrument that measured HBeAg shown to have **reliability and validity** ?	● **Yes (LOW RISK):** The study used enzyme immunoassay (EIA) or chemiluminescent immunoassay (CLIA) to detect HBeAg .<br>● **No (HIGH RISK)**: The study used rapid diagnostic test (RDT) or other low sensitivity test to detect HBeAg.<br>● **Not reported (UNKNOWN RISK)**	● The authors used the CLIA to detect HBeAg (Architect, Abbott) . The answer is: **Yes (LOW RISK)**.<br>● The authors used the RDT to detect HBeAg (SD Bioline, Alere) . The answer is: **No (HIGH RISK)**.
8b. Was the study instrument that measured HBV DNA shown to have **reliability and validity**?	● **Yes (LOW RISK):** The study used commercially available RT-PCR.<br>● **No (HIGH RISK)**: The study used in-house PCR or other nucleic acid test.<br>● **Not reported (UNKNOWN RISK)**	● The authors used the RT-PCR (RealTime, Abbott). The answer is: **Yes (LOW RISK)**.<br>● The authors used an in-house RT-PCR without any methodological reference. The answer is: **No (HIGH RISK)**.
9a. Was the **same HBeAg assay** used for all subjects?	● **Yes (LOW RISK):** The same HBeAg assay was used for all subjects.<br>● **No (HIGH RISK)**: The same HBeAg assay was NOT used for all subjects.<br>● **Not reported (UNKNOWN RISK)**	
9b. Was the **same HBV DNA assay** used for all subjects?	● **Yes (LOW RISK):** The same HBV DNA assay was used for all subjects.<br>● **No (HIGH RISK)**: The same HBV DNA assay was NOT used for all subjects.<br>● **Not reported (UNKNOWN RISK)**	

10. Was the **length <u>of the</u> <u>shortest</u> <u>prevalence</u> <u>period</u>** for the parameter of interest appropriate?	• **Yes (LOW RISK):** The shortest prevalence period for the parameter of interest was appropriate (e.g. point prevalence, one-week prevalence, one-year prevalence).<br>• **No (HIGH RISK)**: The shortest prevalence period for the parameter of interest was not appropriate (e.g. lifetime prevalence)	For this systematic review, this question is **Not applicable (NA)**.
11. Were the **<u>numerator(s) and denominator(s)</u>** for the parameter of interest appropriate?	• **Yes (LOW RISK):** The paper reported the number of pregnant women.<br>• **No (HIGH RISK)**: The paper reported the number of mother-child pairs (i.e. counting a woman who gave birth to a twin twice) AND multiple pregnancies must be < 10% of total pregnancies if we can't exclude them..<br>• **Not reported (UNKNOWN RISK)**	One woman can give birth to a twin or a triplet, or one woman can visit antenatal care for two different episodes of pregnancy. By counting these women independently, the data will be correlated unless these are taken into account by a statistical analysis (e.g. mixed effect model).<br>• 100 HBsAg-positive women gave birth to 110 infants (10 twins). The prevalence of HBeAg in 100 HBsAg-positive women was 10% (10/100). The answer is: **Yes (LOW RISK)**.<br>• 100 HBsAg-positive women gave birth to 110 infants (10 twins). Of 110 infants, 15 infants were born to mothers positive for HBeAg. The prevalence of HBeAg was estimated as 13% (15/110). The answer is: **No (HIGH RISK).**

Supplementary Methods 4. Risk of bias assessment tool for Q4 (Altman D, 2001)

Study feature	Qualities sought	Assessment		
1. Sample of patients	Eligibility criteria defined	Yes	No	Unclear
	Sample selection explained (setting, locations and periods of recruitment)	Yes	No	Unclear
	Clinical and demographic characteristics fully described	Yes	No	Unclear
	Representative of review question population (i.e. recruited following positive HBsAg results at antenatal care)	Yes	No	Unclear
	Completeness (of the women HBsAg+ eligible for the study, how many were included?)	>75%	≤75%	Unclear
2. Outcome (MTCT)	HBsAg or HBV DNA assay in infants fully defined	Yes	No	Unclear
	Proportion of infants born to enrolled HBsAg-positive mothers with HBV DNA assessment who were assessed for outcome	>80%	≤80%	Unclear
3. Exposure (Maternal viral load and HBeAg status)	HBV DNA assay in pregnant women fully defined	Yes	No	Unclear
	Proportion of enrolled HBsAg-positive mothers who were assessed for HBV DNA	>75%	≤75%	Unclear
	HBeAg assay in pregnant women fully defined	Yes	No	Unclear
	Proportion of enrolled HBsAg-positive mothers who were assessed for HBeAg	>75%	≤75%	Unclear
4. PMTCT strategy subsequent to inclusion in cohort	Fully described for hepatitis B vaccine and its schedule	Yes	No	Unclear
	Fully described for HBIG and its schedule	Yes	No	Unclear
	Fully described for peripartum antiviral prophylaxis and its timing	Yes	No	Unclear

Supplementary Results 1. List of articles with overlapping study population

Articles used for the meta-analysis (n = 29)	Articles not used for the meta-analysis (n = 41)	Total number of articles (N=70)
Cheung K et al, 2018	Cheung K et al, 2019 & Cheung K et al, 2019	3
Elefsiniotis I et al, 2007	Elefsiniotis I et al, 2005	2
Elefsiniotis I et al, 2010	Elefsiniotis I et al, 2011	2
Foaud H et al, 2019	Foaud H et al, 2015	2
Giles M et al, 2015	Giles M et al, 2013	2
Guingané A et al, 2022	Guingané A et al, 2020	2
Guo J et al, 2015	Gao Y et al, 2015	2
Guo Z et al, 2013	Wei J et al, 2015	2
Ilboudo D et al, 2010	Yelemkoure E et al, 2018	2
Kang W et al, 2014	Kang W et al, 2017	2
Lee L et al, 2015	Lee L et al, 2019	2
Li X et al, 2003	Li X et al, 2004	2
Lin X et al, 2014	Wan Z et al, 2017	2
Liu J et al, 2018	Chen T et al, 2013 & Liu J et al, 2015 & Liu J et al, 2017	4

Liu Z et al, 2019	Yin X et al, 2020	2
Lu Y et al, 2017	Lu Y et al, 2021 & Lu Y et al, 2016 & Sun K X et al, 2012	4
Pan C et al, 2013	Zou H et al, 2011 & Zou H et al, 2012	3
Pande C et al, 2013	Pande C et al, 2013 & Pande C et al, 2011	3
Peng S et al, 2018	Peng S et al, 2019 & Peng S et al, 2018	3
Segeral O et al, 2022	Segeral O et al, 2020	2
Shao Z et al, 2011	Shao Z et al, 2007	2
Shimakawa Y et al, 2022	Ducancelle A et al, 2013	2
Su H et al, 2011	Su H et al, 2005	2
Wang C et al, 2016	Cao M et al, 2018 & Du Y et al, 2017	3
Xu C et al, 2018	Liu J et al, 2015 & Liu Y et al, 2014	3
Yi W et al, 2018	Yi W et al, 2014	2
Yin Y et al, 2012	Yin Y et al, 2013	2
Patel N et al, 2019	Joshi S et al, 2017 & Joshi S et al, 2020	3
Zhang L et al, 2014	Zhang L et al, 2014 & Zhang L et al, 2016	3

Supplementary Results 2. Study characteristics

Supplementary Results 2A. Characteristics of the included studies

131 studies reported in 172 articles

WHO region	General characteristics						Pregnant women		
	Author, year (* indicates the presence of other articles with overlapping study population)	Country	Design	Year	Recruitment at primary care facilities	Review question	Age (years)	HBeAg assay	No. included in Q1/Q2/Q3
AFR	Aba H et al, 2016	Nigeria	Cross Sectional	2011	N	Q2	NR	RDT	NA/31/NA
	Anaedobe C et al, 2015	Nigeria	Cross Sectional	2013	N	Q2	Mean 32 ± 4.8	EIA	NA/15/NA
	Andersson M et al, 2013	South Africa	Retrospective	2008	N	Q2	NR	EIA	NA/94/NA
	Andreotti M et al, 2014	Malawi	Prospective	2008-2009	N	Q2	NR	CLIA	NA/27/NA
	Bhattacharya D et al, 2021	Sub-saharan africa (South Africa, Tanzania, Uganda, and Zimbabwe)	Prospective	2007-2010	Y	Q1Q2	NR	EIA	88/88/NA

	Candotti D et al, 2007	Ghana	Cross Sectional	NR	N	Q1	NR	NA	199/NA/NA
	Chakvetadze C et al, 2011	Mayotte	Retrospective	1994-2007	N	Q1Q2	Median 23.5 (IQR 20-29)	NR	57/93/NA
	Chasela C et al, 2014	Malawi	Prospective	2007	N	Q2	Median 25 (IQR 22-29)	EIA	NA/102/NA
	Frempong M et al, 2019	Ghana	Cross Sectional	2012-2013	Y	Q2	NR	RDT	NA/32/NA
	Geffert K et al, 2020	Tanzania	Cross Sectional	2014-2015	N	Q1Q2Q3	Mean 27.45 ± 5.4	EIA	22/22/22
	Guingané A et al, 2022*	Burkina Faso	Prospective	2014-2019	Y	Q1Q2Q3	NR	RDT	623/689/578
	Kfutwah A et al, 2012	Cameroon	Cross Sectional	2000-2003	N	Q2	NR	EIA	NA/51/NA
	Loarec A et al, 2022	Mozambique	Prospective	2017-2019	N	Q1Q2Q3	Median 29.1 (IQR 23.5–33.1)	FIA	267/265/252
	Matthews P et al, 2015	South Africe and Botswana	Retrospective	2004-2013	Y	Q2	NR	CLIA	NA/66/NA
	Rouet F et al, 2004	Côte d'Ivoire	Retrospective	1995-2002	N	Q2	Median 23	EIA	NA/85/NA
	Shimakawa Y et al, 2022*	Cameroon	Prospective	2009-2016	Y	Q1Q2Q3Q4	Median 24 (IQR 20-30)	EIA	594/597/594

	Thumbiran N et al, 2014	South Africa	Cross Sectional	2009	Y	Q2	NR	EIA	NA/30/NA
AMR	Biondi M et al, 2020	Canada	Retrospective	2012-2016	Y	Q1Q2	NR	NR	1556/541/NA
	Bzowej N et al, 2019	USA	Prospective	2011-2016	N	Q1Q2	Median 33 (range 18-51)	NR	156/151/NA
	Izquierdo G et al, 2019	Chile	Prospective	2017-2018	N	Q1Q2Q3	Median 28 (IQR 25-32)	NR	30/30/30
	Kubo A et al, 2014	USA	Retrospective	2007-2010	Y	Q1Q2Q3Q4	NR	NR	835/835/835
	Lyu J et al, 2019	USA	Retrospective	2007-2017	N	Q1Q2Q3	Mean 29.1 ± 4.7	NR	1241/1241/1241
	Nguyen G et al, 2009	USA	Retrospective	2000-2008	N	Q1Q2Q3	Mean 30.3 ± 4.6	NR	27/29/27
	Patel N, 2019*	Canada	Prospective	NR	N	Q1Q2Q3	NR	CLIA	41/41/41
	Rajbhandari R et al, 2016	USA	Retrospective	1995-2013	N	Q2	NR	NR	NA/202/NA
	Tohme R et al, 2016	Haïti	Cross Sectional	2012	Y	Q1	NR	NA	33/NA/NA
	Van Ommen C et al, 2019	Canada	Prospective	2011-2015	Y	Q1Q2Q3	Median 32	NR	59/59/59

	Van Schalkwyk J et al, 2014	Canada	Retrospective	2008-2010	Y	Q2	NR	NR	NA/574/NA
EMR	Ahmadinejad Z et al, 2016	Iran	Retrospective	2008-2013	N	Q2	Mean 30.4 ± 6.0	NR	NA/30/NA
	El-Karaksy H et al, 2014	Egypt	Prospective	2010-2011	N	Q1Q2Q3	Mean 27.1 ± 4.8	NR	35/35/35
	Foaud H et al, 2019*	Egypt	Prospective	2012-2015	N	Q1Q2Q3Q4	NR	EIA	48/48/48
	Hannachi N et al, 2009	Tunisia	Cross Sectional	2007	Y	Q2	NR	EIA	55/92/NA
	Hannachi N et al, 2010	Tunisia	Cross Sectional	2007-2008	Y	Q2	NR	EIA	NA/105/NA
	Kishk R et al, 2020	Egypt	Cross Sectional	2018-2019	N	Q1Q2Q3	Mean 27.76 ± 6.77	EIA	30/30/30
	Makhlouf N et al, 2014	Egypt	Prospective	NR	N	Q1Q2Q3Q4	Mean 25.38 ± 5.46	CLIA	14/14/14
	Sbiti M et al, 2016	Morocco	Cross Sectional	2014-2015	Y	Q2	NR	CLIA	NA/24/NA
	Zahran K et al, 2010	Egypt	Cross Sectional	2008-2009	N	Q2	NR	EIA	NA/25/NA
EUR	Belopolskaya M et al, 2015	Russia	Prospective	2011-2013	NR	Q1Q2	Mean 29 ± 1.44	CLIA	31/31/NA

	Bhattacharya S et al, 2008	United Kingdom	Retrospective	2003-2006	Y	Q1Q2Q3	Median 29 (range 18–51)	EIA	112/112/112
	Denis F et al, 2004	France	Retrospective	1984-1998	N	Q2	NR	CLIA	NA/146/NA
	Dervisevic S et al, 2007	United Kingdom	Cross Sectional	1989-2004	N	Q2	NR	NR	NA/114/NA
	Dyson J et al, 2014	United Kingdom	Retrospective	2007-2011	N	Q1Q2	Median 28 (range 18–40)	NR	67/81/NA
	Eilard A et al, 2019	Sweden	Prospective	2009-2012	N	Q1Q2Q3	NR	CLIA	42/42/42
	Elefsiniotis I et al, 2007*	Greece	Cross Sectional	2003-2005	N	Q1Q2Q3	NR	EIA	63/411/63
	Elefsiniotis I et al, 2010	Greece	Prospective	2008-2009	N	Q2	NR	NR	NA/70/NA
	Godbole G et al, 2013	United Kingdom	Retrospective	2009-2010	N	Q1Q2Q3	Median 29 (range 15-46)	NR	293/401/293
	Harder K et al, 2011	Denmark	Prospective	2005-2007	Y	Q2	NR	NR	NA/342/NA
	Keel P et al, 2016	United Kingdom	Retrospective	2009	Y	Q2	NR	NR	NA/361/NA
	Papaevangelou V et al, 2011	Greece	Prospective	2004-2007	N	Q1Q2Q3Q4	NR	EIA	109/109/109

	Ruiz-Extremera Á et al, 2020	Spain	Prospective	2015	N	Q1Q2Q3Q4	NR	EIA	62/62/62
	Safadi R et al, 2021	Israel	Prospective	2009-2014	N	Q1Q2	NR	CLIA	171/171/NA
	Sagnelli E et al, 2016	Italy	Cross Sectional	2012-2013	N	Q2	Mean 31 ± 12.1	EIA	NA/143/NA
	Schulpis K et al, 2008	Greece	Cross Sectional	NR	N	Q1Q2Q3	Mean 28 ± 3.6	EIA	28/28/28
	Sellier P et al, 2015	France	Retrospective	2004-2012	N	Q1	NR	NA	417/NA/NA
	Sellier P et al, 2018	France	Retrospective	2004-2015	N	Q1Q2Q3	Mean 29 ± 6.3	CLIA	16/16/16
	Söderström A et al, 2003	Sweden	Retrospective	1998-2000	N	Q2	NR	EIA	NA/264/NA
	Ter Borg M et al, 2008	Netherlands	Retrospective	1998-2006	N	Q2	Median 25.6 (range 18.2–40.5)	EIA	NA/38/NA
	White H et al, 2015	United Kingdom	Retrospective	2005-2011	Y	Q1Q2Q3	Median 30 (range 17-45)	NR	123/172/123
**SEAR**	Banerjee A et al, 2005	India	Cross Sectional	1998	Y	Q2	Mean 21.3 ± 3.1	EIA	NA/15/NA
	Dachlan E et al, 2020	Indonesia	Cross Sectional	2016	N	Q2	Mean 31.42 ± 6.1	CLIA	NA/33/NA

	Dwivedi M et al, 2011	India	Prospective	2006-2007	N	Q2	NR	EIA	NA/37/NA
	Fujiko M et al, 2015	Indonesia	Cross Sectional	2014-2014	Y	Q1Q2	Median 29 (range 18–42)	EIA	64/64/NA
	Pande C et al, 2013*	India	Prospective	2004-2009	N	Q2	Median 24 (range 19-35)	EIA	NA/259/NA
	Sirilert S et al, 2019	Thaïland	Prospective	2017-2019	N	Q2	NR	EIA	NA/87/NA
	Wibowo PW et al, 2020	Indonesia	Cross Sectional	2017-2018	N	Q2	NR	FIA	NA/52/NA
**WPR**	Bergin H et al, 2017	Australia	Retrospective	2014-2015	N	Q1Q2Q3	NR	NR	99/87/87
	Chen HL et al, 2012	Taiwan	Retrospective	1996-2008	N	Q2	NR	NR	NA/2356/NA
	Chen J et al, 2014	China	Cross Sectional	2009-2011	NR	Q2	Mean 28.0 ± 5.7	CLIA	NA/334/NA
	Chen T et al, 2018	China	Cross Sectional	2010-2015	Y	Q1Q2	Median 27 (range 19–42)	CLIA	951/951/NA
	Chen X et al, 2013	China	Retrospective	2002-2010	N	Q2	Mean 31.0 ± 3.5	EIA	NA/544/NA
	Chen Y et al, 2013	China	Prospective	2009-2011	N	Q1Q2	NR	FIA	171/171/NA

	Chen Z et al, 2017	China	Prospective	2011-2015	N	Q1	Median 27 (range 18-42)	NA	338/NA/NA
	Cheung K et al, 2018*	Hong Kong	Prospective	2014-2016	N	Q1Q2	NR	NR	641/641/NA
	Ding Y et al, 2013	China	Prospective	2010-2011	N	Q2	NR	CLIA	NA/249/NA
	Evans A et al, 2015	China	Prospective	2011-2012	Y	Q1Q2	NR	EIA	185/185/NA
	Giles M et al, 2015	Australia	Prospective	2009-2011	N	Q2	Mean 31.45 ± 0.43	CLIA	NA/126/NA
	Guo J et al, 2015*	China	Prospective	2011-2013	NR	Q2	NR	CLIA	NA/144/NA
	Guo Z et al, 2013*	China	Retrospective	2003-2009	N	Q1Q2	NR	EIA	1046/1043/NA
	Hu Y et al, 2016	China	Retrospective	2002-2004	Y	Q1Q2Q3	Mean 24.95 ± 3.3	CLIA	264/264/264
	Huang H et al, 2020	China	Prospective	2014-2018	N	Q2	NR	CLIA	973/973/NA
	Hui P et al, 2020	Hong Kong	Retrospective	2017-2019	N	Q1	NR	NA	265/NA/NA
	Kang W et al, 2014*	China	Prospective	2011	Y	Q1Q2	NR	EIA	2765/2765/NA

	Khue P et al, 2020	Vietnam	Prospective	2017-2018	N	Q1Q2Q3	Median 30 (IQR 26-33)	EIA	183/183/183
	Kim J et al, 2014	South Korea	Retrospective	2002-2012	N	Q2	NR	NR	NA/159/NA
	Komatsu H et al, 2016	Japan	Prospective	2007-2014	N	Q2	Median 32 (range 21–39)	EIA	NA/31/NA
	Lao T et al, 2015	Hong Kong	Prospective	2009-2012	N	Q2	Mean 32.1 ± 4.3	NR	NA/235/NA
	Latthaphasavang V et al, 2019	Laos	Prospective	2015-2017	N	Q1Q2Q3Q4	Median 28 (IQR 24-30)	EIA	153/153/153
	Lee L et al, 2015*	Singapore	Prospective	2009-2013	N	Q1Q2Q3Q4	Mean 32.4 ± 4.7	CLIA	161/161/154
	Li F et al, 2012	China	Prospective	2008-2010	N	Q1Q2	Mean 27.31 ± 3.74	EIA	221/221/NA
	Li L et al, 2020	China	Retrospective	2017-2017	N	Q1Q2Q3	Mean 29.3 ± 4.2	CLIA	317/317/317
	Li X et al, 2003*	China	Prospective	1999-2001	N	Q2	NR	EIA	NA/151/NA
	Li Y et al, 2020	China	Prospective	2011-2017	N	Q1Q2Q3Q4	NR	NR	232/232/232
	Li Z et al, 2014	China	Prospective	2009-2013	N	Q1Q2	NR	EIA	537/537/NA

	Lin X et al, 2014*	China	Prospective	2008-2012	N	Q2	NR	EIA	NA/294/NA
	Liu C et al, 2015	China	Retrospective	2010-2013	N	Q2	NR	EIA	NA/256/NA
	Liu J et al, 2018*	China	Prospective	2010-2015	Y	Q2	Median 28 (range 19-46)	CLIA	NA/1097/NA
	Liu Z et al, 2019*	China	Prospective	2015-2018	Y	Q1Q2Q3	Mean 28.2 ± 4.2	CLIA	904/893/893
	Lu L et al, 2014	China	Prospective	2010-2013	N	Q1Q2	Range 21-40	CLIA	140/140/NA
	Lu Y et al, 2017*	China	Prospective	2009-2011	Y	Q1Q2Q3	Median 26.0 (range 15.1–43.0)	CLIA	1177/1177/1177
	Lv N et al, 2014	China	Retrospective	2011-2012	N	Q2	NR	EIA	NA/42/NA
	Michitaka K et al, 2012	Japan	Prospective	2010-2010	N	Q1Q2Q3	Median 34 (range 25-40)	CLIA	21/21/21
	Nishimura K et al, 2021	Japan	Prospective	2008-2017	N	Q1Q2Q3	NR	CLIA	87/205/87
	Pan C et al, 2013*	China	Retrospective	2007-2011	N	Q1Q2	NR	CLIA	1401/1401/NA
	Peng S et al, 2018*	China	Prospective	2012-2017	N	Q2	NR	EIA	1234/1345/NA

	Peng T et al, 2019	China	Prospective	2011-2016	N	Q1	NR	NA	750/NA/NA
	Qiao Y et al, 2019	China	Cross Sectional	2017-2018	Y	Q2	Mean 31 ± 5	NR	NA/4112/NA
	Sasagawa Y et al, 2019	Japan	Prospective	2008-2016	N	Q1Q2Q3Q4	Median 32 (range 23-44)	NR	35/35/35
	Ségéral O et al, 2018	Cambodia	Prospective	2015	N	Q1Q2Q3	NR	RDT	128/128/128
	Segeral O et al, 2022*	Cambodia	Prospective	2017-2020	Y	Q1Q2Q3Q4	Median 29 (IQR 26-33)	RDT	1194/1194/1194
	Shao Z et al, 2011*	China	Prospective	2002-2005	N	Q2	NR	EIA	NA/212/NA
	Sheng Q et al, 2018	China	Prospective	2016	N	Q2	NR	CLIA	NA/441/NA
	Shi X et al, 2017	China		2001-2002	N	Q2	NR	EIA	NA/150/NA
	Su H et al, 2011*	China	Cross Sectional	1997-2002	N	Q2	NR	EIA	NA/457/NA
	Thilakanathan C et al, 2018	Australia	Retrospective	2008-2015	N	Q1Q2Q3	Median 30 (range 15-44)	NR	642/642/642
	Wang C et al, 2016*	China	Prospective	2012-2015	Y	Q2	NR	CLIA	NA/890/NA

	Wang D et al, 2019	China	Prospective	2011-2013	N	Q2	NR	CLIA	NA/290/NA
	Wang J et al, 2005	China	Prospective	2000-2001	N	Q2	NR	EIA	NA/42/NA
	Wang L et al, 2016	China	Prospective	2012-2015	N	Q1Q2Q3	NR	FIA	31/31/31
	Wang Z et al, 2003	China	Prospective	2000-2001	N	Q2	NR	EIA	NA/54/NA
	Wiseman E et al, 2009	Australia	Prospective	2002-2008	N	Q2	NR	EIA/CLIA	NA/313/NA
	Wu K et al, 2020	China	Retrospective	2018	N	Q2	Mean 32.17 ± 4.46	NR	NA/1129/NA
	Xu C et al, 2018*	China	Cross Sectional	2009-2014	N	Q1Q2Q3	Median 25.3 (IQR 22.7–30.2)	CLIA	214/214/214
	Xu D et al, 2002	China	Retrospective	1993-1997	N	Q2	NR	EIA	NA/402/NA
	Xu Y et al, 2015	China	Retrospective	2008-2012	Y	Q1Q2	Range 20-40	EIA	312/312/NA
	Yi W et al, 2018*	China	Retrospective	2008-2015	N	Q2	Mean 29.15 ± 4.35	CLIA	NA/3367/NA
	Yin Y et al, 2012*	China	Prospective	2006-2010	N	Q2	Mean 28.8 ± 3.9	EIA	NA/1355/NA

	Yonghao G et al, 2017	China	Cross Sectional	2013-2014	Y	Q2	Mean 27.2 ± 4.6	EIA	NA/336/NA
	Zhang L et al, 2014	China	Prospective	2008-2012	Y	Q1Q2	NR	EIA	630/1186/NA
	Zhang Z et al, 2014	China	Prospective	2004-2005	N	Q1Q2	NR	EIA	174/174/NA
	Zhou Y et al, 2017	China	Retrospective	2010-2012	Y	Q2	NR	EIA	NA/20827/NA
	Zhu Y et al, 2010	China	Prospective	2006-2008	N	Q2	Mean 34.02 ± 5.63	EIA	NA/252/NA

Abbreviations: CLIA, chemiluminescent immunoassay; EIA, enzyme immunoassay; FIA, fluorescent immunoassay; HBeAg, hepatitis B e antigen; NA, not available; NR, not reported; RDT, rapid diagnostic test;

Supplementary Results 2B. Characteristics of the included cohorts in Q4

20 intervention arms from 11 cohorts (11 studies)

WHO region	General characteristics					Pregnant women		Infants		
	Author, year	Country	Design	Year	Recruitment at primary care facilities	Age (years)	HBeAg assay	Age at testing (months)	PMTCT intervention	N
AFR	Shimakawa Y et al, 2022*	Cameroon	Prospective	2009-2016	Y	Median 24 (IQR 20-30)	EIA	6-24	HepB-BD/HepB3	176
AMR	Kubo A et al, 2014	USA	Retrospective	2007-2010	Y	NR	NR	9-15	HepB-BD/HepB2/HBIG/PAP**	127
									HepB-BD/HepB2/HBIG**	708
EMR	Foaud H et al, 2019*	Egypt	Prospective	2012-2015	N	NR	EIA	≥ 6	HepB-BD/HepB3/HBIG/PAP	9
									HepB-BD/HepB3/HBIG	39
	Makhlouf N et al, 2014	Egypt	Prospective	NR	N	Mean 25.38 ± 5.46	CLIA	9	HepB-BD/HepB2/HBIG	14
EUR	Papaevangelou V et al, 2011	Greece	Prospective	2004-2007	N	NR	EIA	9	HepB-BD/HepB2/HBIG	109
	Ruiz-Extremera Á et al, 2020	Spain	Prospective	2015	N	NR	EIA	18	HepB-BD/HepB/HBIG/PAP	2

									HepB-BD/HepB/HBIG	52
WPR	Latthaphasavang V et al, 2019	Laos	Prospective	2015-2017	N	Median 28 (IQR 24-30)	EIA	6	HepB-BD/HepB3**	119
	Lee L et al, 2015*	Singapore	Prospective	2009-2013	N	Mean 32.4 ± 4.7	CLIA	9	HepB-BD/HepB2/HBIG/	154
	Li Y et al, 2020	China	Prospective	2011-2017	N	NR	NR	6	HepB	6
									HepB-BD/HepB/HBIG	135
									HepB-BD/HepB/HBIG/PAP	108
									HepB/PAP	2
	Segeral O et al, 2022*	Cambodia	Prospective	2017-2020	Y	Median 29 (IQR 26-33)	RDT	6	HepB-BD/HepB3/HBIG/PAP	37
									HepB-BD/HepB3/HBIG	109
									HepB-BD/HepB3/PAP	263
									HepB-BD/HepB3	575

Abbreviations: CLIA, chemiluminescent immunoassay; EIA, enzyme immunoassay; HBeAg, hepatitis B e antigen; HBIG, hepatitis B immune globulin; HepB; infant hepatitis B vaccine; HepB-BD, hepatitis B birth dose vaccine; NR, not reported; PAP, peripartum antiviral prophylaxis; RDT, rapid diagnostic test.

* Indicates the presence of other articles with overlapping study population.

** Type of PMTCT interventions was determined based on the standard of care described in each article, regardless of whether participants complied with these recommendations.

Supplementary Results 3. Risk of bias

Supplementary Results 3A. Risk of bias of the studies included in Q1, Q2, and Q3

WHO region	Author, year (* indicates the presence of other articles with overlapping study population)	Country	Review question	Was the study's target population a close representation of the national population in relation to relevant variables?	Was the sampling frame a true or close representation of the target population?	Was some form of systematic, random selection used to select the sample, or was a census undertaken?	Was the likelihood of selecting women under concomitant antiviral treatment at baseline evaluation minimal?	Was the likelihood of non-response bias minimal for HBeAg test (75%)?	Was the likelihood of non-response bias minimal for HBV DNA test (75%)?	Was the study instrument that measured HBeAG status shown to have validity and reliability?
AFR	Bhattacharya D et al, 2021	Sub-saharan africa (South Africa, Tanzania, Uganda, and Zimbabwe)	Q1Q2	Low risk	Low risk	High risk	High risk	Low risk	Low risk	Low risk
	Andersson M et al, 2013	South Africa	Q2	High risk	High risk	High risk	High risk	Low risk	NA	Low risk
	Andreotti M et al, 2014	Malawi	Q2	High risk	High risk	High risk	Low risk	Low risk	NA	Low risk
	Kfutwah A et al, 2012	Cameroon	Q2	High risk	High risk	High risk	High risk	Unknown risk	NA	Low risk
	Frempong M et al, 2019	Ghana	Q2	High risk	Low risk	High risk	High risk	Unknown risk	NA	High risk
	Chakvetadze C et al, 2011	Mayotte	Q1Q2	High risk	High risk	High risk	High risk	Unknown risk	Unknown risk	Unknown risk
	Shimakawa Y et al, 2022*	Cameroon	Q1Q2Q3Q4	High risk	Low risk	Low risk	High risk	High risk	High risk	Low risk
	Geffert K et al, 2020	Tanzania	Q1Q2Q3	High risk	High risk	Low risk	High risk	Low risk	Low risk	Low risk

	Loarec A et al, 2022	Mozambique	Q1Q2Q3	High risk	High risk	Low risk	High risk	Low risk	Low risk	Low risk
	Candotti D et al, 2007	Ghana	Q1	High risk	High risk	Low risk	Low risk	NA	Low risk	NA
	Aba H et al, 2016	Nigeria	Q2	High risk	High risk	Low risk	High risk	Low risk	NA	High risk
	Anaedobe C et al, 2015	Nigeria	Q2	High risk	High risk	Low risk	Low risk	Low risk	NA	Low risk
	Chasela C et al, 2014	Malawi	Q2	High risk	High risk	Low risk	High risk	Low risk	NA	Low risk
	Rouet F et al, 2004	Côte d'Ivoire	Q2	High risk	High risk	Low risk	Low risk	Low risk	NA	Low risk
	Matthews P et al, 2015	South Africe and Botswana	Q2	High risk	Low risk	Low risk	High risk	Low risk	NA	Low risk
	Thumbiran N et al, 2014	South_Africa	Q2	High risk	Low risk	Low risk	High risk	Low risk	NA	Low risk
	Guingané A et al, 2022*	Burkina Faso	Q1Q2Q3	High risk	Low risk	Low risk	High risk	Unknown risk	Unknown risk	High risk
**AMR**	Nguyen G et al, 2009	USA	Q1Q2Q3	High risk	High risk	High risk	High risk	High risk	High risk	Unknown risk
	Lyu J et al, 2019	USA	Q1Q2Q3	High risk	High risk	High risk	Low risk	Low risk	Low risk	Low risk
	Patel N et al, 2019*	Canada	Q1Q2Q3	High risk	High risk	High risk	Low risk	Unknown risk	Unknown risk	Low risk
	Biondi M et al, 2020	Canada	Q1Q2	High risk	Low risk	Low risk	High risk	High risk	High risk	Unknown risk
	Kubo A et al, 2014	USA	Q1Q2Q3Q4	High risk	Low risk	Low risk	High risk	High risk	High risk	Unknown risk
	Van Ommen C et al, 2019	Canada	Q1Q2Q3	High risk	Low risk	Low risk	Low risk	High risk	High risk	Unknown risk
	Bzowej N et al, 2019	USA	Q1Q2	High risk	High risk	Low risk	Low risk	Low risk	Low risk	Unknown risk
	Izquierdo G et al, 2019	Chile	Q1Q2Q3	High risk	High risk	Low risk	High risk	Low risk	Low risk	Unknown risk
	Tohme R et al, 2016	Haïti	Q1	Low risk	Low risk	Low risk	High risk	NA	Low risk	NA
	Rajbhandari R et al, 2016	USA	Q2	High risk	High risk	Low risk	High risk	High risk	NA	Unknown risk
	Van Schalkwyk J et al, 2014	Canada	Q2	High risk	Low risk	Low risk	High risk	Unknown risk	NA	Unknown risk
**EMR**	El-Karaksy H et al, 2014	Egypt	Q1Q2Q3	High risk	High risk	High risk	High risk	High risk	High risk	Unknown risk

	Kishk R et al, 2020	Egypt	Q1Q2Q3	High risk	High risk	High risk	High risk	Low risk	Low risk	Low risk
	Ahmadinejad Z et al, 2016	Iran	Q2	High risk	High risk	High risk	High risk	High risk	NA	Unknown risk
	Makhlouf N et al, 2014	Egypt	Q1Q2Q3Q4	High risk	High risk	Low risk	High risk	Low risk	Low risk	Low risk
	Zahran K et al, 2010	Egypt	Q2	High risk	High risk	Low risk	High risk	Low risk	NA	Low risk
	Hannachi N et al, 2009	Tunisia	Q2	High risk	Low risk	Low risk	Low risk	Low risk	NA	Low risk
	Hannachi N et al, 2010	Tunisia	Q2	High risk	Low risk	Low risk	High risk	Low risk	NA	Low risk
	Sbiti M et al, 2016	Morocco	Q2	High risk	Low risk	Low risk	Low risk	Low risk	NA	Low risk
	Foaud H et al, 2019*	Egypt	Q1Q2Q3Q4	High risk	High risk	Low risk	Low risk	Unknown risk	Unknown risk	Low risk
**EUR**	Sellier P et al, 2018	France	Q1Q2Q3	High risk	High risk	High risk	High risk	High risk	High risk	Low risk
	Sagnelli E et al, 2016	Italy	Q2	High risk	High risk	High risk	High risk	Unknown risk	NA	Low risk
	Söderström A et al, 2003	Sweden	Q2	High risk	High risk	High risk	High risk	Unknown risk	NA	Low risk
	Keel P et al, 2016	United Kingdom	Q2	High risk	Low risk	High risk	High risk	Unknown risk	NA	Unknown risk
	Schulpis K et al, 2008	Greece	Q1Q2Q3	High risk	High risk	High risk	High risk	Unknown risk	Unknown risk	Low risk
	Belopolskaya M et al, 2015	Russia	Q1Q2	High risk	Unknown risk	High risk	Low risk	Unknown risk	Unknown risk	Low risk
	Eilard A et al, 2019	Sweden	Q1Q2Q3	High risk	High risk	Low risk	High risk	High risk	High risk	Low risk
	Ruiz-Extremera Á et al, 2020	Spain	Q1Q2Q3Q4	High risk	High risk	Low risk	High risk	High risk	High risk	Low risk
	Bhattacharya S et al, 2008	United Kingdom	Q1Q2Q3	High risk	Low risk	Low risk	High risk	High risk	High risk	Low risk
	Elefsiniotis I et al, 2007*	Greece	Q1Q2Q3	High risk	High risk	Low risk	High risk	Low risk	High risk	Low risk
	Godbole G et al, 2013	United Kingdom	Q1Q2Q3	High risk	High risk	Low risk	High risk	Low risk	High risk	Unknown risk
	Sellier P et al, 2015	France	Q1	High risk	High risk	Low risk	Low risk	NA	Low risk	NA
	Denis F et al, 2004	France	Q2	High risk	High risk	Low risk	High risk	Low risk	NA	Low risk

	Elefsiniotis I et al, 2010	Greece	Q2	High risk	High risk	Low risk	High risk	Low risk	NA	Unknown risk
	Harder K et al, 2011	Denmark	Q2	Low risk	Low risk	Low risk	High risk	Low risk	NA	Unknown risk
	Dervisevic S et al, 2007	United Kingdom	Q2	High risk	High risk	Low risk	High risk	Unknown risk	NA	Unknown risk
	Dyson J et al, 2014	United Kingdom	Q1Q2	High risk	High risk	Low risk	High risk	Unknown risk	Unknown risk	Unknown risk
	Papaevangelou V et al, 2011	Greece	Q1Q2Q3Q4	High risk	High risk	Low risk	Low risk	Unknown risk	Unknown risk	Low risk
	Safadi R et al, 2021	Israel	Q1Q2	High risk	High risk	Low risk	Low risk	Unknown risk	Unknown risk	Low risk
	White H et al, 2015	United Kingdom	Q1Q2Q3	High risk	Low risk	Low risk	High risk	Unknown risk	Unknown risk	Unknown risk
	Ter Borg M et al, 2008	Netherlands	Q2	High risk	High risk	Unknown risk	Low risk	Unknown risk	NA	Low risk
**SEAR**	Dachlan E et al, 2020	Indonesia	Q2	High risk	High risk	High risk	Low risk	Unknown risk	NA	Low risk
	Dwivedi M et al, 2011	India	Q2	High risk	High risk	Low risk	High risk	Low risk	NA	Low risk
	Pande C et al, 2013*	India	Q2	High risk	High risk	Low risk	Low risk	Low risk	NA	Low risk
	Banerjee A et al, 2005	India	Q2	High risk	Low risk	Low risk	High risk	Low risk	NA	Low risk
	Sirilert S et al, 2019	Thaïland	Q2	High risk	High risk	Low risk	High risk	Unknown risk	NA	Low risk
	Fujiko M et al, 2015	Indonesia	Q1Q2	High risk	Low risk	Low risk	Low risk	Unknown risk	Unknown risk	Low risk
	Wibowo PW et al, 2020	Indonesia	Q2	High risk	High risk	Unknown risk	High risk	Unknown risk	NA	Low risk
**WPR**	Pan C et al, 2013*	China	Q1Q2	High risk	High risk	High risk	Low risk	High risk	High risk	Low risk
	Wang L et al, 2016	China	Q1Q2Q3	High risk	High risk	High risk	Low risk	High risk	High risk	Low risk
	Zhang L et al, 2014	China	Q1Q2	High risk	Low risk	High risk	High risk	High risk	High risk	Low risk
	Zhang Z et al, 2014	China	Q1Q2	High risk	High risk	High risk	Low risk	Low risk	Low risk	Low risk
	Liu Z et al, 2019*	China	Q1Q2Q3	High risk	Low risk	High risk	Low risk	Low risk	Low risk	Unknown risk
	Liu C et al, 2015	China	Q2	High risk	High risk	High risk	High risk	High risk	NA	Low risk

	Yin Y et al, 2012*	China	Q2	High risk	High risk	High risk	Low risk	Low risk	NA	Low risk
	Zhu Y et al, 2010	China	Q2	High risk	High risk	High risk	High risk	Low risk	NA	Low risk
	Zhou Y et al, 2017	China	Q2	Low risk	Low risk	High risk	High risk	Low risk	NA	Low risk
	Lv N et al, 2014	China	Q2	High risk	High risk	High risk	Low risk	Unknown risk	NA	Low risk
	Shao Z et al, 2011*	China	Q2	High risk	High risk	High risk	High risk	Unknown risk	NA	Low risk
	Chen J et al, 2014	China	Q2	High risk	Unknown risk	High risk	Low risk	Unknown risk	NA	Low risk
	Guo Z et al, 2013*	China	Q1Q2	High risk	High risk	Low risk	High risk	High risk	High risk	Low risk
	Li F et al, 2012	China	Q1Q2	High risk	High risk	Low risk	High risk	High risk	High risk	Low risk
	Li L et al, 2020	China	Q1Q2Q3	High risk	High risk	Low risk	Low risk	High risk	High risk	Low risk
	Li Z et al, 2014	China	Q1Q2	High risk	High risk	Low risk	High risk	High risk	High risk	Low risk
	Evans A et al, 2015	China	Q1Q2	High risk	Low risk	Low risk	High risk	High risk	High risk	Low risk
	Hu Y et al, 2016	China	Q1Q2Q3	High risk	Low risk	Low risk	Low risk	High risk	High risk	Low risk
	Nishimura K et al, 2021	Japan	Q1Q2Q3	High risk	High risk	Low risk	Low risk	Low risk	High risk	Low risk
	Chen Z et al, 2017	China	Q1	High risk	High risk	Low risk	Low risk	NA	High risk	NA
	Hui P et al, 2020	Hong_Kong	Q1	High risk	High risk	Low risk	High risk	NA	High risk	NA
	Bergin H et al, 2017	Australia	Q1Q2Q3	High risk	High risk	Low risk	High risk	Low risk	Low risk	Unknown risk
	Cheung K et al, 2018*	Hong_Kong	Q1Q2	High risk	High risk	Low risk	Low risk	Low risk	Low risk	Unknown risk
	Khue P et al, 2020	Vietnam	Q1Q2Q3	High risk	High risk	Low risk	Low risk	Low risk	Low risk	Low risk
	Latthaphasavang V et al, 2019	Laos	Q1Q2Q3Q4	High risk	High risk	Low risk	High risk	Low risk	Low risk	Low risk
	Lee L et al, 2015*	Singapore	Q1Q2Q3Q4	High risk	High risk	Low risk	High risk	Low risk	Low risk	Unknown risk
	Michitaka K et al, 2012	Japan	Q1Q2Q3	High risk	High risk	Low risk	High risk	Low risk	Low risk	Low risk
	Ségéral O et al, 2018	Cambodia	Q1Q2Q3	High risk	High risk	Low risk	High risk	Low risk	Low risk	High risk

	Chen T et al, 2018	China	Q1Q2	High risk	Low risk	Low risk	High risk	Low risk	Low risk	Low risk
	Lu Y et al, 2017*	China	Q1Q2Q3	High risk	Low risk	Low risk	Low risk	Low risk	Low risk	Low risk
	Segeral O et al, 2022*	Cambodia	Q1Q2Q3Q4	High risk	Low risk	Low risk	Low risk	Low risk	Low risk	High risk
	Peng T et al, 2019	China	Q1	High risk	High risk	Low risk	High risk	NA	Low risk	NA
	Chen X et al, 2013	China	Q2	High risk	High risk	Low risk	Low risk	High risk	NA	Low risk
	Kim J et al, 2014	South Korea	Q2	High risk	High risk	Low risk	Low risk	High risk	NA	Unknown risk
	Lin X et al, 2014*	China	Q2	High risk	High risk	Low risk	High risk	High risk	NA	Low risk
	Yonghao G et al, 2017	China	Q2	High risk	Low risk	Low risk	High risk	High risk	NA	Low risk
	Ding Y et al, 2013	China	Q2	High risk	High risk	Low risk	High risk	Low risk	NA	Low risk
	Huang H et al, 2020	China	Q2	High risk	High risk	Low risk	Low risk	Low risk	NA	Low risk
	Lao T et al, 2015	Hong_Kong	Q2	High risk	High risk	Low risk	Low risk	Low risk	NA	Unknown risk
	Peng S et al, 2018*	China	Q2	High risk	High risk	Low risk	High risk	Low risk	NA	Low risk
	Sasagawa Y et al, 2019	Japan	Q1Q2Q3Q4	High risk	High risk	Low risk	High risk	Low risk	NA	Unknown risk
	Sheng Q et al, 2018	China	Q2	High risk	High risk	Low risk	High risk	Low risk	NA	Low risk
	Wu K et al, 2020	China	Q2	High risk	High risk	Low risk	High risk	Low risk	NA	Unknown risk
	Yi W et al, 2018*	China	Q2	High risk	High risk	Low risk	Low risk	Low risk	NA	Low risk
	Qiao Y et al, 2019	China	Q2	Low risk	Low risk	Low risk	High risk	Low risk	NA	Unknown risk
	Wang C et al, 2016*	China	Q2	High risk	Low risk	Low risk	Low risk	Low risk	NA	Low risk
	Chen HL et al, 2012	Taiwan	Q2	High risk	High risk	Low risk	High risk	Unknown risk	NA	Unknown risk
	Giles M et al, 2015	Australia	Q2	High risk	High risk	Low risk	Low risk	Unknown risk	NA	Low risk
	Komatsu H et al, 2016	Japan	Q2	High risk	High risk	Low risk	High risk	Unknown risk	NA	Low risk
	Wang J et al, 2005	China	Q2	High risk	High risk	Low risk	High risk	Unknown risk	NA	Low risk

	Chen Y et al, 2013	China	Q1Q2	High risk	High risk	Low risk	Low risk	Unknown risk	Unknown risk	Low risk
	Thilakanathan C et al, 2018	Australia	Q1Q2Q3	High risk	High risk	Low risk	High risk	Unknown risk	Unknown risk	Unknown risk
	Kang W et al, 2014*	China	Q1Q2	High risk	Low risk	Low risk	High risk	Unknown risk	Unknown risk	Low risk
	Wang D et al, 2019	China	Q2	High risk	High risk	Unknown risk	Low risk	High risk	NA	Low risk
	Shi X et al, 2017	China	Q2	High risk	High risk	Unknown risk	High risk	Low risk	NA	Low risk
	Wiseman E et al, 2009	Australia	Q2	High risk	High risk	Unknown risk	High risk	Low risk	NA	Low risk
	Liu J et al, 2018*	China	Q2	High risk	Low risk	Unknown risk	Low risk	Low risk	NA	Low risk
	Li X et al, 2003*	China	Q2	High risk	High risk	Unknown risk	Low risk	Unknown risk	NA	Low risk
	Su H et al, 2011*	China	Q2	High risk	High risk	Unknown risk	Low risk	Unknown risk	NA	Low risk
	Wang Z et al, 2003	China	Q2	High risk	High risk	Unknown risk	High risk	Unknown risk	NA	Low risk
	Xu D et al, 2002	China	Q2	High risk	High risk	Unknown risk	High risk	Unknown risk	NA	Low risk
	Guo J et al, 2015*	China	Q2	High risk	Unknown risk	Unknown risk	Low risk	Unknown risk	NA	Low risk
	Li Y et al, 2020	China	Q1Q2Q3Q4	High risk	High risk	Unknown risk	Low risk	Unknown risk	Unknown risk	Unknown risk
	Lu L et al, 2014	China	Q1Q2	High risk	High risk	Unknown risk	High risk	Unknown risk	Unknown risk	Low risk
	Xu C et al, 2018*	China	Q1Q2Q3	High risk	High risk	Unknown risk	Low risk	Unknown risk	Unknown risk	Low risk
	Xu Y et al, 2015	China	Q1Q2	High risk	Low risk	Unknown risk	High risk	Unknown risk	Unknown risk	Low risk

Supplementary Results 3B. Risk of bias of the studies included in Q4

WHO region	Author, year (* indicates the presence of other articles with overlapping study population)	Sample selection explained (setting, locations and periods of recruitment)	Representative of review question population (i.e. recruited following positive HBsAg results at antenatal care)	Completeness (of the women HBsAg+ eligible for the study, how many were included?)	HBsAg or HBV DNA assay in infants fully defined	Proportion of infants born to enrolled HBsAg-positive mothers with HBV DNA and HbeAg assessment who were assessed for outcome	Fully described for hepatitis B vaccine and its schedule	Fully described for HBIG and its schedule	Fully described for peripartum antiviral prophylaxis and its timing
**AFR**	Shimakawa Y et al, 2022*	Low risk	Low risk	High risk	Low risk	Low risk	Low risk	Low risk	Low risk
**AMR**	Kubo A et al, 2014	Low risk	Low risk	High risk	High risk	High risk	High risk	High risk	High risk
**EMR**	Ruiz-Extremera Á et al, 2020	High risk	Low risk	Low risk	Low risk	Low risk	High risk	High risk	High risk
	Foaud H et al, 2019*	Low risk	Low risk	Low risk	Low risk	Low risk	Low risk	Low risk	Low risk
	Makhlouf N et al, 2014	Low risk	Low risk	Low risk	Low risk	Low risk	Low risk	Low risk	High risk
	Papaevangelou V et al, 2011	Low risk	Low risk	Low risk	Low risk	Low risk	Low risk	Low risk	Low risk
**WPR**	Segeral O et al, 2022*	Low risk	Low risk	Low risk	Low risk	Low risk	Low risk	Low risk	Low risk
	Li Y et al, 2020	Low risk	Low risk	Low risk	Low risk	Low risk	Low risk	High risk	Low risk
	Latthaphasavang V et al, 2019	Low risk	Low risk	Low risk	Low risk	Low risk	Low risk	Low risk	Low risk

	Lee L et al, 2015*	Low risk	Low risk	Low risk	Low risk	Low risk	Low risk	Low risk	High risk
	Sasagawa Y et al, 2019	Low risk	Low risk	Low risk	Low risk	Low risk	Low risk	Low risk	Low risk

Supplementary Results 4. Publication bias

Supplementary Results 4A. Adapted funnel plots for the studies included in Q1 (n=67)

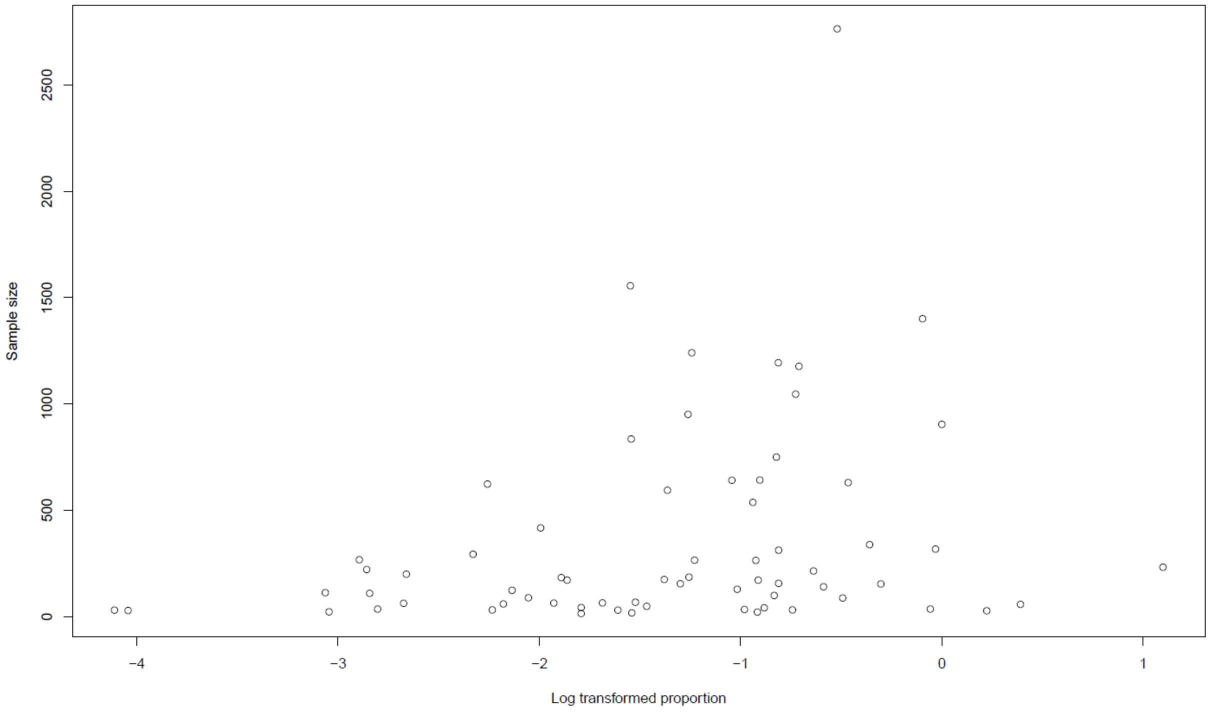

Supplementary Results 4B. Adapted funnel plots for the studies included in Q2 (n=125)

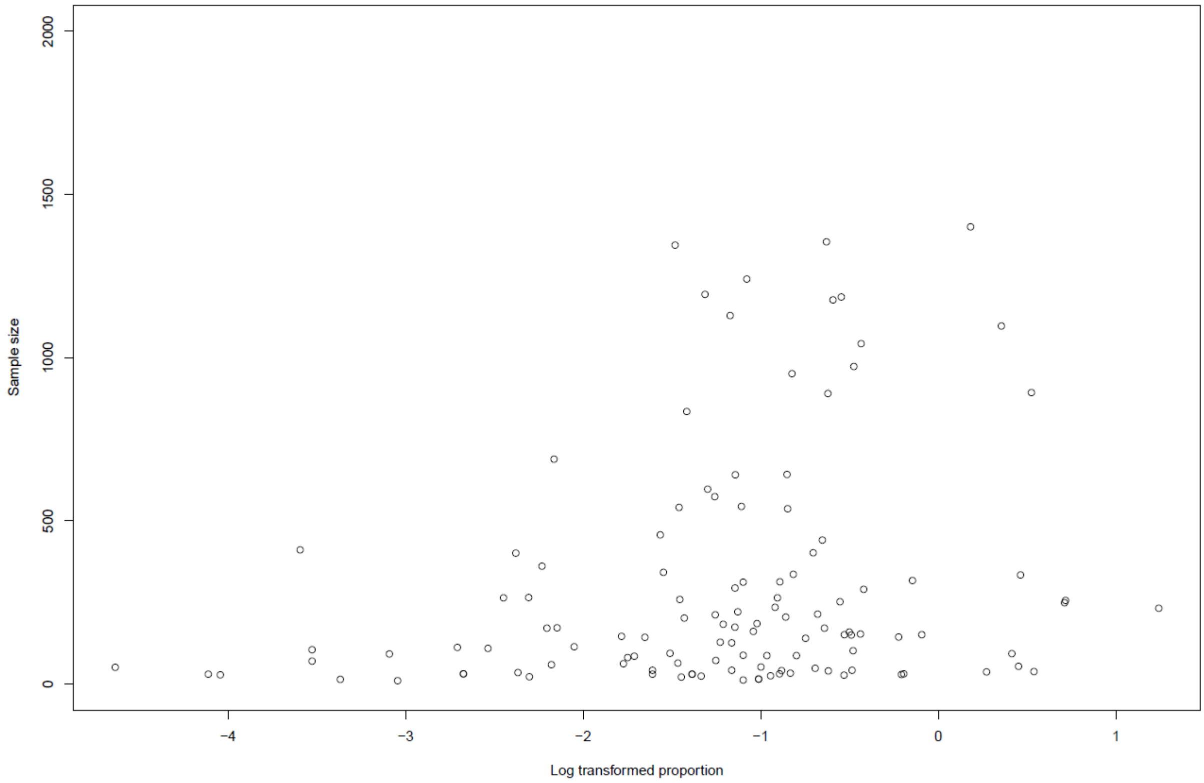

Supplementary Results 5. Description of studies that provided outlying estimates

Supplementary Results 5A. Studies showing substantial deviation towards lower estimates (n=13)

Author, year	Country	% with high viral load	% with positive HBeAg	Potential reasons
Bhattacharya S et al, 2008	UK	4.46	6.25	No apparent reason
Elefsiniotis I et al, 2007	Greece	12.70	2.68	No apparent reason
Elefsiniotis I et al, 2010	Greece	NR	2.86	No apparent reason
Frempong M et al, 2019	Ghana	NR	0	Small number of participants (N=10)
Geffert K et al, 2020	Tanzania	4.55	9.09	Small number of participants (N=22)
Hannachi N et al, 2009	Tunisia	NR	4.35	No apparent reason
Hannachi N et al, 2010	Tunisia	NR	2.86	No apparent reason
Kfutwah A et al, 2012a	Cameroon	NR	0	All women co-infected with HIV and small number of participants (N=23)
Kfutwah A et al, 2012b	Cameroon	NR	0	Small number of participants (N=28)
Kishk R et al, 2020	Egypt	0	0	Small number of participants (N=30)
Makhlouf N et al, 2014	Egypt	14.29	0	Small number of participants (N=14)
Schulpis K et al, 2008	Greece	0	0	Exclusion of premature new-born and small number of participants (N=28)
Thumbiran N et al, 2014	South Africa	NR	0	Small number of participants (N=14)

* Estimates that are <5 % were considered to be the outliers.

Supplementary Results 5B. Studies showing substantial deviation towards higher estimates (n=18)

Author, year	Country	% with high viral load	% with positive HBeAg	Potential reasons
Bzowej N, 2019	USA	30.77	37.09	Specialized tertiary center
Chakvetadze C, 2011	Mayotte	59.65	60.22	Specialized tertiary center
Chen J, 2014	China	NR	61.38	Biological study
Ding Y, 2013	China	NR	67.07	No apparent reason
Dwivedi M, 2011	India	NR	56.76	Small number of participants (N=37)
Li Y, 2020	China	75.00	77.59	Specialized tertiary center
Liu C, 2015	China	NR	67.19	Selection based on fully vaccinated newborn with HBIG only
Liu J, 2018	China	NR	58.80	Specialized tertiary center
Liu Z, 2019	China	50.00	62.82	Specialized tertiary center
Nguyen G, 2009	USA	55.56	44.83	Specialized tertiary center and small number of participants (N=27)
Pan C, 2013	China	47.61	54.83	Selection based on fully vaccinated newborn with HBIG only
Ter Borg M, 2008	Netherlands	NR	63.16	Specialized tertiary center and small number of participants (N=38)
Wang Z, 2003	China	NR	61.11	Specialized tertiary center
Andreotti M, 2014	Malawi	NR	37.04	HIV and small number of participants (N=27)
Chasela C, 2014	Malawi	NR	38.24	HIV
Dachlan E, 2020	Indonesia	NR	30.30	Specialized tertiary center and small number of participants (N=33)
Foaud H, 2019	Egypt	18.75	33.33	Specialized tertiary center and small number of participants (N=48)
Sirilert S, 2019	Thaïland	NR	31.03	Specialized tertiary center

* Estimates that are >50 % for WPR and >30% for other regions were considered to be the outliers.

Table S1. Subgroup analyses for the proportion of HBV-infected pregnant women with high HBV DNA levels (67 cohorts from 67 studies)

Variables	WPR (33 cohorts from 33 studies)				Other WHO regions (34 cohorts from 34 studies)			
	Cohorts (n)	Pooled estimate	95% CI	p-value (Moderators)	Cohorts (n)	Pooled estimate	95% CI	p-value (Moderators)
**Clinical heterogeneity**								
**Viral load cut-off (log10 IU/mL)**								
5.0 - 5.2	5	0.3642	[0.3076; 0.4248]	0.6035	2	0.3728	[0.1379; 0.6884]	0.0217
5.3	26	0.3031	[0.2518; 0.3599]		28	0.1269	[0.0960; 0.1658]	
5.4 - 6.0	2	0.3475	[0.1817; 0.5609]		4	0.0916	[0.0716; 0.1165]	
**Mean or median age of women***								
< 29 yo	8	0.2968	[0.1961; 0.4222]	0.9979	9	0.0985	[0.0355; 0.2447]	0.5625
≥ 29 yo	7	0.2981	[0.2115; 0.4021]		12	0.1479	[0.0932; 0.2267]	
**Median recruitment year****								
< 2011	14	0.2817	[0.2248; 0.3465]	0.1790	15	0.1423	[0.0928; 0.2120]	0.7094
≥ 2011	19	0.3430	[0.2818; 0.4098]		15	0.1334	[0.0956; 0.1830]	
**HIV co-infection in women**								
None reported to be co-infected with HIV	31	0.3122	[0.2662; 0.3622]	0.7424	26	0.1360	[0.0983; 0.1853]	0.8221
Some co-infected with HIV	1	0.3226	[0.1832; 0.5028]		6	0.1117	[0.0688; 0.1763]	
All co-infected with HIV	1	0.4248	[0.3490; 0.5044]		2	0.1335	[0.0573; 0.2810]	
**Methodological heterogeneity**								
**Study design**								
Prospective	23	0.3121	[0.2531; 0.3780]	0.8452	15	0.1236	[0.0903; 0.1670]	0.0896

Retrospective	8	0.3352	[0.2766; 0.3994]		11	0.1825	[0.1125; 0.2823]	
Cross sectional	2	0.2738	[0.1967; 0.3673]		8	0.0831	[0.0405; 0.1629]	
**Recruitment site*** **								
Primary care	9	0.3230	[0.2720; 0.3786]	0.8797	10	0.1359	[0.1041; 0.1756]	0.9766
Referral centre	24	0.3128	[0.2552; 0.3768]		23	0.1292	[0.0870; 0.1875]	
**HBsAg screening process fully described**								
Yes	17	0.2500	[0.2048; 0.3013]	< 0.0001	19	0.1105	[0.0831; 0.1453]	0.0675
No	16	0.3922	[0.3346; 0.4530]		15	0.1657	[0.1066; 0.2486]	
**Rate of uptake for HBV DNA quantification****** **								
≥ 75%	14	0.2914	[0.2409; 0.3476]	0.7650	11	0.1190	[0.0746; 0.1846]	0.7040
< 75%	12	0.3042	[0.2287; 0.3921]		12	0.1342	[0.0891; 0.1970]	

* Mean or median age was not reported in 31 cohorts.
** Median recruitment year was not reported in 4 cohorts.
*** Recruitment site was not reported in 1 cohort.
**** Rate of uptake for HBV DNA quantification was not reported in 18 cohorts.

Table S2. Subgroup analyses for the proportion of HBV-infected pregnant women who test positive for HBeAg (129 cohorts from 125 studies)

Variables	WPR (63 cohorts)				Other WHO regions (66 cohorts)			
	Cohorts (n)	Pooled estimate	95% CI	p-value (Moderators)	Cohorts (n)	Pooled estimate	95% CI	p-value (Moderators)
**Clinical heterogeneity**								
**HBeAg test***								
EIA	27	0.3223	[0.2808; 0.3668]	0.0697	30	0.1296	[0.0844; 0.1940]	0.5318
CLIA	21	0.3977	[0.3419; 0.4562]		11	0.1800	[0.1300; 0.2440]	
RDT	2	0.2133	[0.1921; 0.2362]		3	0.1000	[0.0802; 0.1240]	
FIA	2	0.3366	[0.2748; 0.4046]		2	0.1526	[0.0677; 0.3086]	
**Median or mean age of women****								
< 29 yo	11	0.3965	[0.3197; 0.4787]	0.0154	15	0.1529	[0.0796; 0.2736]	0.9415
≥ 29 yo	15	0.2938	[0.2578; 0.3326]		16	0.1736	[0.1258; 0.2346]	
**Median recruitment year*****								
< 2011	36	0.3438	[0.3030; 0.3869]	0.9144	42	0.1446	[0.1078; 0.1912]	0.5734
≥ 2011	27	0.3473	[0.2977; 0.4005]		21	0.1757	[0.1372; 0.2222]	
**HIV co-infection in women**								
None reported to be co-infected with HIV	61	0.3453	[0.3125; 0.3795]	0.8716	50	0.1363	[0.1032; 0.1779]	0.5348
Some co-infected with HIV	1	0.2903	[0.1585; 0.4705]		14	0.1875	[0.1389; 0.2482]	
All co-infected with HIV	1	0.3922	[0.3180; 0.4716]		2	0.2079	[0.1786; 0.2406]	
**Methodological heterogeneity**								
**Study design**								
Prospective	40	0.3507	[0.3088; 0.3951]	0.9127	23	0.1736	[0.1278; 0.2314]	0.0264

Retrospective	16	0.3370	[0.2822; 0.3964]		22	0.1817	[0.1339; 0.2419]	
Cross sectional	7	0.3350	[0.2494; 0.4332]		21	0.0838	[0.0466; 0.1462]	
**Recruitment site******								
Primary care	14	0.3452	[0.2880; 0.4073]	0.8504	21	0.1354	[0.1032; 0.1756]	0.3402
Referral center	47	0.3377	[0.3008; 0.3768]		44	0.1584	[0.1185; 0.2087]	
**HBsAg screening process fully described**								
Yes	22	0.3017	[0.2638; 0.3425]	0.0397	38	0.1291	[0.0951; 0.1730]	0.1561
No	41	0.3698	[0.3269; 0.4150]		28	0.1761	[0.1308; 0.2329]	
**Rate of uptake for HBeAg testing*******								
≥ 75%	29	0.3268	[0.2856; 0.3708]	0.4390	30	0.1418	[0.0996; 0.1980]	0.5912
< 75%	15	0.3561	[0.2982; 0.4185]		12	0.1727	[0.1333; 0.2208]	

* Type of HBeAg assay was not reported in 31 cohorts.
** Median or mean age was not reported in 72 cohorts.
*** Median recruitment year was not reported in 3 cohorts.
**** Recruitment site was not reported in 3 cohrots.
***** Rate of uptake for HBeAg testing was not reported in 43 cohorts.

Fig. S1. Proportion of HBV-infected pregnant women in subgroups defined by both HBV DNA levels and HBeAg status according to the WHO regions

Fig. S1A. Proportion of HBV-infected pregnant women with high viral load and1 positive HBeAg

Author	Country	Number of PW with HVL and HBeAg positive status	Total PW	Prop (%)	95% CI
WHO_regions = WPR					
Nishimura K, 2021	Japan	29	87	33.33	[23.58; 44.25]
Ségéral O, 2022	Cambodia	240	1194	20.10	[17.86; 22.49]
Bergin H, 2017	Australia	20	87	22.99	[14.64; 33.25]
Thilakanathan C, 2018	Australia	168	642	26.17	[22.81; 29.75]
Ségéral O, 2018	Cambodia	26	128	20.31	[13.72; 28.33]
Wang L, 2016	China	9	31	29.03	[14.22; 48.04]
Hu Y, 2016	China	68	264	25.76	[20.59; 31.48]
Li L, 2020	China	138	317	43.53	[38.00; 49.19]
Li Y, 2020	China	174	232	75.00	[68.91; 80.44]
Liu Z, 2019	China	411	893	46.02	[42.72; 49.36]
Lu Y, 2017	China	364	1177	30.93	[28.29; 33.65]
Xu C, 2018	China	68	214	31.78	[25.60; 38.47]
Michitaka K, 2012	Japan	4	21	19.05	[5.45; 41.91]
Sasagawa Y, 2019	Japan	14	35	40.00	[23.87; 57.89]
Latthaphasavang V, 2019	Laos	51	153	33.33	[25.93; 41.40]
Lee L, 2015	Singapore	33	154	21.43	[15.23; 28.76]
Khue P, 2020	Vietnam	22	183	12.02	[7.69; 17.63]
Pooled estimate				**30.28**	**[23.86; 37.57]**
Heterogeneity: I^2 = 96%, τ^2 = 0.4229, p < 0.01					
WHO_regions = AFR					
Guingané A, 2022	Burkina Faso	38	578	6.57	[4.69; 8.91]
Shimakawa Y, 2022	Cameroon	110	594	18.52	[15.47; 21.88]
Loarec A, 2022	Mozambique	12	252	4.76	[2.48; 8.17]
Geffert K, 2020	Tanzania	1	22	4.55	[0.12; 22.84]
Pooled estimate				**8.05**	**[4.15; 15.05]**
Heterogeneity: I^2 = 94%, τ^2 = 0.3845, p < 0.01					
WHO_regions = AMR					
Van Ommen C, 2019	Canada	4	59	6.78	[1.88; 16.46]
Izquierdo G, 2019	Chile	5	30	16.67	[5.64; 34.72]
Kubo A, 2014	USA	127	835	15.21	[12.84; 17.83]
Lyu J, 2019	USA	255	1241	20.55	[18.33; 22.90]
Nguyen G, 2009	USA	10	27	37.04	[19.40; 57.63]
Patel N, 2019	Canada	10	41	24.39	[12.36; 40.30]
Pooled estimate				**18.27**	**[13.33; 24.53]**
Heterogeneity: I^2 = 76%, τ^2 = 0.1300, p < 0.01					
WHO_regions = EMR					
El−Karaksy H, 2014	Egypt	1	35	2.86	[0.07; 14.92]
Foaud H, 2019	Egypt	7	48	14.58	[6.07; 27.76]
Kishk R, 2020	Egypt	0	30	0.00	[0.00; 11.57]
Makhlouf N, 2014	Egypt	0	14	0.00	[0.00; 23.16]
Pooled estimate				**2.78**	**[0.34; 19.45]**
Heterogeneity: I^2 = 0%, τ^2 = 1.5563, p = 0.46					
WHO_regions = EUR					
Sellier P, 2018	France	2	12	16.67	[2.09; 48.41]
Elefsiniotis I, 2007	Greece	1	63	1.59	[0.04; 8.53]
Papaevangelou V, 2011	Greece	5	109	4.59	[1.51; 10.38]
Schulpis K, 2008	Greece	0	28	0.00	[0.00; 12.34]
Ruiz−Extremera Á, 2020	Spain	2	62	3.23	[0.39; 11.17]
Eilard A, 2019	Sweden	6	42	14.29	[5.43; 28.54]
Bhattacharya S, 2008	United Kingdom	5	112	4.46	[1.47; 10.11]
Godbole G, 2013	United Kingdom	20	293	6.83	[4.22; 10.35]
White H, 2015	United Kingdom	12	123	9.76	[5.14; 16.42]
Pooled estimate				**5.98**	**[4.06; 8.73]**
Heterogeneity: I^2 = 33%, τ^2 = 0.0911, p = 0.16					
**Pooled estimate**				**15.24**	**[11.12; 20.53]**
Heterogeneity: I^2 = 95%, τ^2 = 1.1562, p < 0.01					
Test for subgroup differences: χ^2_4 = 60.72, df = 4 (p < 0.01)					

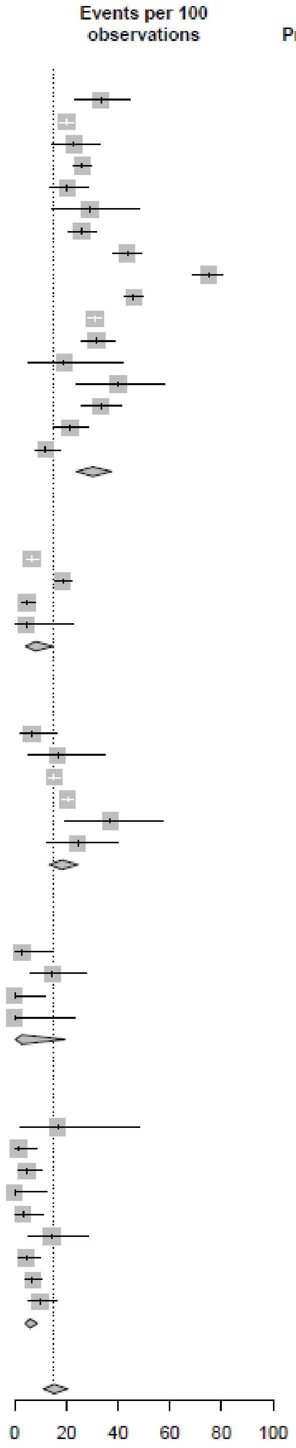

Fig. S1B. Proportion of HBV-infected pregnant women with high viral load and negative HBeAg

Author	Country	Number of PW with HVL and HBeAg negative status	Total PW	Prop (%)	95% CI
**WHO_regions = WPR**					
Nishimura K, 2021	Japan	4	87	4.60	[1.27; 11.36]
Ségéral O, 2022	Cambodia	127	1194	10.64	[8.94; 12.53]
Bergin H, 2017	Australia	5	87	5.75	[1.89; 12.90]
Thilakanathan C, 2018	Australia	17	642	2.65	[1.55; 4.21]
Ségéral O, 2018	Cambodia	8	128	6.25	[2.74; 11.94]
Wang L, 2016	China	1	31	3.23	[0.08; 16.70]
Hu Y, 2016	China	7	264	2.65	[1.07; 5.39]
Li L, 2020	China	18	317	5.68	[3.40; 8.83]
Li Y, 2020	China	0	232	0.00	[0.00; 1.58]
Liu Z, 2019	China	22	893	2.46	[1.55; 3.71]
Lu Y, 2017	China	24	1177	2.04	[1.31; 3.02]
Xu C, 2018	China	6	214	2.80	[1.04; 6.00]
Michitaka K, 2012	Japan	2	21	9.52	[1.17; 30.38]
Sasagawa Y, 2019	Japan	3	35	8.57	[1.80; 23.06]
Latthaphasavang V, 2019	Laos	14	153	9.15	[5.09; 14.88]
Lee L, 2015	Singapore	0	154	0.00	[0.00; 2.37]
Khue P, 2020	Vietnam	2	183	1.09	[0.13; 3.89]
Pooled estimate				**3.27**	**[2.11; 5.04]**
Heterogeneity: $I^2 = 87\%$, $\tau^2 = 0.6411$, $p < 0.01$					
**WHO_regions = AFR**					
Guingané A, 2022	Burkina Faso	18	578	3.11	[1.86; 4.88]
Shimakawa Y, 2022	Cameroon	11	594	1.85	[0.93; 3.29]
Loarec A, 2022	Mozambique	1	252	0.40	[0.01; 2.19]
Geffert K, 2020	Tanzania	0	22	0.00	[0.00; 15.44]
Pooled estimate				**1.70**	**[0.77; 3.69]**
Heterogeneity: $I^2 = 43\%$, $\tau^2 = 0.2115$, $p = 0.15$					
**WHO_regions = AMR**					
Van Ommen C, 2019	Canada	2	59	3.39	[0.41; 11.71]
Izquierdo G, 2019	Chile	0	30	0.00	[0.00; 11.57]
Kubo A, 2014	USA	20	835	2.40	[1.47; 3.68]
Lyu J, 2019	USA	23	1241	1.85	[1.18; 2.77]
Nguyen G, 2009	USA	5	27	18.52	[6.30; 38.08]
Patel N, 2019	Canada	2	41	4.88	[0.60; 16.53]
Pooled estimate				**3.24**	**[1.56; 6.64]**
Heterogeneity: $I^2 = 78\%$, $\tau^2 = 0.5316$, $p < 0.01$					
**WHO_regions = EMR**					
El−Karaksy H, 2014	Egypt	1	35	2.86	[0.07; 14.92]
Foaud H, 2019	Egypt	9	48	18.75	[8.95; 32.63]
Kishk R, 2020	Egypt	0	30	0.00	[0.00; 11.57]
Makhlouf N, 2014	Egypt	2	14	14.29	[1.78; 42.81]
Pooled estimate				**6.19**	**[1.57; 21.38]**
Heterogeneity: $I^2 = 18\%$, $\tau^2 = 1.0608$, $p = 0.30$					
**WHO_regions = EUR**					
Sellier P, 2018	France	0	12	0.00	[0.00; 26.46]
Elefsiniotis I, 2007	Greece	7	63	11.11	[4.59; 21.56]
Papaevangelou V, 2011	Greece	1	109	0.92	[0.02; 5.01]
Schulpis K, 2008	Greece	0	28	0.00	[0.00; 12.34]
Ruiz−Extremera Á, 2020	Spain	2	62	3.23	[0.39; 11.17]
Eilard A, 2019	Sweden	0	42	0.00	[0.00; 8.41]
Bhattacharya S, 2008	United Kingdom	0	112	0.00	[0.00; 3.24]
Godbole G, 2013	United Kingdom	6	293	2.05	[0.76; 4.40]
White H, 2015	United Kingdom	1	123	0.81	[0.02; 4.45]
Pooled estimate				**1.25**	**[0.39; 3.92]**
Heterogeneity: $I^2 = 47\%$, $\tau^2 = 1.2341$, $p = 0.05$					
**Pooled estimate**				**2.70**	**[1.88; 3.86]**

Heterogeneity: $I^2 = 83\%$, $\tau^2 = 0.8797$, $p < 0.01$

Test for subgroup differences: $\chi^2_4 = 5.43$, df = 4 ($p = 0.25$)

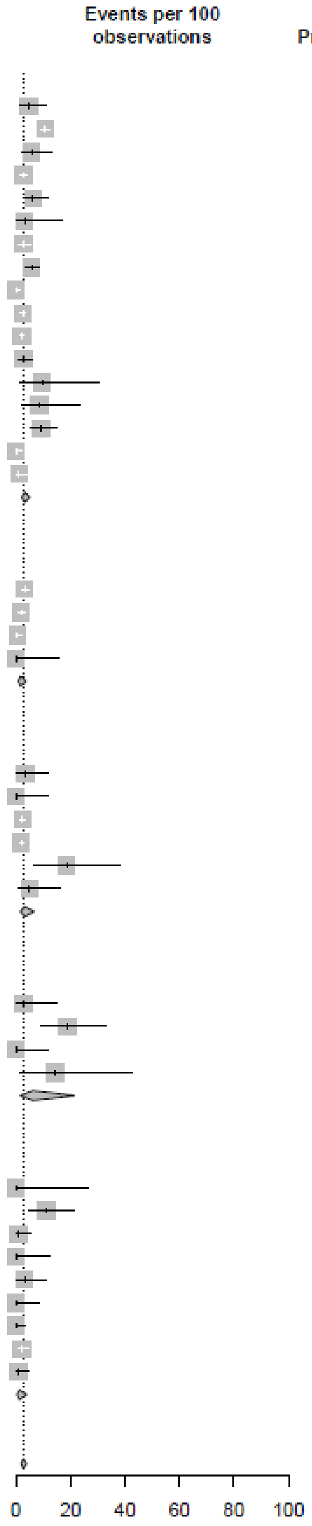

Fig. S1C. Proportion of HBV-infected pregnant women with low viral load and positive HBeAg

Author	Country	Number of PW with LVL and HBeAg positive status	Total PW	Prop (%)	95% CI
**WHO_regions = WPR**					
Nishimura K, 2021	Japan	8	87	9.20	[4.05; 17.32]
Ségéral O, 2022	Cambodia	13	1194	1.09	[0.58; 1.85]
Bergin H, 2017	Australia	4	87	4.60	[1.27; 11.36]
Thilakanathan C, 2018	Australia	24	642	3.74	[2.41; 5.51]
Ségéral O, 2018	Cambodia	3	128	2.34	[0.49; 6.70]
Wang L, 2016	China	0	31	0.00	[0.00; 11.22]
Hu Y, 2016	China	8	264	3.03	[1.32; 5.88]
Li L, 2020	China	9	317	2.84	[1.31; 5.32]
Li Y, 2020	China	16	232	6.90	[3.99; 10.96]
Liu Z, 2019	China	150	893	16.80	[14.40; 19.41]
Lu Y, 2017	China	55	1177	4.67	[3.54; 6.04]
Xu C, 2018	China	4	214	1.87	[0.51; 4.72]
Michitaka K, 2012	Japan	0	21	0.00	[0.00; 16.11]
Sasagawa Y, 2019	Japan	2	35	5.71	[0.70; 19.16]
Latthaphasavang V, 2019	Laos	9	153	5.88	[2.72; 10.87]
Lee L, 2015	Singapore	8	154	5.19	[2.27; 9.98]
Khue P, 2020	Vietnam	20	183	10.93	[6.80; 16.37]
Pooled estimate				**4.23**	**[2.86; 6.19]**
Heterogeneity: $I^2 = 93\%$, $\tau^2 = 0.5169$, $p < 0.01$					
**WHO_regions = AFR**					
Guingané A, 2022	Burkina Faso	24	578	4.15	[2.68; 6.12]
Shimakawa Y, 2022	Cameroon	17	594	2.86	[1.68; 4.54]
Loarec A, 2022	Mozambique	12	252	4.76	[2.48; 8.17]
Geffert K, 2020	Tanzania	1	22	4.55	[0.12; 22.84]
Pooled estimate				**3.73**	**[2.87; 4.84]**
Heterogeneity: $I^2 = 0\%$, $\tau^2 = 0$, $p = 0.52$					
**WHO_regions = AMR**					
Van Ommen C, 2019	Canada	2	59	3.39	[0.41; 11.71]
Izquierdo G, 2019	Chile	0	30	0.00	[0.00; 11.57]
Kubo A, 2014	USA	36	835	4.31	[3.04; 5.92]
Lyu J, 2019	USA	60	1241	4.83	[3.71; 6.18]
Nguyen G, 2009	USA	2	27	7.41	[0.91; 24.29]
Patel N, 2019	Canada	2	41	4.88	[0.60; 16.53]
Pooled estimate				**4.57**	**[3.78; 5.52]**
Heterogeneity: $I^2 = 0\%$, $\tau^2 = 0$, $p = 0.96$					
**WHO_regions = EMR**					
El−Karaksy H, 2014	Egypt	2	35	5.71	[0.70; 19.16]
Foaud H, 2019	Egypt	2	48	4.17	[0.51; 14.25]
Kishk R, 2020	Egypt	0	30	0.00	[0.00; 11.57]
Makhlouf N, 2014	Egypt	0	14	0.00	[0.00; 23.16]
Pooled estimate				**3.15**	**[1.19; 8.09]**
Heterogeneity: $I^2 = 0\%$, $\tau^2 = 0$, $p = 0.99$					
**WHO_regions = EUR**					
Sellier P, 2018	France	1	12	8.33	[0.21; 38.48]
Elefsiniotis I, 2007	Greece	0	63	0.00	[0.00; 5.69]
Papaevangelou V, 2011	Greece	3	109	2.75	[0.57; 7.83]
Schulpis K, 2008	Greece	0	28	0.00	[0.00; 12.34]
Ruiz−Extremera Á, 2020	Spain	7	62	11.29	[4.66; 21.89]
Eilard A, 2019	Sweden	1	42	2.38	[0.06; 12.57]
Bhattacharya S, 2008	United Kingdom	2	112	1.79	[0.22; 6.30]
Godbole G, 2013	United Kingdom	6	293	2.05	[0.76; 4.40]
White H, 2015	United Kingdom	1	123	0.81	[0.02; 4.45]
Pooled estimate				**2.19**	**[1.04; 4.56]**
Heterogeneity: $I^2 = 49\%$, $\tau^2 = 0.5566$, $p = 0.05$					
**Pooled estimate**				**3.69**	**[2.86; 4.75]**
Heterogeneity: $I^2 = 86\%$, $\tau^2 = 0.3848$, $p < 0.01$					
Test for subgroup differences: $\chi^2_4 = 4.84$, df = 4 ($p = 0.30$)					

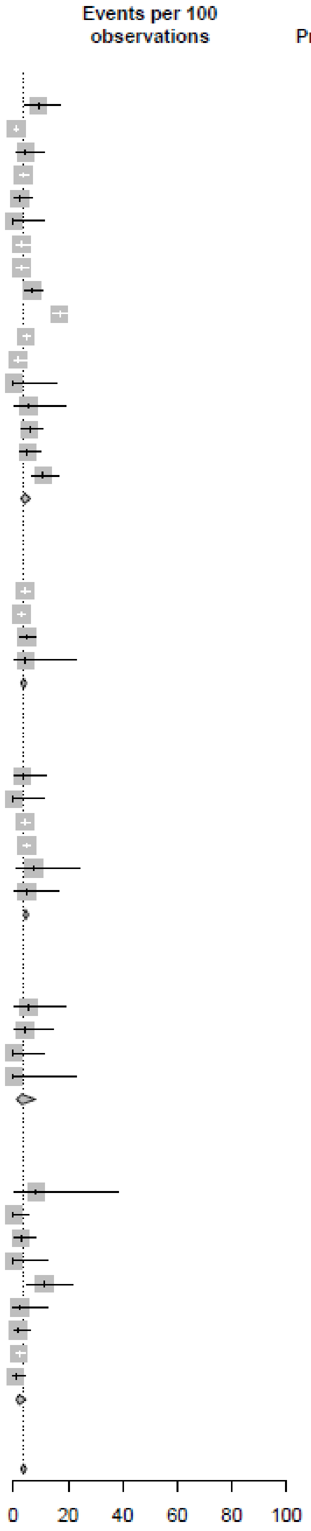

Fig. S1D. Proportion of HBV-infected pregnant women with low viral load and negative HBeAg

Author	Country	Number of PW with LVL and HBeAg negative status	Total PW	Prop (%)	95% CI
WHO_regions = WPR					
Nishimura K, 2021	Japan	46	87	52.87	[41.87; 63.67]
Ségéral O, 2022	Cambodia	814	1194	68.17	[65.45; 70.81]
Bergin H, 2017	Australia	58	87	66.67	[55.75; 76.42]
Thilakanathan C, 2018	Australia	433	642	67.45	[63.67; 71.06]
Ségéral O, 2018	Cambodia	91	128	71.09	[62.42; 78.76]
Wang L, 2016	China	21	31	67.74	[48.63; 83.32]
Hu Y, 2016	China	181	264	68.56	[62.59; 74.11]
Li L, 2020	China	152	317	47.95	[42.33; 53.60]
Li Y, 2020	China	42	232	18.10	[13.37; 23.67]
Liu Z, 2019	China	310	893	34.71	[31.59; 37.94]
Lu Y, 2017	China	735	1177	62.45	[59.61; 65.22]
Xu C, 2018	China	136	214	63.55	[56.72; 70.00]
Michitaka K, 2012	Japan	15	21	71.43	[47.82; 88.72]
Sasagawa Y, 2019	Japan	16	35	45.71	[28.83; 63.35]
Latthaphasavang V, 2019	Laos	79	153	51.63	[43.42; 59.78]
Lee L, 2015	Singapore	113	154	73.38	[65.66; 80.17]
Khue P, 2020	Vietnam	139	183	75.96	[69.10; 81.95]
Pooled estimate				**59.31**	**[51.30; 66.85]**
Heterogeneity: I^2 = 97%, τ^2 = 0.4246, p < 0.01					
WHO_regions = AFR					
Guingané A, 2022	Burkina Faso	498	578	86.16	[83.07; 88.87]
Shimakawa Y, 2022	Cameroon	456	594	76.77	[73.16; 80.11]
Loarec A, 2022	Mozambique	227	252	90.08	[85.70; 93.48]
Geffert K, 2020	Tanzania	20	22	90.91	[70.84; 98.88]
Pooled estimate				**85.48**	**[78.88; 90.28]**
Heterogeneity: I^2 = 90%, τ^2 = 0.1498, p < 0.01					
WHO_regions = AMR					
Van Ommen C, 2019	Canada	51	59	86.44	[75.02; 93.96]
Izquierdo G, 2019	Chile	25	30	83.33	[65.28; 94.36]
Kubo A, 2014	USA	652	835	78.08	[75.12; 80.85]
Lyu J, 2019	USA	903	1241	72.76	[70.19; 75.22]
Nguyen G, 2009	USA	10	27	37.04	[19.40; 57.63]
Patel N, 2019	Canada	27	41	65.85	[49.41; 79.92]
Pooled estimate				**72.96**	**[60.48; 82.63]**
Heterogeneity: I^2 = 84%, τ^2 = 0.4105, p < 0.01					
WHO_regions = EMR					
El−Karaksy H, 2014	Egypt	31	35	88.57	[73.26; 96.80]
Foaud H, 2019	Egypt	30	48	62.50	[47.35; 76.05]
Kishk R, 2020	Egypt	30	30	100.00	[88.43; 100.00]
Makhlouf N, 2014	Egypt	12	14	85.71	[57.19; 98.22]
Pooled estimate				**88.99**	**[65.07; 97.23]**
Heterogeneity: I^2 = 61%, τ^2 = 1.5077, p = 0.05					
WHO_regions = EUR					
Sellier P, 2018	France	9	12	75.00	[42.81; 94.51]
Elefsiniotis I, 2007	Greece	55	63	87.30	[76.50; 94.35]
Papaevangelou V, 2011	Greece	100	109	91.74	[84.90; 96.15]
Schulpis K, 2008	Greece	28	28	100.00	[87.66; 100.00]
Ruiz−Extremera Á, 2020	Spain	51	62	82.26	[70.47; 90.80]
Eilard A, 2019	Sweden	35	42	83.33	[68.64; 93.03]
Bhattacharya S, 2008	United Kingdom	105	112	93.75	[87.55; 97.45]
Godbole G, 2013	United Kingdom	261	293	89.08	[84.93; 92.41]
White H, 2015	United Kingdom	109	123	88.62	[81.64; 93.64]
Pooled estimate				**89.22**	**[86.94; 91.14]**
Heterogeneity: I^2 = 17%, τ^2 = 0, p = 0.29					
**Pooled estimate**				**75.59**	**[69.15; 81.05]**

Heterogeneity: I^2 = 96%, τ^2 = 0.9601, p < 0.01

Test for subgroup differences: χ^2_4 = 81.05, df = 4 (p < 0.01)

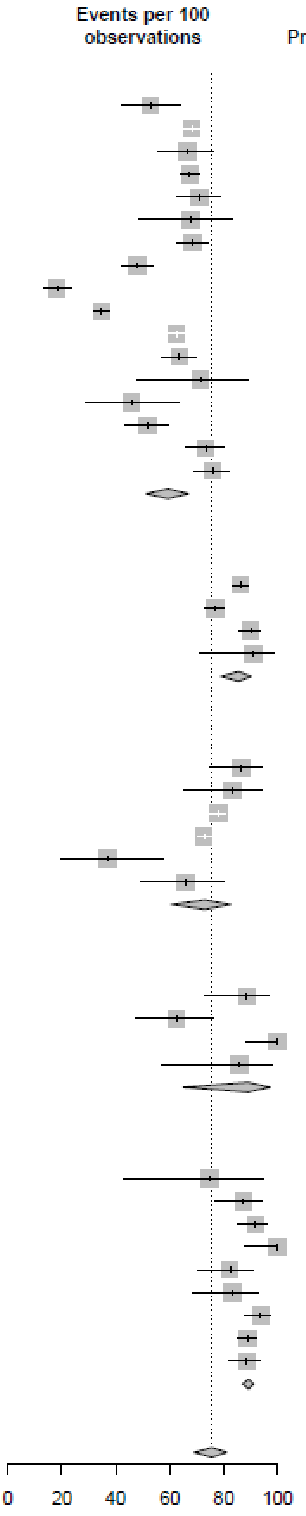

Supplementary references 1. References of studies included in the systematic review

The numbers of cohorts and studies evaluated in each of the questions were as below:

- 67 cohorts from 67 studies for Q1 [1–67]
- 129 cohorts from 125 studies for Q2 [1,3–14,16–30,32–37,39–42,44–57,59–131]
- 40 cohorts from 40 studies for Q3 [4–7,10–14,16–20,22,24–28,30,32,33,35,42,45–47,49,50,52,54–56,59–62,67,122]
- 11 cohorts from 11 studies for Q4 [7,11,18,20,27,28,46,47,50,59,61]

1 Bhattacharya D, Guo R, Tseng C-H, et al. Maternal HBV Viremia and Association With Adverse Infant Outcomes in Women Living With HIV and HBV. Pediatric Infectious Disease Journal 2021;40:e56–61. doi:10.1097/INF.0000000000002980

2 Candotti D, Danso K, Allain J-P. Maternofetal transmission of hepatitis B virus genotype E in Ghana, west Africa. Journal of General Virology 2007;88:2686–95. doi:10.1099/vir.0.83102-0

3 Chakvetadze C, Roussin C, Roux J, et al. Efficacy of hepatitis B sero-vaccination in newborns of African HBsAg positive mothers. Vaccine 2011;29:2846–9. doi:10.1016/j.vaccine.2011.01.101

4 Geffert K, Maponga TG, Henerico S, et al. Prevalence of chronic HBV infection in pregnant woman attending antenatal care in a tertiary hospital in Mwanza, Tanzania: a cross-sectional study. BMC Infectious Diseases 2020;20:395. doi:10.1186/s12879-020-05096-2

5 Nanelin Guingané A, Kaboré R, Shimakawa Y, et al. Screening for Hepatitis B in partners and children of women positive for surface antigen, Burkina Faso. Bull World Health Organ 2022;100:256–67. doi:10.2471/BLT.21.287015

6 Loarec A, Nguyen A, Molfino L, et al. Prevention of mother-to-child transmission of hepatitis B virus in antenatal care and maternity services, Mozambique. Bull World Health Org 2022;100:60–9. doi:10.2471/BLT.20.281311

7 Shimakawa Y, Veillon P, Birguel J, et al. Residual risk of mother-to-child transmission of hepatitis B virus infection despite timely birth-dose vaccination in Cameroon (ANRS 12303): a single-centre, longitudinal observational study. The Lancet Global Health 2022;10:e521–9. doi:10.1016/S2214-109X(22)00026-2

8 Biondi MJ, Marchand-Austin A, Cronin K, et al. Prenatal hepatitis B screening, and hepatitis B burden among children, in Ontario: a descriptive study. CMAJ 2020;192:E1299–305. doi:10.1503/cmaj.200290

9 Bzowej NH, Tran TT, Li R, et al. Total Alanine Aminotransferase (ALT) Flares in Pregnant North American Women With Chronic Hepatitis B Infection: Results From a Prospective Observational Study. Am J Gastroenterol 2019;114:1283–91. doi:10.14309/ajg.0000000000000221

10 Izquierdo G, Bustos S, González Á, et al. Cribado de virus de hepatitis B en mujeres embarazadas: inmigrantes, y chilenas con conductas de riesgo. Manejo del binomio madre-hijo: Plan piloto. Rev chil infectol 2019;36:576–84. doi:10.4067/S0716-10182019000500576

11 Kubo A, Shlager L, Marks AR, et al. Prevention of Vertical Transmission of Hepatitis B: An Observational Study. Ann Intern Med 2014;160:828. doi:10.7326/M13-2529

12 Lyu J, Wang S, He Q, et al. Hep B Moms: A cross-sectional study of mother-to-child transmission risk among pregnant Asian American women with chronic hepatitis B in New York City, 2007-2017. Journal of Viral Hepatitis 2020;27:168–75. doi:10.1111/jvh.13221

13 Nguyen G, Garcia RT, Nguyen N, et al. Clinical course of hepatitis B virus infection during pregnancy. Alimentary Pharmacology & Therapeutics 2009;29:755–64. doi:10.1111/j.1365-2036.2009.03932.x

14 Patel NH, Joshi SS, Lau KCK, et al. Analysis of serum hepatitis B virus RNA levels in a multiethnic cohort of pregnant chronic hepatitis B carriers. Journal of Clinical Virology 2019;111:42–7. doi:10.1016/j.jcv.2019.01.002

15 Tohme RA, Andre-Alboth J, Tejada-Strop A, et al. Hepatitis B virus infection among pregnant women in Haiti: A cross-sectional serosurvey. Journal of Clinical Virology 2016;76:66–71. doi:10.1016/j.jcv.2016.01.012

16 Ommen CV, Albert A, Nourmoussavi M, et al. Stability of hepatitis B viral load during pregnancy and implications for antepartum prophylaxis: A prospective cohort study. Canadian Liver Journal 2019;2:190–8. doi:10.3138/canlivj.2019-0001

17 El-Karaksy HM. Applicability and efficacy of a model for prevention of perinatal transmission of hepatitis B virus infection: Single center study in Egypt. WJG 2014;20:17075. doi:10.3748/wjg.v20.i45.17075

18 Foaud HM, Maklad S, Gmal El Din A, et al. Lamivudine use in pregnant HBsAg-females effectively reduces maternal viremia. Arab Journal of Gastroenterology 2019;20:8–13. doi:10.1016/j.ajg.2019.02.003

19 Kishk R, Mandour M, Elprince M, et al. Pattern and interpretation of hepatitis B virus markers among pregnant women in North East Egypt. Braz J Microbiol 2020;51:593–600. doi:10.1007/s42770-019-00174-3

20 Makhlouf N, Morsy K, Othman E, et al. Ante-natal screening of pregnant women for hepatitis B virus infection in Upper Egypt: A Tertiary Care Center Based Study. Egyptian Liver Journal 2014;4:57–62. doi:10.1097/01.ELX.0000445723.55972.3a

21 Belopolskaya M, Avrutin V, Firsov S, et al. HBsAg level and hepatitis B viral load correlation with focus on pregnancy. Ann Gastroenterol 2015;28:379–84.

22 Bhattacharya S, O'Donnell K, Dudley T, et al. Ante-natal screening and post-natal follow-up of hepatitis B in the West Midlands of England. QJM 2008;101:307–12. doi:10.1093/qjmed/hcn007

23 Dyson JK, Waller J, Turley A, et al. Hepatitis B in pregnancy. Frontline Gastroenterol 2014;5:111–7. doi:10.1136/flgastro-2013-100361

24 Eilard A, Andersson M, Ringlander J, et al. Vertically acquired occult hepatitis B virus infection may become overt after several years. J Infect 2019;78:226–31. doi:10.1016/j.jinf.2019.01.002

25 Elefsiniotis IS, Glynou I, Brokalaki H, et al. Serological and virological profile of chronic HBV infected women at reproductive age in Greece: A two-year single center study. European Journal of Obstetrics & Gynecology and Reproductive Biology 2007;132:200–3. doi:10.1016/j.ejogrb.2006.08.015

26 Godbole G, Irish D, Basarab M, et al. Management of hepatitis B in pregnant women and infants: a multicentre audit from four London hospitals. BMC Pregnancy and Childbirth 2013;13:222. doi:10.1186/1471-2393-13-222

27 Papaevangelou V, Paraskevis D, Anastassiadou V, et al. HBV viremia in newborns of HBsAg(+) predominantly Caucasian HBeAg(-) mothers. J Clin Virol 2011;50:249–52. doi:10.1016/j.jcv.2010.11.009

28 Ruiz-Extremera Á, Díaz-Alcázar M del M, Muñoz-Gámez JA, et al. Seroprevalence and epidemiology of hepatitis B and C viruses in pregnant women in Spain. Risk factors for vertical transmission. PLoS ONE 2020;15:e0233528. doi:10.1371/journal.pone.0233528

29 Safadi R, Khoury T, Saed N, et al. Efficacy of Birth Dose Vaccination in Preventing Mother-to-Child Transmission of Hepatitis B: A Randomized Controlled Trial Comparing Engerix-B and Sci-B-Vac. Vaccines 2021;9:331. doi:10.3390/vaccines9040331

30 Schulpis KH, Barzeliotou A, Papadakis M, et al. Maternal chronic hepatitis B virus is implicated with low neonatal paraoxonase/arylesterase activities. Clinical Biochemistry 2008;41:282–7. doi:10.1016/j.clinbiochem.2007.10.013

31 Sellier P, Maylin S, Amarsy R, et al. Untreated highly viraemic pregnant women from Asia or sub-Saharan Africa often transmit hepatitis B virus despite serovaccination to newborns. Liver International 2015;35:409–16. doi:10.1111/liv.12561

32 Sellier PO, Maylin S, Brichler S, et al. Hepatitis B Virus-Hepatitis D Virus mother-to-child co-transmission: A retrospective study in a developed country. Liver International 2018;38:611–8. doi:10.1111/liv.13556

33 White HA, Wiselka MJ, Wilson DJ. Antenatal hepatitis B in a large teaching NHS Trust – Implications for future care. Journal of Infection 2015;70:72–7. doi:10.1016/j.jinf.2014.07.028

34 Fujiko M, Chalid MT, Turyadi, et al. Chronic hepatitis B in pregnant women: is hepatitis B surface antigen quantification useful for viral load prediction? International Journal of Infectious Diseases 2015;41:83–9. doi:10.1016/j.ijid.2015.11.002

35 Bergin H, Wood G, Walker SP, et al. Perinatal management of hepatitis B virus: Clinical implementation of updated Australasian management guidelines. Obstet Med 2018;11:23–7. doi:10.1177/1753495X17731990

36 Chen T, Wang J, Qiu H, et al. Different interventional criteria for chronic hepatitis B pregnant women with HBeAg(+) or HBeAg(-): Epidemiological data from Shaanxi, China. Medicine 2018;97:e11406. doi:10.1097/MD.0000000000011406

37 Chen Y, Wang L, Xu Y, et al. Role of maternal viremia and placental infection in hepatitis B virus intrauterine transmission. Microbes and Infection 2013;15:409–15. doi:10.1016/j.micinf.2013.02.008

38 Chen Z-X, Gu G-F, Bian Z-L, et al. Clinical course and perinatal transmission of chronic hepatitis B during pregnancy: A real-world prospective cohort study. Journal of Infection 2017;75:146–54. doi:10.1016/j.jinf.2017.05.012

39 Cheung KW, Seto MTY, Kan ASY, et al. Immunoprophylaxis Failure of Infants Born to Hepatitis B Carrier Mothers Following Routine Vaccination. Clin Gastroenterol Hepatol 2018;16:144–5. doi:10.1016/j.cgh.2017.07.013

40 Evans AA, Cohen C, Huang P, et al. Prevention of perinatal hepatitis B transmission in Haimen City, China: Results of a community public health initiative. Vaccine 2015;33:3010–5. doi:10.1016/j.vaccine.2015.01.054

41 Guo Z, Shi XH, Feng YL, et al. Risk factors of HBV intrauterine transmission among HBsAg-positive pregnant women. J Viral Hepat 2013;20:317–21. doi:10.1111/jvh.12032

42 Hu Y, Feng Z, Liu J, et al. Virological Determinants of Spontaneous Postpartum e Antigen Seroconversion and Surface Antigen Seroclearance in Pregnant Women Infected with Hepatitis B Virus. Archives of Medical Research 2016;47:207–13. doi:10.1016/j.arcmed.2016.06.008

43 Hui P, Ng C, Cheung K, et al. Acceptance of antiviral treatment and enhanced service model for pregnant patients carrying hepatitis B. Hong Kong Med J Published Online First: 12 August 2020. doi:10.12809/hkmj208451

44 Kang W, Ding Z, Shen L, et al. Risk factors associated with immunoprophylaxis failure against mother to child transmission of hepatitis B virus and hepatitis B vaccination status in Yunnan province, China. Vaccine 2014;32:3362–6. doi:10.1016/j.vaccine.2014.04.045

45 Khue PM, Thuy Linh NT, Vinh VH, et al. Hepatitis B Infection and Mother-to-Child Transmission in Haiphong, Vietnam: A Cohort Study with Implications for Interventions. BioMed Research International 2020;2020:1–12. doi:10.1155/2020/4747965

46 Latthaphasavang V, Vanhems P, Ngo-Giang-Huong N, et al. Perinatal hepatitis B virus transmission in Lao PDR: A prospective cohort study. PLoS ONE 2019;14:e0215011. doi:10.1371/journal.pone.0215011

47 Lee LY, Aw M, Rauff M, et al. Hepatitis B immunoprophylaxis failure and the presence of hepatitis B surface gene mutants in the affected children. Journal of Medical Virology 2015;87:1344–50. doi:10.1002/jmv.24193

48 Li F, Wang Q, Zhang L, et al. The risk factors of transmission after the implementation of the routine immunization among children exposed to HBV infected mothers in a developing area in northwest China. Vaccine 2012;30:7118–22. doi:10.1016/j.vaccine.2012.09.031

49 Li L, Zou H, Xu M, et al. Risk factors related to postpartum hepatic inflammation in pregnant women with chronic hepatitis B. J Int Med Res 2020;48:030006052096643. doi:10.1177/0300060520966439

50 Li Y, Wang J, Yu Y, et al. Maternal antiviral treatment safeguards infants from hepatitis B transmission in contingencies of delayed immunoprophylaxis. Liver International 2020;40:2377–84. doi:10.1111/liv.14479

51 Li Z, Xie Z, Ni H, et al. Mother-to-child transmission of hepatitis B virus: Evolution of hepatocellular carcinoma-related viral mutations in the post-immunization era. Journal of Clinical Virology 2014;61:47–54. doi:10.1016/j.jcv.2014.06.010

52 Liu J, Chen T, Chen Y, et al. 2019 Chinese Clinical Practice Guidelines for the Prevention of Mother-to-child Transmission of Hepatitis B Virus. J Clin Transl Hepatol 2020;8:397–406. doi:10.14218/JCTH.2020.00070

53 Lu L-L, Chen B-X, Wang J, et al. Maternal transmission risk and antibody levels against hepatitis B virus e antigen in pregnant women. International Journal of Infectious Diseases 2014;28:41–4. doi:10.1016/j.ijid.2014.07.028

54 Lu Y, Zhu F-C, Liu J-X, et al. The maternal viral threshold for antiviral prophylaxis of perinatal hepatitis B virus transmission in settings with limited resources: A large prospective cohort study in China. Vaccine 2017;35:6627–33. doi:10.1016/j.vaccine.2017.10.032

55 Michitaka K, Hiraoka A, Imai Y, et al. Clinical Features and Hepatitis B Virus (HBV) Genotypes in Pregnant Women Chronically Infected with HBV. Internal Medicine 2012;51:3317–22. doi:10.2169/internalmedicine.51.8596

56 Nishimura K, Yamana K, Fukushima S, et al. Comparison of Two Hepatitis B Vaccination Strategies Targeting Vertical Transmission: A 10-Year Japanese Multicenter Prospective Cohort Study. Vaccines 2021;9:58. doi:10.3390/vaccines9010058

57 Pan CQ, Zou H-B, Chen Y, et al. Cesarean Section Reduces Perinatal Transmission of Hepatitis B Virus Infection From Hepatitis B Surface Antigen–Positive Women to Their Infants. Clinical Gastroenterology and Hepatology 2013;11:1349–55. doi:10.1016/j.cgh.2013.04.026

58 Peng T-T, Cai Q-E, Yang M, et al. Epidemiological trends and virological traits of hepatitis B virus infection in pregnant women and neonates. Arch Virol 2019;164:1335–41. doi:10.1007/s00705-019-04190-4

59 Sasagawa Y, Yamada H, Morizane M, et al. Hepatitis B virus infection: Prevention of mother-to-child transmission and exacerbation during pregnancy. Journal of Infection and Chemotherapy 2019;25:621–5. doi:10.1016/j.jiac.2019.03.014

60 Ségéral O, S. N'Diaye D, Prak S, et al. Usefulness of a serial algorithm of HBsAg and HBeAg rapid diagnosis tests to detect pregnant women at risk of HBV mother-to-child transmission in Cambodia, the ANRS 12328 pilot study. Journal of Clinical Virology 2018;109:29–34. doi:10.1016/j.jcv.2018.10.007

61 Segeral O, Dim B, Durier C, et al. Immunoglobulin-free strategy to prevent HBV mother-to-child transmission in Cambodia (TA-PROHM): a single-arm, multicentre, phase 4 trial. The Lancet Infectious Diseases 2022;22:1181–90. doi:10.1016/S1473-3099(22)00206-7

62 Thilakanathan C, Wark G, Maley M, et al. Mother-to-child transmission of hepatitis B: Examining viral cut-offs, maternal HBsAg serology and infant testing. Liver International 2018;38:1212–9. doi:10.1111/liv.13736

63 Wang C, Wang C, Jia Z-F, et al. Protective effect of an improved immunization practice of mother-to-infant transmission of hepatitis B virus and risk factors associated with immunoprophylaxis failure. Medicine 2016;95:e4390. doi:10.1097/MD.0000000000004390

64 Xu Y-Y, Liu H-H, Zhong Y-W, et al. Peripheral Blood Mononuclear Cell Traffic Plays a Crucial Role in Mother-to-Infant Transmission of Hepatitis B Virus. Int J Biol Sci 2015;11:266–73. doi:10.7150/ijbs.10813

65 Zhang L, Gui X, Fan J, et al. Breast feeding and immunoprophylaxis efficacy of mother-to-child transmission of hepatitis B virus. J Matern Fetal Neonatal Med 2014;27:182–6. doi:10.3109/14767058.2013.806901

66 Zhang Z, Li A, Xiao X. Risk factors for intrauterine infection with hepatitis B virus. International Journal of Gynecology & Obstetrics 2014;125:158–61. doi:10.1016/j.ijgo.2013.10.028

67 Xu C, Liu J, Liu L, et al. Comparison of hepatitis B viral loads and viral antigen levels in child-bearing age women with and without pregnancy. BMC Pregnancy and Childbirth 2018;18:292. doi:10.1186/s12884-018-1932-9

68 Kfutwah AK, Tejiokem MC, Njouom R. A low proportion of HBeAg among HBsAg-positive pregnant women with known HIV status could suggest low perinatal transmission of HBV in Cameroon. Virology Journal 2012;9:62. doi:10.1186/1743-422X-9-62

69 Rouet F, Chaix M-L, Inwoley A, et al. HBV and HCV prevalence and viraemia in HIV-positive and HIV-negative pregnant women in Abidjan, Côte d'Ivoire: The ANRS 1236 study. Journal of Medical Virology 2004;74:34–40. doi:10.1002/jmv.20143

70 Thumbiran NV, Moodley D, Parboosing R, et al. Hepatitis B and HIV co-infection in pregnant women: Indication for routine antenatal hepatitis B virus screening in a high HIV prevalence setting. S Afr Med J 2014;104:307. doi:10.7196/SAMJ.7299

71 Keel P, Edwards G, Flood J, et al. Assessing the impact of a nurse-delivered home dried blood spot service on uptake of testing for household contacts of hepatitis B-infected pregnant women across two London trusts. Epidemiol Infect 2016;144:2087–97. doi:10.1017/S0950268815003325

72 Aba HO, Aminu M. Seroprevalence of hepatitis B virus serological markers among pregnant Nigerian women. Ann Afr Med 2016;15:20–7. doi:10.4103/1596-3519.172555

73 Anaedobe CG, Fowotade A, Omoruyi C, et al. Prevalence, sociodemographic features and risk factors of Hepatitis B virus infection among pregnant women in Southwestern Nigeria. Pan Afr Med J 2015;20. doi:10.11604/pamj.2015.20.406.6206

74 Andersson MI, Maponga TG, Ijaz S, et al. The epidemiology of hepatitis B virus infection in HIV-infected and HIV-uninfected pregnant women in the Western Cape, South Africa. Vaccine 2013;31:5579–84. doi:10.1016/j.vaccine.2013.08.028

75 Andreotti M, Pirillo MF, Liotta G, et al. The impact of HBV or HCV infection in a cohort of HIV-infected pregnant women receiving a nevirapine-based antiretroviral regimen in Malawi. BMC Infectious Diseases 2014;14:180. doi:10.1186/1471-2334-14-180

76 Chasela CS, Kourtis AP, Wall P, et al. Hepatitis B virus infection among HIV-infected pregnant women in Malawi and transmission to infants. Journal of Hepatology 2014;60:508–14. doi:10.1016/j.jhep.2013.10.029

77 Frempong MT, Ntiamoah P, Annani-Akollor ME, et al. Hepatitis B and C infections in HIV-1 and non-HIV infected pregnant women in the Brong-Ahafo Region, Ghana. PLoS ONE 2019;14:e0219922. doi:10.1371/journal.pone.0219922

78 Matthews PC, Beloukas A, Malik A, et al. Prevalence and Characteristics of Hepatitis B Virus (HBV) Coinfection among HIV-Positive Women in South Africa and Botswana. PLoS ONE 2015;10:e0134037. doi:10.1371/journal.pone.0134037

79 Rajbhandari R, Barton K, Juncadella AC, et al. Discontinuity of care for mothers with chronic hepatitis B diagnosed during pregnancy. Journal of Viral Hepatitis 2016;23:561–8. doi:10.1111/jvh.12524

80 van Schalkwyk J, Nourmoussavi M, Massey A, et al. Missed Opportunities for Prevention of Perinatal Transmission of Hepatitis B: A Retrospective Cohort Study. Canadian Journal of Gastroenterology and Hepatology 2014;28:525–8. doi:10.1155/2014/549764

81 Ahmadinejad Z, Abdi Liae Z, Salehizadeh S, et al. Efficacy of Post-Exposure Prophylaxis in Infants Born to HBsAg Positive Mothers in Iran; Is It Authentic? Iran J Pediatr 2016;In Press. doi:10.5812/ijp.5979

82 Hannachi N, Bahri O, Mhalla S, et al. Hépatite virale B chez les femmes enceintes tunisiennes : facteurs de risque et intérêt de l'étude de la réplication virale en cas d'antigène HBe négatif. Pathologie Biologie 2009;57:e43–7. doi:10.1016/j.patbio.2008.04.017

83 Hannachi N, Bahri O, ben fredj N, et al. [Risk of vertical transmission of hepatitis B virus in Tunisia]. Archives de l'Institut Pasteur de Tunis 2010;87:17–24.

84 Sbiti M, Khalki H, Benbella I, et al. Séroprévalence de l'AgHBs chez la femme enceinte dans le centre du Maroc. Pan Afr Med J 2016;24. doi:10.11604/pamj.2016.24.187.9849

85 Zahran KM, Badary MS, Agban MN, et al. Pattern of hepatitis virus infection among pregnant women and their newborns at the Women's Health Center of Assiut University, Upper Egypt. International Journal of Gynecology & Obstetrics 2010;111:171–4. doi:10.1016/j.ijgo.2010.06.013

86 Denis F, Ranger-Rogez S, Alain S, et al. Sreening of pregnant women for hepatitis B markers in a French Provincial University Hospital (Limoges) during 15 years. Eur J Epidemiol 2004;19:973–8. doi:10.1007/s10654-004-5755-9

87 Dervisevic S, Ijaz S, Chaudry S, et al. Non-A Hepatitis B Virus Genotypes in Antenatal Clinics, United Kingdom. Emerg Infect Dis 2007;13:1689–93. doi:10.3201/eid1311.070578

88 Elefsiniotis I, Tsoumakas K, Vezali E, et al. Spontaneous preterm birth in women with chronic hepatitis B virus infection. International Journal of Gynecology & Obstetrics 2010;110:241–4. doi:10.1016/j.ijgo.2010.04.020

89 Harder KM, Cowan S, Eriksen MB, et al. Universal screening for hepatitis B among pregnant women led to 96% vaccination coverage among newborns of HBsAg positive mothers in Denmark. Vaccine 2011;29:9303–7. doi:10.1016/j.vaccine.2011.10.028

90 Sagnelli E, Taliani G, Castelli F, et al. Chronic HBV infection in pregnant immigrants: a multicenter study of the Italian Society of Infectious and Tropical Diseases. New Microbiol 2016;39:114–8.

91 Söderström A, Norkrans G, Lindh M. Hepatitis B virus DNA during pregnancy and post partum: aspects on vertical transmission. Scand J Infect Dis 2003;35:814–9. doi:10.1080/00365540310016547

92 Ter Borg MJ, Leemans WF, De Man RA, et al. Exacerbation of chronic hepatitis B infection after delivery. Journal of Viral Hepatitis 2008;15:37–41. doi:10.1111/j.1365-2893.2007.00894.x

93 Banerjee A, Chakravarty R, Mondal P. HEPATITIS B VIRUS GENOTYPE D INFECTION AMONG ANTENATAL PATIENTS ATTENDING A MATERNITY HOSPITAL IN CALCUTTA, INDIA: ASSESSMENT OF INFECTIVITY STATUS. SOUTHEAST ASIAN J TROP MED PUBLIC HEALTH 2005;36:4.

94 Gumilar Dachlan E, Nugraheni C, Rahniayu A, et al. Quantitative HBsAg and Qualitative HBeAg Predicts Intrauterine Placental Infection and Umbilical Blood Cord in

Pregnant Women. JFRH Published Online First: 7 October 2020. doi:10.18502/jfrh.v14i2.4353

95 Dwivedi M, Misra S, Misra V, et al. Seroprevalence of hepatitis B infection during pregnancy and risk of perinatal transmission. Indian journal of gastroenterology : official journal of the Indian Society of Gastroenterology 2011;30:66–71. doi:10.1007/s12664-011-0083-y

96 Pande C, Sarin SK, Patra S, et al. Hepatitis B vaccination with or without hepatitis B immunoglobulin at birth to babies born of HBsAg-positive mothers prevents overt HBV transmission but may not prevent occult HBV infection in babies: a randomized controlled trial. Journal of Viral Hepatitis 2013;20:801–10. doi:10.1111/jvh.12102

97 Sirilert S, Khamrin P, Kumthip K, et al. Placental infection of hepatitis B virus among Thai pregnant women: Clinical risk factors and its association with fetal infection. Prenatal Diagnosis 2020;40:380–6. doi:10.1002/pd.5628

98 Wibowo AP, Masadah R, Nelwana BJ, et al. Asialoglycoprotein receptor expression in placenta of women with Hepatitis B Virus e Antigen (HBeAg) positive and negative. Enfermería Clínica 2020;30:255–8. doi:10.1016/j.enfcli.2019.10.017

99 Chen H-L, Lin L-H, Hu F-C, et al. Effects of maternal screening and universal immunization to prevent mother-to-infant transmission of HBV. Gastroenterology 2012;142:773-781.e2. doi:10.1053/j.gastro.2011.12.035

100 Chen J, Yan L, Zhu F-C, et al. Amino acid polymorphism in the reverse transcriptase region of hepatitis B virus and the relationship with nucleos(t)ide analogues treatment for preventing mother-to-infant transmission. Journal of Medical Virology 2014;86:1288–95. doi:10.1002/jmv.23948

101 Chen X, Chen J, Wen J, et al. Breastfeeding Is Not a Risk Factor for Mother-to-Child Transmission of Hepatitis B Virus. PLoS ONE 2013;8:e55303. doi:10.1371/journal.pone.0055303

102 Ding Y, Sheng Q, Ma L, et al. Chronic HBV infection among pregnant women and their infants in Shenyang, China. Virology Journal 2013;10:17. doi:10.1186/1743-422X-10-17

103 Giles M, Visvanathan K, Lewin S, et al. Clinical and virological predictors of hepatic flares in pregnant women with chronic hepatitis B. Gut 2015;64:1810–5. doi:10.1136/gutjnl-2014-308211

104 Guo J, Gao Y, Guo Z, et al. Frequencies of dendritic cells and Toll-like receptor 3 in neonates born to HBsAg-positive mothers with different HBV serological profiles. Epidemiology & Infection 2015;143:62–70. doi:10.1017/S0950268814000624

105 Huang H, Xu C, Liu L, et al. Increased Protection of Earlier Use of Immunoprophylaxis in Preventing Perinatal Transmission of Hepatitis B Virus. Clinical Infectious Diseases 2021;73:e3317–23. doi:10.1093/cid/ciaa898

106 Kim JH, Kim JS, Lee JJ, et al. Survey of perinatal hepatitis B virus transmission after Korean National Prevention Program in a tertiary hospital. Korean J Intern Med 2014;29:307. doi:10.3904/kjim.2014.29.3.307

107 Komatsu H, Inui A, Umetsu S, et al. Evaluation of the G145R Mutant of the Hepatitis B Virus as a Minor Strain in Mother-to-Child Transmission. PLoS ONE 2016;11:e0165674. doi:10.1371/journal.pone.0165674

108 Lao T, Leung T, Chan H, et al. Effect of pregnancy on the activity and infectivity of hepatitis B virus in women with chronic hepatitis B infection. Hong Kong medical journal = Xianggang yi xue za zhi / Hong Kong Academy of Medicine 2015;21:S4–7.

109 Li X-M. Interruption of HBV intrauterine transmission: A clinical study. WJG 2003;9:1501. doi:10.3748/wjg.v9.i7.1501

110 Lin X, Guo Y, Zhou A, et al. Immunoprophylaxis Failure Against Vertical Transmission of Hepatitis B Virus in the Chinese Population: A Hospital-based Study and a Meta-analysis. The Pediatric Infectious Disease Journal 2014;33:897. doi:10.1097/INF.0000000000000315

111 Liu C-P, Zeng Y-L, Zhou M, et al. Factors Associated with Mother-to-child Transmission of Hepatitis B Virus Despite Immunoprophylaxis. Intern Med 2015;54:711–6. doi:10.2169/internalmedicine.54.3514

112 Liu J, Wang J, Qi C, et al. Baseline Hepatitis B Virus Titer Predicts Initial Postpartum Hepatic Flare: A Multicenter Prospective Study. Journal of Clinical Gastroenterology 2018;52:902–7. doi:10.1097/MCG.0000000000000877

113 Lv N, Chu X-D, Sun Y-H, et al. Analysis on the outcomes of hepatitis B virus perinatal vertical transmission: nested case–control study. European Journal of Gastroenterology & Hepatology 2014;26:1286. doi:10.1097/MEG.0000000000000189

114 Peng S, Wan Z, Liu T, et al. Cesarean section reduces the risk of early mother-to-child transmission of hepatitis B virus. Digestive and Liver Disease 2018;50:1076–80. doi:10.1016/j.dld.2018.05.004

115 Qiao Y-P, Su M, Song Y, et al. Outcomes of the national programme on prevention of mother-to-child transmission of hepatitis B virus in China, 2016–2017. Infectious Diseases of Poverty 2019;8:65. doi:10.1186/s40249-019-0576-y

116 Shao Z-J, Zhang L, Xu J-Q, et al. Mother-to-infant transmission of hepatitis B virus: A Chinese experience. Journal of Medical Virology 2011;83:791–5. doi:10.1002/jmv.22043

117 Sheng Q-J, Wang S-J, Wu Y-Y, et al. Hepatitis B virus serosurvey and awareness of mother-to-child transmission among pregnant women in Shenyang, China: An observational study. Medicine 2018;97:e10931. doi:10.1097/MD.0000000000010931

118 Shi X, Wang X, Xu X, et al. Impact of HBV replication in peripheral blood mononuclear cell on HBV intrauterine transmission. Front Med 2017;11:548–53. doi:10.1007/s11684-017-0597-5

119 Su H-X, Zhang Y-H, Zhang Z-G, et al. High Conservation of Hepatitis B Virus Surface Genes during Maternal Vertical Transmission despite Active and Passive Vaccination. Intervirology 2011;54:122–30. doi:10.1159/000319437

120 Wang DD, Yi LZ, Wu LN, et al. Relationship between Maternal PBMC HBV cccDNA and HBV Serological Markers and its Effect on HBV Intrauterine Transmission. Biomed Environ Sci 2019;32:315–23. doi:10.3967/bes2019.043

121 Wang J-S. Transformation of hepatitis B serologic markers in babies born to hepatitis B surface antigen positive mothers. WJG 2005;11:3582. doi:10.3748/wjg.v11.i23.3582

122 Wang L, Wiener J, Bulterys M, et al. Hepatitis B Virus (HBV) Load Response to 2 Antiviral Regimens, Tenofovir/Lamivudine and Lamivudine, in HIV/ HBV-Coinfected Pregnant Women in Guangxi, China: The Tenofovir in Pregnancy (TiP) Study. J Infect Dis 2016;214:1695–9. doi:10.1093/infdis/jiw439

123 Wang Z, Zhang J, Yang H, et al. Quantitative analysis of HBV DNA level and HBeAg titer in hepatitis B surface antigen positive mothers and their babies: HBeAg passage through the placenta and the rate of decay in babies. Journal of Medical Virology 2003;71:360–6. doi:10.1002/jmv.10493

124 Wiseman E, Fraser MA, Holden S, et al. Perinatal transmission of hepatitis B virus: an Australian experience. Medical Journal of Australia 2009;190:489–92. doi:10.5694/j.1326-5377.2009.tb02524.x

125 Wu K, Wang H, Li S, et al. Maternal hepatitis B infection status and adverse pregnancy outcomes: a retrospective cohort analysis. Arch Gynecol Obstet 2020;302:595–602. doi:10.1007/s00404-020-05630-2

126 Xu D-Z, Yan Y-P, Choi BCK, et al. Risk factors and mechanism of transplacental transmission of hepatitis B virus: a case-control study. J Med Virol 2002;67:20–6. doi:10.1002/jmv.2187

127 Yi W, Pan CQ, Li M-H, et al. The characteristics and predictors of postpartum hepatitis flares in women with chronic hepatitis B: American Journal of Gastroenterology 2018;113:686–93. doi:10.1038/s41395-018-0010-2

128 Yin Y, Wu L, Zhang J, et al. Identification of risk factors associated with immunoprophylaxis failure to prevent the vertical transmission of hepatitis B virus. Journal of Infection 2013;66:447–52. doi:10.1016/j.jinf.2012.12.008

129 Yonghao G, Pumei D, Jianhui Y, et al. A retrospective study of hepatitis B mother-to-child transmission prevention and postvaccination serological test results of infants at risk of perinatal transmission in two counties of middle China. Journal of Viral Hepatitis 2017;24:687–95. doi:10.1111/jvh.12694

130 Liu J, Zhang S, Liu M, et al. Maternal pre-pregnancy infection with hepatitis B virus and the risk of preterm birth: a population-based cohort study. The Lancet Global Health 2017;5:e624–32. doi:10.1016/S2214-109X(17)30142-0

131 Zhu Y-Y, Mao Y-Z, Wu W-L, et al. Does Hepatitis B Virus Prenatal Transmission Result in Postnatal Immunoprophylaxis Failure? Clin Vaccine Immunol 2010;17:1836–41. doi:10.1128/CVI.00168-10
